# Tuning light-driven oxidation of styrene inside water-soluble nanocages

**DOI:** 10.1038/s41467-024-45991-9

**Published:** 2024-02-28

**Authors:** Souvik Ghosal, Ankita Das, Debojyoti Roy, Jyotishman Dasgupta

**Affiliations:** Department of Chemical Sciences, Tata Institute of Fundamental Research, 1 Homi Bhabha Road, Mumbai, 400005 India

**Keywords:** Photocatalysis, Photocatalysis, Catalyst synthesis

## Abstract

Selective functionalization of innate sp^2^ C-H bonds under ambient conditions is a grand synthetic challenge in organic chemistry. Here we combine host-guest charge transfer-based photoredox chemistry with supramolecular nano-confinement to achieve selective carbonylation of styrene by tuning the dioxygen concentration. We observe exclusive photocatalytic formation of benzaldehyde under excess O_2_ (>1 atm) while Markovnikov addition of water produced acetophenone in deoxygenated condition upon photoexcitation of confined styrene molecules inside a water-soluble cationic nanocage. Further by careful tuning of the nanocage size, electronics, and guest preorganization, we demonstrate rate enhancement of benzaldehyde formation and a complete switchover to the anti-Markovnikov product, 2-phenylethan-1-ol, in the absence of O_2_. Raman spectroscopy, 2D ^1^H-^1^H NMR correlation experiments, and transient absorption spectroscopy establish that the site-selective control on the confined photoredox chemistry originates from an optimal preorganization of styrene molecules inside the cavity. We envision that the demonstrated host-guest charge transfer photoredox paradigm in combination with green atom-transfer reagents will enable a broad range of sp^2^ carbon-site functionalization.

## Abstract (web summary)

Selective functionalization of C(sp2)–H bonds under ambient conditions is a persistent synthetic challenge in organic chemistry. Here the authors combine host-guest charge-transfer-based photoredox chemistry with supramolecular nano-confinement to achieve selective carbonylation of styrene by tuning the dioxygen concentration.

## Introduction

Oxidative olefin functionalization for regioselective C-O or C-C bond formation has been a fundamental chemical challenge in the synthesis of diverse natural products and target pharmaceuticals^1–7^. Selective chemistry is usually hindered by the lack of intrinsic polarity in olefinic C = C π-bond. Traditional approaches to circumvent the polarity problem involve the incorporation of electron donating or withdrawing functionality in the olefin moiety by substrate pre-functionalization. The use of transition metal complexes additionally offers a synergistic effect on the olefin site by perturbing the C = C bonding strength either through effective metal-π-complexation or via back-donation to the anti-bonding π*-orbitals^4,6–20^. Recently, the advent of photoredox catalysis has revolutionized the field of olefinic functionalization where visible photons act as reagents for ultrafast bond activation and provide unexplored avenues for harnessing new reactivity^10,21–28^. The electron transfer or energy transfer processes mediated by an excited photocatalyst enables chemical transformation of olefins through the reactive radical-cation state or through the photo-excited triplet state intermediates of the substrate. However, most of these activation reactions are diffusion-limited in the free solution, and hence the selectivity gets limited by the intrinsic electronic and steric influences of the olefin substrates.

Pre-functionalization through attachment of a removable covalent template to the substrate can alleviate the selectivity challenge although the additional chemical steps of template attachment and removal compromise the atom-economy of the synthetic steps^8,15^. Alternately, preorganization of substrate with respect to reagents through designed non-covalent interactions inside a nanocavity can generate the necessary anisotropic environment to tune the product selectivity mimicking the enzyme active-site^29–45^. Such a catalytic strategy can give rise to product distributions that may be complementary to that obtained in bulk solution^29^. In the last few years, it has been shown that substrates inside supramolecular confinement can be photo-activated^46,47^ at ultrafast timescales^48^ and catalytic chemistry^49^ can be carried out utilizing the nanocavity either as a silent spectator^42,50–52^ or as an active photoredox partner^48,49,53^. Our approach relies on the formation of a photoactivable host-guest charge transfer (CT) complex upon substrate incarceration inside a redox complementary supramolecular cavity. Photoexcitation at the host-guest CT band drives selective bond functionalization via photo-induced electron transfer reaction and optimal pre-organization of the confined reactants without the need of any external photosensitizer.

**Fig. 1:**
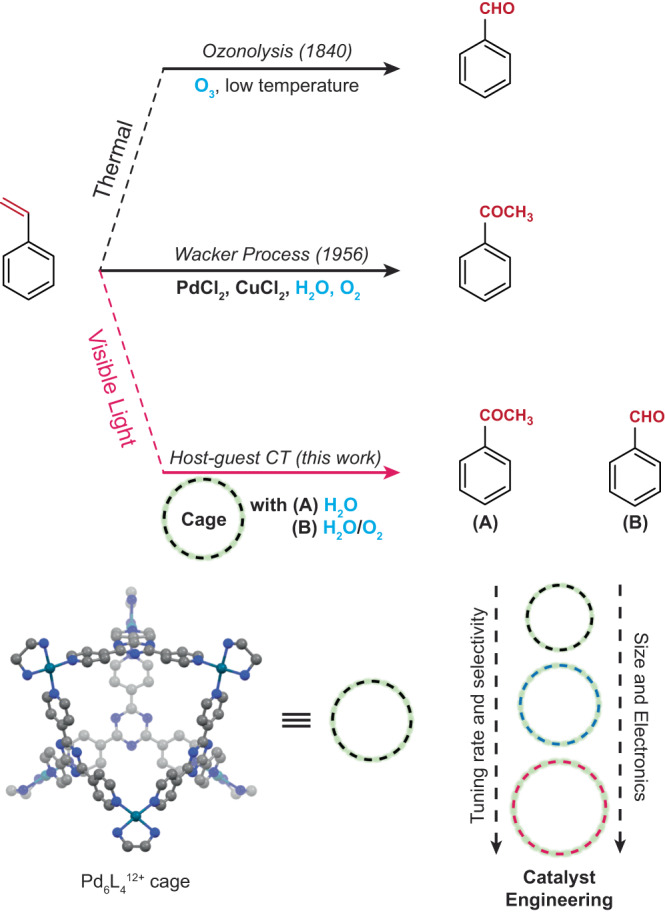
Strategies to carry out sp^2^ C-H bond functionalization at the olefin site on styrene. Classically ozonolysis and Wacker process have been used to make aldehydes and ketones from olefins by requisite oxidants in the ground state. The methodology described in this work uses host-guest CT paradigm to open an alternate route through the excited state manifold with visible light as reagent. Crystal structure representation of Pd_6_L_4_^12+^ nanocavity was taken from CCDC-1271853.

Traditionally, oxidation of simple aromatic olefins like styrene has been carried out by classical methods like Wacker process^54^ and ozonolysis^55^ that exploit the ground state reactivity of the olefin bond (Fig. 1). Supramolecular encapsulation of styrene and its derivatives leading to confined oxidation has previously been demonstrated although no light-mediated method has been reported thus far^50,51,56,57^. Using our host-guest CT paradigm, here we demonstrate light-induced oxidative functionalization of the olefinic sp^2^ C-H bond of styrene and its derivatives while they are confined inside an electron-deficient water-soluble cationic Pd_6_L_4_^12+^ nanocage^29^. The olefinic bond of styrene is partially weakened in the ground state by non-covalent host-guest interactions. Subsequent photoexcitation of the host-guest CT complex leads to an efficient electron transfer from styrene to the nanocage, thereby driving subsequent oxidation reaction of styrene with either pre-organized water or dissolved dioxygen gas to form regioselective C-O bonds. We report that modulating the O_2_ pressure tunes the kinetic partitioning between the Wacker product, acetophenone and the ozonolysis product, benzaldehyde. Alteration of the external ancillary amine ligands that coordinate to the Pd^2+^ site subtly tunes the regioselectivity of C-O bond formation and yield of C-C coupling reactions under ambient condition. Under Argon atmosphere, we however observe a remarkable switch of regioselectivity from Markovnikov to anti-Markovnikov product upon changing the remote Pd-capping ligand and consequent cage-size which highlights the power of confinement-mediated preorganization. Upon increasing the size of the confinement, we accelerate the rate of photocatalytic styrene oxidation at high O_2_ pressure. Thus, we demonstrate a modular/ general photochemical route to tune both regio- and chemo-selectivity of olefin functionalization in styrene inside a water-soluble nanocavity.

## Results

### Pd_6_L_4_^12+^ Nanocage synthesis and host-guest complex characterization

The Pd_6_L_4_^12+^ nanocage (L = 2,4,6-tris(4-pyridyl)−1,3,5-triazine; **TPT**) was prepared using the reported protocol ^30^with slight modifications (Supplementary Fig. 1)^48,49,53^. Briefly, the synthesized precursor [Pd(**X**)(ONO_2_)_2_] metal-complexes [**X** = Ethylenediamine (En), Tetramethyl ethylenediamine (TMEDA) or Bipyridyl (BiPy)] were treated with organic ligand **TPT** in 3:2 molar-stoichiometry at 80 °C^48,53^ to obtain the corresponding nanocages and the purity was checked by ^1^H-NMR (Supplementary Figs. 6–8).

**Fig. 2:**
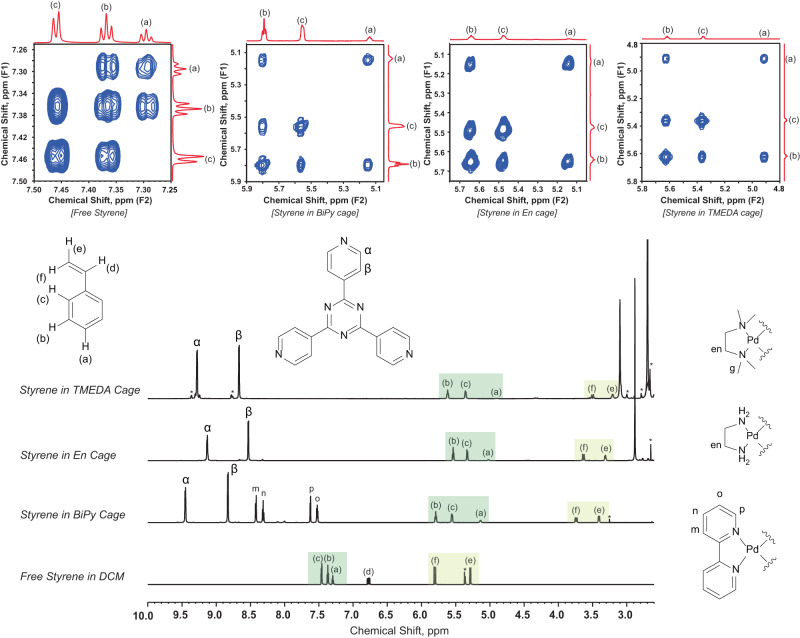
Comparative ^1^H NMR characterization of free styrene in organic solvent CD_2_Cl_2_ versus styrene-host complexes in D_2_O for three different Pd_6_L_4_^12+^ cavities indicating successful incarceration. (At the bottom) The stacked spectra demonstrate the differential shielding effects of different nanocavities on styrene, and also the different extent of upfield shifting for different protons of the same styrene molecule due to host-guest packing. All the styrene protons are labelled while the methyl (g) and methylene (en) protons associated with cage capping ligands are seen as singlets in between 2.8–3.2 ppm. (At the top) The effect was further cross-validated from different ^1^H-^1^H cross-correlation patterns observed for the styrene-host complexes in comparison to free styrene in 2D COSY spectra.

In order to incarcerate styrene and its derivatives inside the cavity, we first titrated the equivalents of styrene from stoichiometric concentration to molar excess up to 10 equivalents. Using ^1^H-NMR and DOSY experiments (Supplementary Figs. 12–13) we found that the optimal ratio is 10 equivalents of styrene per equivalent of nanocage for fully loading the cavity and also for a higher fraction of loaded cavity compared to the empty cavity. Therefore, we incubated 10 molar equivalents of guests in 2.5 mM solution of the Pd_6_L_4_^12+^ cage for 1 h and characterized the host-guest complex through ^1^H-NMR. Compared to the NMR spectrum of free styrene and its derivatives in organic solvent CD_2_Cl_2_, (Supplementary Figs. 9–11), we observed a prominent upfield shift for the cage-confined styrene peaks from 7.2–7.5 ppm (in free) to 4.6–6 ppm for the aromatic protons while the alkenyl protons moved upfield from 5.2–6.8 ppm (in free) to 3.8–3.1 ppm inside all the three cages indicating a more shielding hydrophobic environment inside the cavity (Section 1.2.3 in Supplementary Information and Fig. 2). Further, the NMR peak integration ratio suggests that a maximum of four styrene molecules on an average can get accommodated inside En-cage while the host-guest binding stoichiometry was 1:5 for both the TMEDA and BiPy cage due to the slightly larger cavity volume^58,59^. We also observed that the *meta*-H_b_ and *ortho*-H_c_ protons of styrene have exchanged their positions in the chemical shift axis in all three host-guest inclusion complexes revealing a more upfield shift of H_c_ with respect to H_b_ further verified by 2D COSY experiments (Fig. 2). This indicates that the alkenyl moiety of the guest molecule is facing more towards the hydrophobic triazine wall while the phenyl group is buried towards the center of the cage.

Further, to track the nature of guest incarceration dynamics inside the host and understand the host-guest interaction more deeply, we systematically titrated the guest to host molar ratio and monitored both ^1^H-NMR and DOSY (Diffusion-ordered spectroscopy) spectrum. When we take 1:1 ratio of the host and guest to start the incubation process with the aim to form a 1:1 host-guest complex, we observe that there are already ~3 styrene molecules per nanocage as seen in ^1^H NMR spectra (Supplementary Fig. 12). The cage:styrene stoichiometry saturates to ~1:4 even at a cage:styrene incubation molar ratio of 1:3 (Supplementary Fig. 12). Of course, the concentration of the loaded cage is certainly lesser than the normal case of 1:10 ratio of host-guest in the incubation process. Further, through the ^1^H-DOSY measurement, we measured the diffusion coefficient values of both cage protons and characteristic styrene protons for host-guest complexes obtained from mixing those at different incubation ratios (Supplementary Fig. 13). We observed that the cage protons showed a diffusion constant of 2.2 Å^2^/s with no styrene incubated while that shifts to 1.9 Å^2^/s when incubated with 1 eq. styrene (Supplementary Fig. 13). Thereafter, to our surprise, we observed no more significant change of diffusion constant values when more styrene was incubated with the cage (Supplementary Fig. 13). This led us to conclude that the binding of the guest inside the nanocage is most likely cooperative in nature. After characterization of the host-guest complex, we went ahead to check the photo-reactivity of styrene ⊂ host complexes.

### Photo-reactivity of host-guest complex

**Fig. 3:**
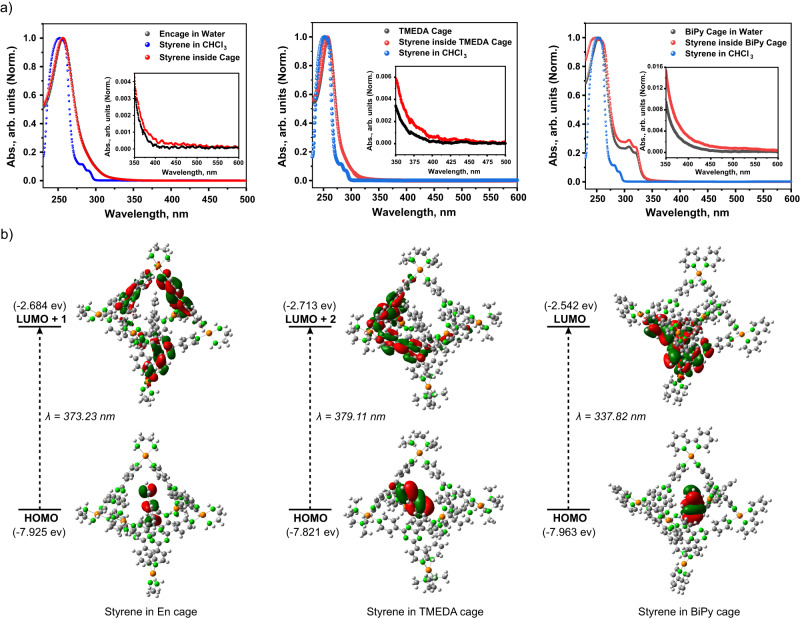
Optical transitions of host-guest CT complexes. **a** Steady-state absorption spectra of styrene-host complexes for three different hosts namely En cage, TMEDA cage, and BiPy cage: we observed a broadening at the 400 nm spectral region for the host-guest complexes which we predicted as the contribution from guest to host CT state absorptions. **b** Single-point TD-DFT calculations on styrene-host complexes predict prominent guest-to-host CT transitions.

In order to carry out photoreactions on styrene inside the nanocage, we first carried out steady-state UV-Vis absorption measurements for the styrene-cage host-guest complex and compared it to free styrene and empty cavity for all three different cages. We observe a subtle broadening in the spectrum at 400 nm region for all styrene-cage complexes (Fig. 3 and Supplementary Figs. 14–15) which indicates the presence of host-guest CT states. This broadening of the absorption features was assigned to charge transfer transitions based on the results obtained from the electronic structure calculations performed using time-dependent density functional theory. Calculations with CAM-B3LYP/ lanl2dz (Pd)/ 6-31 g* (C,H,O,N) level of theory were performed on geometry optimized host-guest complexes (Fig. 3, Supplementary Information Section 1.2.10 and Supplementary Figs. 16–22). The red-most transitions were described by the corresponding involved molecular orbitals which showed that the spectra between 350 to 450 nm can be explained by two different types of transitions: (1) host-centric MLCT (metal-to-ligand charge transfer) transitions (Supplementary Fig. 17), and (2) styrene-host CT-transitions (Fig. 3, Supplementary Figs. 16 and 19). The charge-transfer character for those transitions was characterized by the extent of electron-shift during the optical transition parametrized by D_CT_ factor^60–62^.

**Fig. 4:**
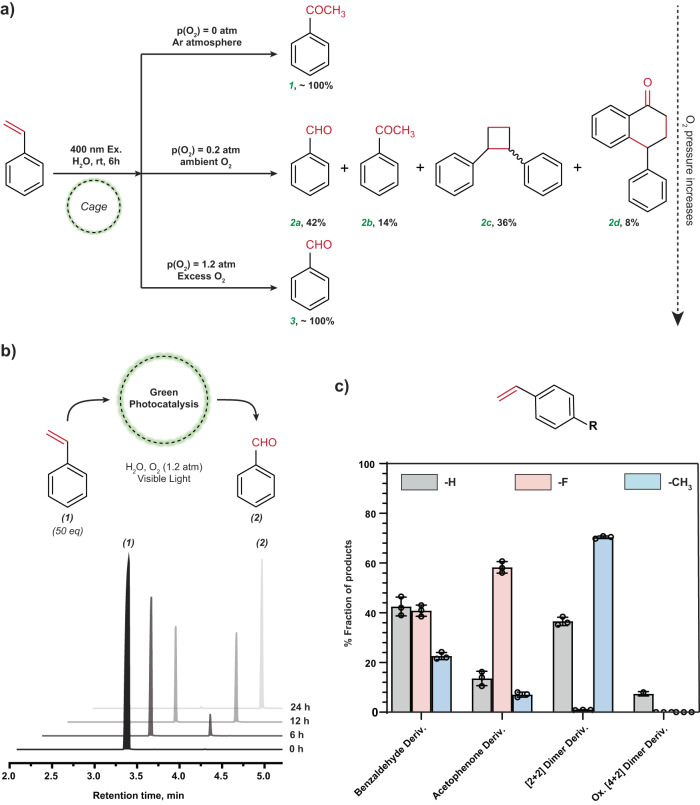
Photo-activated olefin functionalization of styrene inside Pd_6_L_4_^12+^ nanocage (En cage). **a** Oxygen-sensitive reactivity of styrene inside Encage and corresponding product distribution. **b** Representative gas chromatogram traces for photocatalytic conversion of styrene into benzaldehyde. **c** Tuning the selectivity of styrene photo-functionalization by introducing *para*-substituents. For 4-fluorostyrene, water addition-mediated acetophenone formation was observed to be the major pathway while for 4-methylstyrene, intermolecular C-C coupling-mediated dimer formation was observed to be the major reaction channel. Each experiment was replicated three times to generate the error bar.

Photoexcitation of the styrene-cage complex at 400 nm (30–40 mW/cm^2^) for 5.5 h, (Supplementary Fig. 5) under deoxygenated conditions yielded Wacker product acetophenone with 100% selectivity. The acetophenone product was characterized by ^1^H-NMR and GC-MS spectra (Fig. 4a and Supplementary Figs. 23–25). This remarkable result motivated us to systematically test the sensitivity of the photoreaction to O_2_ pressure. Under ambient air (0.2 atm), the photoreaction produced both benzaldehyde and acetophenone with additional [2 + 2]-cycloadduct ‘1,2-diphenylcyclobutane’ and another oxidized [4 + 2]-cycloadduct ‘4-phenyl-3,4- dihydronaphthalen-1(2H)-one’ respectively. At excess O_2_ pressure (1.2 atm) (5.8 molar equivalent with respect to styrene), exclusively benzaldehyde is formed. This seamless switchover from a Wacker product to an ozonolysis product demonstrates the significance of tuning the molecular O_2_ concentration in determining the chemoselectivity.

Apart from the role of O_2_ as an active reagent for these photoreactions in this framework, we also hypothesized that O_2_ could take away the electrons from the cage as a terminal oxidant, thereby making the cage a redox catalyst (based on our previous experience with similar host-guest chemistry)^49^. Therefore, we took 50 equivalents of styrene (125 mM) with respect to Encage concentration (2.5 mM) and after 1 h of incubation, we illuminated the solution with 400 nm LED light. We found that almost 100% conversion took place within 24 h. In Fig. 4b, representative gas chromatogram traces are plotted to depict the time course of the photo-catalytic conversion of styrene into benzaldehyde with a catalytic turnover of 50 at excess oxygen pressure of 1.2 atm i.e., 5.8 molar equivalents to styrene concentration (Supplementary Fig. 26). It unequivocally reveals that photocatalysis with these redox-amenable cages will be a universally feasible idea and it also opens up an alternate green yet selective route to achieve ozonolysis-equivalent products from aromatic olefins.

We hypothesized that styrene radical cation photo-generated through guest-to-host charge transfer in the excited state manifold can react with O_2_ at its higher pressure to generate benzaldehyde as the only photoproduct. While under anaerobic conditions, the photoexcited styrene radical cation can react only with the solvent water molecule followed by cage and water cluster mediated concerted electron-proton transfer steps to ultimately produce acetophenone. At intermediate pressures of O_2_ (from 0.2 to 1.2 atm) when the diffusion of the O_2_ inside the nanocage becomes a kinetically slow process, intermolecular coupling between one styrene radical cation and another neutral styrene molecule packed inside the same cage, can also generate the C-C coupled products depending on their relative orientations. In fact, we see that in pressures between 0.2 to 0.8 atm, we find a small amount of 2-phenyl-ethanol (Supplementary Fig. 27). We do not see this product either at high or low O_2_ partial pressures.

### Effect of substituents on the selectivity of styrene photoreaction

In order to probe the effect of substrate functionalization on the reaction selectivity, we systematically incarcerated different styrene derivatives (NMR shown in Supplementary Figs. 28–29) inside Encage for their subsequent photoreactions under ambient conditions (Fig. 4c, Supplementary Figs. 30–32). Incorporation of an electron-donating and hydrophobic functional group, like methyl at the para position of styrene enhances the relative yield of the [2 + 2] dimer (70.8%) while introducing an electron-withdrawing and hydrophilic functionality like- fluorine at the para position drastically reduces the relative yield of the dimer to ~1% (Supplementary Fig. 31). The major product from 4-methylstyrene, i.e., the corresponding [2 + 2] dimer was having the fastest rate of formation, while we obtained 4-fluoroacetophenone as the major product (58%) from 4-fluorostyrene with the fastest rate of formation (Fig. 4c, Supplementary Figs. 31–32). It clearly indicates that 4-methylstyrene molecules are comparatively more buried inside the Encage due to its hydrophobic functionality allowing facile guest orientation for [2 + 2] coupling reaction. The -F group makes 4-fluorostyrene molecules more exposed towards the water network through H-bonding interactions which favors the formation of acetophenone through water addition-oxidation cascade, and possibly disfavors the intermolecular coupling due to the lack of appropriate orientation for that reaction. The variation of product distribution strongly emphasizes the influence of both host-guest preorganization and substrate electronic structure in confinement induced photochemistry. This remarkable result demonstrates solvent exposure of the guest’s reactive site as a tuning parameter for the photo-selection in supramolecular confinement-based chemistry which one can modulate by introducing appropriate solvophilic or solvophobic functional groups into the guest molecules.

### Tuning the nanocage to photoswitch from Markovnikov to anti-Markovnikov addition reaction

**Fig. 5:**
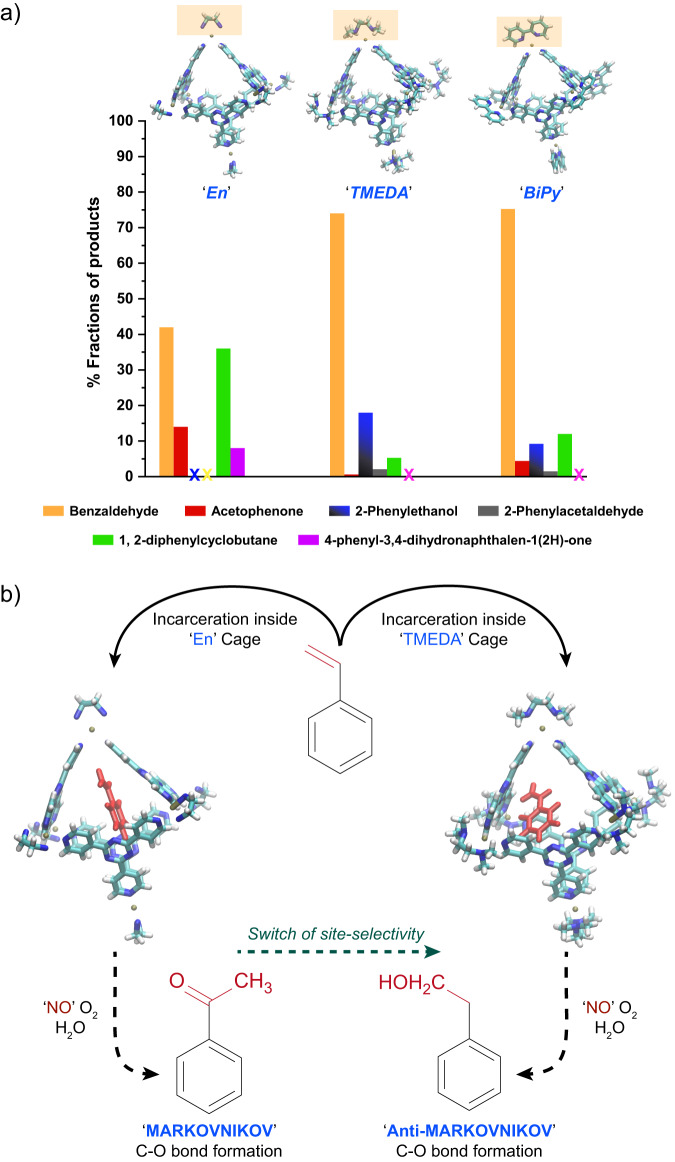
Tuning photo-selection of olefin functionalization of styrene by altering the nanocage structure. **a** Bar diagram of the altered product selectivity originated from the change in remote metal-capping ligands from ethylenediamine (En) to tetramethylethylenediamine (TMEDA) and bipyridine (BiPy). **b** Under inert (deoxygenated) condition, we observed a remarkable switch of product selectivity from exclusive formation of the Markovnikov product acetophenone to exclusive anti-Markovnikov product 2-phenylethanol just by altering the external ancillary ligand of the Pd_6_L_4_^12+^ nanocage from En to TMEDA.

The biomimetic host-guest complex here can behave like a photo-oxidase enzyme. To establish this, we modified the nanocage by altering the ancillary bidentate amine ligand from ethylenediamine (En) to tetramethyl ethylenediamine (TMEDA) and bipyridine (BiPy), respectively. The change in the Pd-ligand subtly changes the volume of the cavity which alters the styrene preorganization (seen in Fig. 2) as reflected from the altered product distribution. We performed photoreactions in these three styrene-nanocage complexes with 400 nm light-excitation at ambient condition and probed the photoproducts by GCMS studies. In Fig. 5a, under ambient O_2_, we found ~1.8 times enhancement in the benzaldehyde formation selectivity and a drastic decrease in [2 + 2] dimer formation in both TMEDA and BiPy cages compared to Encage. We observed significant 6-fold reduction in TMEDA cage and 3-fold reduction in BiPy cage for the formation of [2 + 2] dimeric products. For both TMEDA and BiPy nanocages, we observed a substantial decrease in Markovnikov adduct acetophenone correlated with the emergence of anti-Markovnikov adduct, 2-phenylethanol (Fig. 5, Supplementary Figs. 33–34). Subsequently, we performed photoreactions on styrene-TMEDA cage complex under stringent Ar atmosphere. Remarkably after 6 h of photoreaction, we obtained 88% conversion of styrene to yield 2-phenylethanol with ~100% selectivity, characterized by GCMS and ^1^H-NMR (Fig. 5b, Supplementary Figs. 35–36). This exciting result opens up an new light-activated synthetic route for forming anti-Markovnikov C-O bonds from sp^2^ C-H bond. Apart from the change in the site-selectivity of water addition, we observed an excellent control over the oxidation state of the final oxidized product as well. Water addition to the benzylic position of styrene subsequently undergoes further host-mediated photo-oxidation which ultimately leads to the formation of carbonyl functionality (acetophenone formation); while similar water addition to the terminal olefinic carbon of styrene leads to the formation of alcohol (2-phenylethanol), not to the aldehyde. The emergence of 2-phenylethanol without any further oxidation to the corresponding aldehyde indicates that the primary non-benzylic C-H does not undergo PCET due its poor acidity^48,49^.

Our preliminary ^1^H NMR data in Fig. 2 for all the host-guest complexes clearly suggest that a total of five styrene molecules on an average are packed inside TMEDA and BiPy cages while a total of four styrene molecules get incarcerated inside Encage. Hence, it indicates that altered packing of the styrene molecules enables more protrusion of the double-bond towards the cavity pores in TMEDA and BiPy cages compared to that in Encage. Therefore, the greater solvent accessibility allows lesser chances for inter-guest coupling in TMEDA and BiPy cages, further reflected in the product distribution at ambient condition for those cages. Based on this evidence of packing differences, we believe that the modulation of styrene packing inside different cages altered the positioning of reactive alkene-site with respect to the interfacial water-network. Consequently, the water addition takes place either at the terminal alkenyl carbon or at the buried alkenyl carbon next to the benzene ring. Furthermore, we observed that the rate of styrene consumption during ambient photo-oxidation inside Encage has an exponential behavior while it’s sigmoidal with a lag-phase in either TMEDA or BiPy cages (Supplementary Fig. 37).

**Fig. 6:**
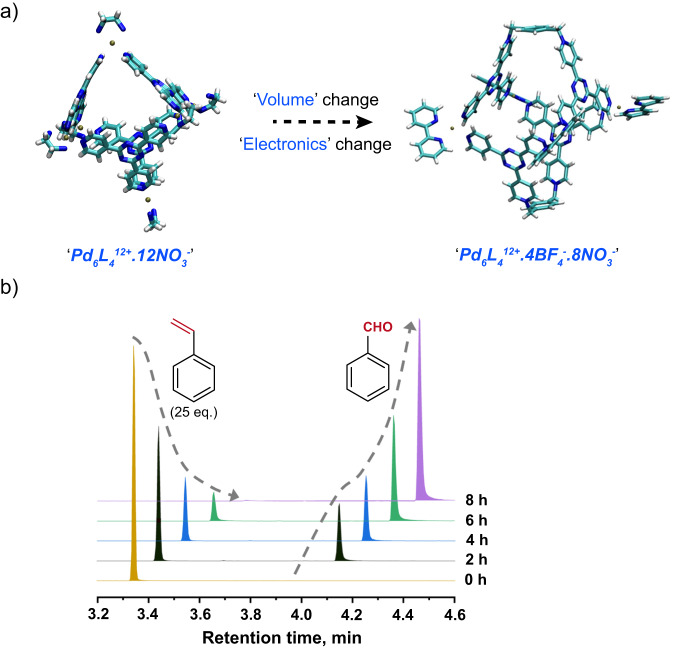
Alteration of pore-size leads to a faster photocatalytic turnover. **a** Switching to a larger (82% volume expansion) nanocavity having four Pd^2+^ sites and two triazine based cationic ligands leads to exclusive benzaldehyde formation even in the ambient condition and (**b**) a more efficient rate of photocatalytic turnover (TON of 25) to produce benzaldehyde from styrene within 8 h of 400 nm light illumination.

In order to increase the catalytic rates and specifically tune the substrate entry rates (k_ON_), we synthesized Pd_4_L’_2_^12+^.4BF_4_^-^.8NO_3_^-^ nanocage^31^ by separately synthesizing the [Pd(BiPy)(ONO_2_)_2_] complex and the organic ligand L’ (Supplementary Figs. 2–3, Section 1.2.2 in Supplementary Information) and characterized by ^1^H-NMR (Supplementary Figs. 38–40). Although this cage provides a larger pore size and cavity volume (82% larger in comparison to Encage), we surprisingly observed only 3–4 styrene molecules on average getting inside the cavity. While carrying out photocatalysis with this larger cage, we observed ~100% conversion of 25 equivalents styrene per nanocage (4 mol %) into benzaldehyde selectively, even at ambient condition within only 8 h (Fig. 6). We have earlier observed a catalytic turnover of 25 at 12 h under high O_2_ pressure for Encage. This unequivocally demonstrates the faster rate of photocatalysis probably due to a larger pore size and altered styrene preorganization in this larger cavity. Additionally, the absence of any water addition product indicates that either O_2_ has greater access to the olefin site of the reactants or water has lesser access towards the olefin site in this cavity, unlike the tighter packing of the guest molecules in the Pd_6_L_4_^12+^ cavity. This remarkable observation highlights the immense importance of host-guest preorganization as well as the pore size of the host for substrate entrance and product exit in the photocatalytic conversion of styrene.

### Mechanistic insights

**Fig. 7:**
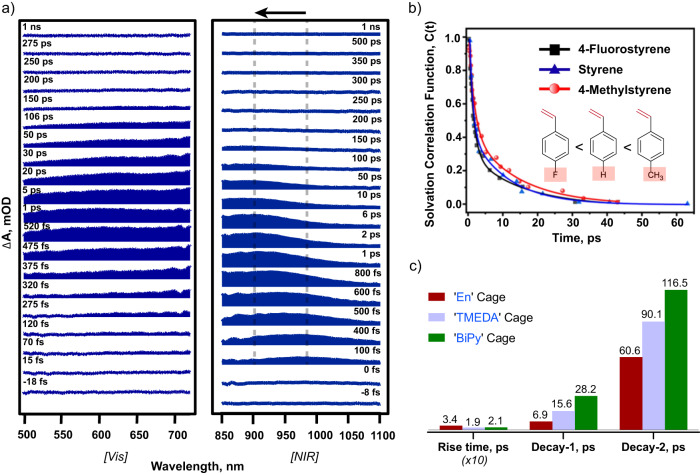
Ultrafast transient absorption probes the reactive host-guest CT state. **a** Transient absorption spectrum for styrene ⊂ Encage inclusion complex in both visible and the NIR spectral region. Literature report suggests that the broad absorption observed between 450 nm to 750 nm can be assigned to the styrene radical cation. The spectral feature appearing at the NIR region shows a blue-shift suggesting the effect of solvation dynamics due to formation of host-guest CT state. **b** Comparison of solvation dynamics for styrene and its derivatives inside Encage shows the fastest solvation dynamics for the 4-fluorostyrene ⊂ Encage complex while the 4-methylstyrene ⊂ Encage complex shows a slow solvation. The data reveals more solvent exposure for the 4-fluorostyrene while 4-methylstyrene is possibly the most buried inside the En cage. **c** The comparative lifetime components for NIR absorbing host-guest CT states for En, TMEDA and BiPy cages showing higher CT state lifetime of TMEDA and BiPy cages in comparison to En cage.

**Fig. 8:**
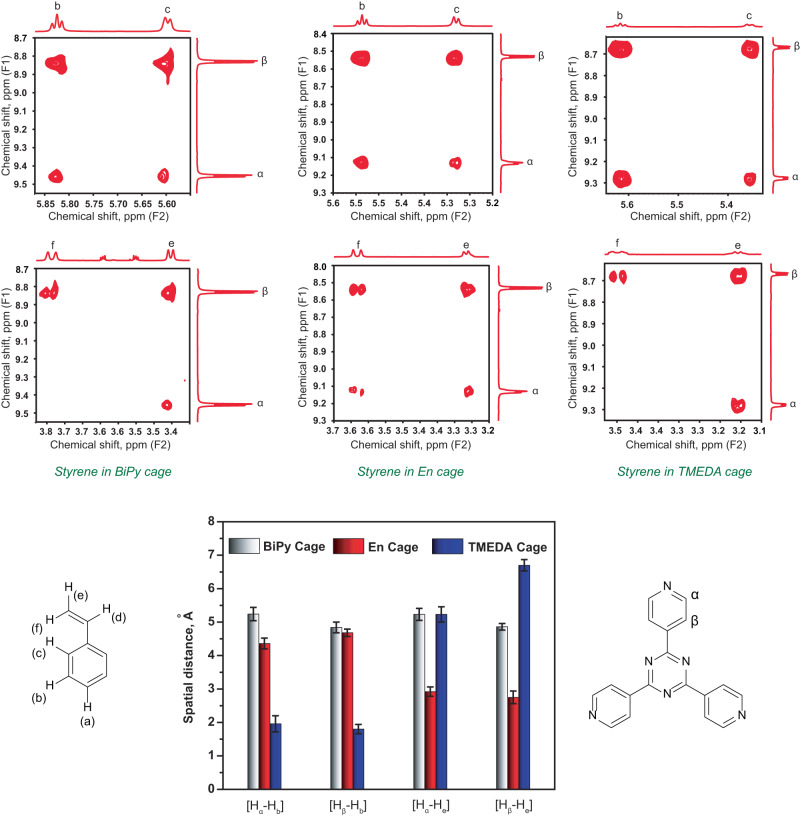
^1^H-^1^H through-space correlation measurement by ROESY probes differential host-guest packing for three different cages. The cross-peak intensities in between the host triazine protons and styrene protons obtained from the 2D ROESY NMR experiments are presented. For all the three host-guest complexes we extract the distance parameters between host protons and guest protons which probe the fundamental differences in host-guest packing parameters in three different cages. Each experiment was replicated two times to generate the error bar.

**Fig. 9:**
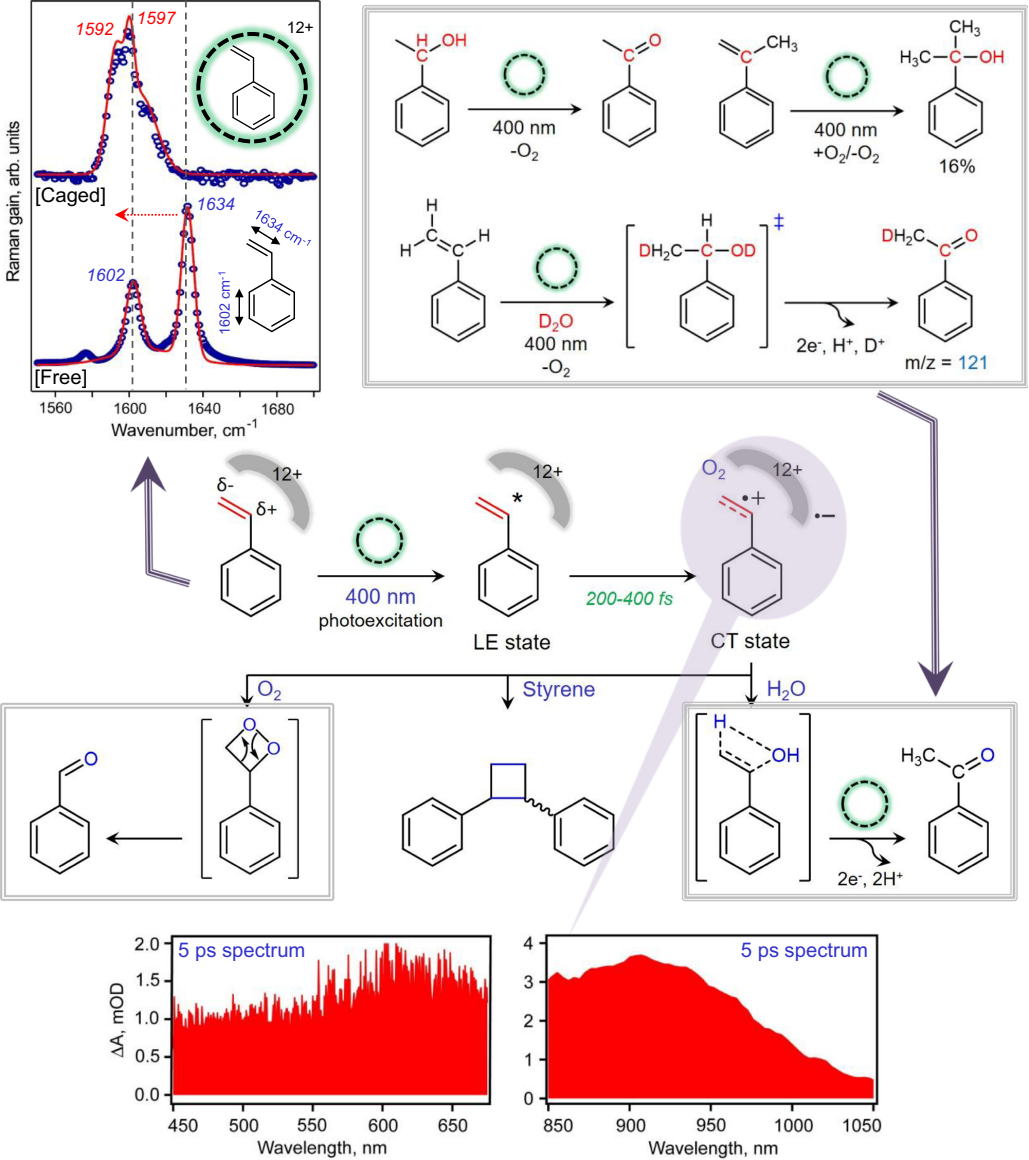
Probing “confinement effect” on selective bond polarization and light-activated host-guest charge transfer reaction. (Central Panel) A proposed mechanistic model for photo-oxidation of styrene molecules inside different hosts: the overall scheme for all possible reaction pathways after the formation of host-guest CT states is illustrated in the scheme with some important mechanistic investigations. (Top left panel) Steady state Raman spectral traces for free styrene (bottom, blue) and caged styrene (top, blue) along with the Gaussian fit (red line) obtained by an off-resonance excitation of styrene-En cage complex at 532 nm. (Top right panel) Chemical probes to understand the dark reaction steps for acetophenone formation which was believed to follow water addition followed by cage mediated photo-oxidation cascade pathway. First, our hypothesized alcohol intermediate gives acetophenone as single product both in presence and absence of O_2_. Further, the alcohol intermediate has been trapped in photo-oxidation of α-methylstyrene. Additionally, a linear enrichment of D-labeled product has been observed with gradual enhancement of D_2_O in the solvent system. (Bottom panel) Representative transient absorption spectral traces for styrene$\subset$Encage inclusion complex in both visible and NIR spectral region obtained at 5 ps pump-probe delay after photoexcitation of the host-guest complex with 400 nm actinic pump pulse.

In order to elucidate the mechanism of light-activated oxygen atom insertion in styrene, we first performed the dark-control where the styrene-Encage complex was allowed to react with the O_2_ in the absence of light. We did not observe any products being formed both at room temperature as well as at an elevated temperature of 60 °C negating the possibility of any ground state reactivity. Photoreaction under Ar-atmosphere did not produce any benzaldehyde suggesting that the source of O-atom in benzaldehyde is aerial O_2_ and that in acetophenone is possibly solvent water. We hypothesized that water addition to the radical cation (shown in Fig. 7 and 9, central panel) should first produce an alcohol-like intermediate in all cages which would undergo cage mediated PCET photo-oxidation to generate the acetophenone. It should be noted that if the cage is TMEDA instead of EnCage we do indeed see 2-phenylethanol due to subtle changes in host-guest packing (see Fig. 8). To test this hypothesis, we carried out photooxidation of 1-phenylethanol inside the cage under ambient and deoxygenated conditions, both of which produced acetophenone (Fig. 9, top right panel). These results indicate that a water attack on sp^2^ carbon first forms a benzylic alcohol intermediate which rapidly undergoes a spontaneous, cascade reaction to generate the carbonyl product via C-H oxidation through proton-coupled electron transfer (PCET) mechanism as reported in previous literature as well^49^.

In order to stabilize and trap the alcohol intermediate and detect it by steady-state measurements, we incarcerated α-methylstyrene inside Encage and performed similar photoreaction. We were able to isolate 2-phenylpropan-2-ol as the stable, tertiary alcohol product in this experiment thereby validating the cascade reactions (Fig. 9, top right panel). We further enriched the solvent systematically with D_2_O and found a linear enrichment of the labeled acetophenone product by performing the photoreaction on styrene ⊂ Encage complex under Ar-atmosphere which further cross-validates the water addition step (Fig. 9, top right panel). It clearly shows that the source of H atom in the keto-methyl group is the solvent water, distinctly different from the classical Wacker reaction pathway. However, under ambient conditions, two dimeric products are formed via intermolecular C-C coupling reaction amongst the incarcerated styrene molecules based on their proper orientation (Fig. 9, middle panel). Under high O_2_ conditions, the radical cation generated at the olefin site is immediately attacked by dissolved O_2_ leading to aldehyde formation, similar to cracking reactions. Based on all these experiments, we further proposed an overall plausible mechanism of the photocatalytic cycle comprising of the light-activation step, dark bi-molecular reaction step either with water, dioxygen or another guest followed by relays of multiple electron and proton transfer steps. Thermal processes that include dioxygen-mediated electron abstraction from the cage and displacement of the product by excess substrate leads to continuity of the photocatalytic cycle (Fig. 9 and Supplementary Fig. 41).

To delve into the reaction, we pumped the host-guest complexes with 400 nm fs laser pulses and probed the intermediates that mediate product formation (excited state dynamics) with another broadband fs probe. As previously reported^48,49,53^, we find that the styrene-Encage complex showed a broad absorption spectrum from 850 nm to 1050 nm (Figs. 7a, 9 (lower panel), attributed to excited state absorption of host-guest coupled CT (Supplementary Figs. 42–44). This feature has a prominent blue shift upto 35–40 ps arising from the solvent relaxation (Fig. 7a, Supplementary Fig. 44). The spectral kinetics at 914 nm fits well with a rise time of 348 ± 21 fs from a LE state along with a bi-exponential decay having a fast component of 6 ± 1 ps (28%) lifetime followed by a slower component of 59 ± 11 ps (72%) lifetime (Supplementary Fig. 43). The solvation correlation function plotted against time additionally extracted the pure solvation timescale decoupled from the population decay and shows a prominent kinetic isotope effect of 1.39 (Supplementary Fig. 45). For measurements in H_2_O, we observe a faster solvation of 0.9 ps followed by a slower component of 11 ps while D_2_O shows 1.6 ps and 14 ps components. We also observed a weak broad absorption feature at 500–700 nm spectral window which we assigned as styrene radical cation absorption based on the literature report (Figs. 7a, 9 (lower panel), Supplementary Fig. 46)^63^. Single point spectral kinetics at 511 nm wavelength showed a rise-time of ~300 fs for the formation of the radical cation followed by a faster decay component of ~5 ps (47% contributing) and a slower decay component of ~ 88 ps (53% contributing) (Supplementary Fig. 43b). Interestingly using similar spectral analysis, we concluded that all styrene derivatives form radical cations inside Encage, although a faster solvation of host-guest coupled CT state in 4-fluorostyrene supports a greater solvent exposure while a slower solvation timescale for 4-methylstyrene supported its buried packing (Fig. 7b, Supplementary Figs. 48 and 58); further indicating towards their corresponding reactivity pattern (Fig. 4c, Supplementary Fig. 31). The greater solvent exposure of 4-fluorostyrene leads to greater solvent stabilization of the associated host-guest CT state which causes blue-most CT absorption at 889 nm for 4-fluorostyrene NIR absorption feature. The least solvent stabilization of CT state was observed for 4-methylstyrene leading to the red-most CT absorption peak maxima at 902 nm for the corresponding NIR absorption feature (Supplementary Fig. 48). The change in the host electronics from Encage to TMEDA and BiPy-cage results in a prominent change in the host-guest coupled CT state lifetime (Fig. 7c, Supplementary Figs. 59–62). For the larger Pd_4_L’_2_^12+^ cavity, CT state population survived upto nanosecond timescale demonstrating the influence of a completely different electronic structure of organic ligand L’ as well as the altered styrene packing (Supplementary Figs. 63–65). Thus, we have established that the styrene radical cation and styrene-host coupled CT states are the driving photo-intermediates for the subsequent water or O_2_-attack products (Supplementary Figs. 66–67).

We believe that the extent of the upfield shifts of styrene protons has a complex dependence on the cage electronics, host-guest packing, cavity hydrophobicity and the exposure of confined guest molecules to interfacial and bulk water. To unravel the nature of guest packing inside three different cavities, 2D proton ROESY experiments were performed with the styrene-cage inclusion complexes (Supplementary Figs. 68 and 70). The off-diagonal, cross-correlation peak intensities give the information of distance-sensitive dipolar coupling amongst protons (Fig. 8). From ROESY intensity analysis of styrene-Encage complex, we found that the olefinic protons are 2.8 Å away from the triazine wall while the aryl meta protons are 4.4–4.6 Å away from the triazine wall (Fig. 8, Supplementary Figs. 68 and 71, Supplementary Table 1). For TMEDA and BiPy cage complexes, similar analysis reveals 5 Å distance between triazine protons and olefin protons (Fig. 8, Supplementary Figs. 69–71, Supplementary Tables 2–3). However, the T1 relaxation timescale of triazine α-protons reduces from 5.4 s to 4.1 s for Encage after styrene incarceration while the T1 remains almost the same for TMEDA cage (2.3 s to 2.1 s) (Supplementary Fig. 72). This also indicates that on average each styrene molecule resides slightly further away from the triazine wall in TMEDA cage than for the Encage. Therefore, based on ROESY-derived distances, we find a structural model that enumerates that intermolecular packing of the olefin part in Encage restricts facile access to the solvent water while the C = C is more exposed to water in TMEDA cavity. Further, relatively higher exposure of the terminal C-site of the olefin part of styrene in TMEDA and BiPy cage and the buried nature of the same C towards the center of the triazine wall in Encage attributed to the subtle change in the relative positioning of the preorganized water and thus led to a remarkable change in the site-selectivity of C-O bond formation. As the olefinic moieties of all styrene molecules are shifted towards the open pores in TMEDA and BiPy cages, the chances of attaining an optimum orientation for intermolecular [2 + 2] dimerization reaction decreased substantially. Therefore, the host-guest modeling has been supported by the corresponding chemistry we observed in all three different cages, unequivocally emphasizing the strength of substrate preorganization in tuning the chemical selectivity.

In order to elucidate the effect of the cationic confinement on the polarization of the olefin site in styrene, we measured Raman spectra of the styrene-cage complex and the nanocage separately. We compared the encapsulated styrene vibrations with that in the free solutions (Fig. 9, top left panel and Supplementary Figs. 73–76). Most significantly, the olefinic sp^2^ C = C stretches are red-shifted due to cage confinement although the phenylic protons show a subtle red-shift inside En cage. We demonstrated a clear red shift for the olefinic C = C stretch from 1634 cm^−1^ to 1597 cm^−1^ for Encage, 1602 cm^−1^ for TMEDA cage and 1621 cm^−1^ for BiPy cage while the phenylic C = C stretches did not show considerable extent of shifts. We predict that selective olefinic C = C bond weakening (13–37 cm^−1^) is originated from a combination of the cage electric field generated by six Pd^2+^ metal sites^49^ and the host-guest charge transfer hybridization. Fundamentally, such electric field-mediated C = C bond weakening and pre-polarization even before light activation circumvents the problem of low polarity at olefin sites. Now, the change in styrene preorganization inside different nanocages is reflected in the differential redshift of C = C stretches i.e., the extent of bond-weakening which dictate the site-selectivity of C-O bond formation and further, the yield of C-C bond formation, additionally probed by 2D ROESY correlation experiments (Supplementary Fig. 76).

Here it is important to draw contrast to previous work of light-mediated photoreactions inside Pd_6_L_4_^12+^ nanocavities via photoinduced electron transfer (PET) process using a high-power UV light source^64,65^ (few Watts). Although demonstration of few PET-based reactions in cavity was striking, building a systematic framework for modular host-guest charge transfer interactions leading to visible-light mediated photochemistry for aromatic substrates has been established only recently^48,49^. Our efforts here with styrene substrates clearly add weightage to the significance of utilizing emergent donor-acceptor CT interactions to drive organic transformations in water using visible light using diffused LED sources^49,66^ (50–175 mW/cm^2^). The generality of our developed concept can be translated to other supramolecular host structures in whose confines modular CT interactions can be sued to carry out bond selective activation chemistry with visible light^67^. Our work therefore affords a simple yet powerful non-covalent activation strategy which utilizes the pre-polarization and the pre-organization of small molecules inside charged cavities via self-assembly.

**Fig. 10:**
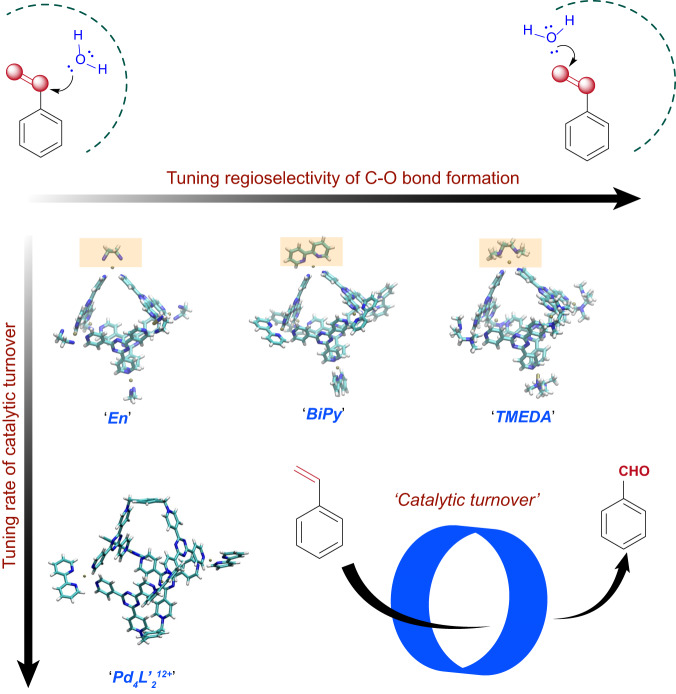
Engineering the shape, pore-size and electronic structure of photocatalyst to tune chemoselectivity and catalytic turn-over frequency. Remote ligand alteration (from En to TMEDA and BiPy, capping ligands of six Pd sites) subtly alters the geometry and volume of supramolecular nanocage which in turn leads to a modulation in host-guest packing and consequences to an altered product selectivity. Additionally, the modified organic ligand, ligand-to-metal ratio and most importantly the larger pore size and volume of Pd_4_L’_2_^12+^ nanocage changes the rate of catalytic turn-over possibly by reducing the substrate entrance and product exit timescale.

In conculsion, we report a photochemical equivalent of the classical ozonolysis reaction and the Wacker oxidation of styrene and its derivatives (Fig. 10). The photoreaction relies on the green reagents, like visible light, water, and dioxygen. We establish our Pd_6_L_4_^12+^ host as an efficient artificial photo-oxidase for photocatalytic conversion of styrene to benzaldehyde. Subsequently, we evolve the nanocage volume and consequent guest preorganization via subtle engineering of the ancillary Pd-capping amine ligands to photo-switch the regioselectivity of C-O bond formation from Markovnikov to anti-Markovnikov water addition on the olefinic site. Utilizing the tunability of host-guest preorganization inside nano-confinement, we exclusively obtain an anti-Markovnikov adduct, primary alcohol from styrene in inert condition which is a rare example of light activated anti-Markovnikov water addition reaction. We further evolve the nanocage to a larger cavity with different electronics and styrene-packing, thereby increasing the rate of catalytic turnover for styrene photo-oxidation. A systematic combination of Raman spectroscopy, transient absorption spectroscopy, NMR correlation experiments, and chemical probe experiments was employed to gather molecular insights on the reaction mechanism. Our paradigm enables us to diversify multiple chemical transformations using a marriage between enzyme-like confinement effects for selective bond-polarization as well as tuning chemoselectivity and host-guest photoredox paradigm for ultrafast bond activation. We further envision heterogeneous photo-electrocatalysis by attaching hosts to a solid electrode surface, and thus move towards a recyclable and industrially scalable method for selective C-O and C-C coupling reactions.

## Methods

### Materials

Styrene (>99%) and all the para-substituted styrene derivatives (4-methylstyrene, 4-fluorostyrene), α-methylstyrene, Palladium (II) chloride (99%) and α,α’-dibromo-p-xylene were purchased from Sigma-Aldrich. 2,4,6-tri(4-pyridyl)−1,3,5-triazine was purchased from TCI Chemicals Pvt. Ltd. Solvents (dichloromethane, chloroform, dimethylformamide, methanol, acetone, n-hexane, ethyl acetate, diethyl ether) were purchased from SD Fine Chemicals. Analytical reagent (AR) grade solvents were used for chemical reactions and HPLC grade solvents were used for spectroscopic measurements. Other chemical reagents like hydrochloric acid, sodium bicarbonate etc. were purchased from Sigma-Aldrich. Heavy water (D_2_O; 99.9 atom %D) was procured from Sigma-Aldrich and used as supplied for the chemical reactions as well as for the NMR measurements.

### Synthesis of Pd_6_L_4_^12+^ nanocage

To the brown suspension of palladium(II) chloride (1.0 g, 5.64 mmol) in water (5 mL), concentrated hydrochloric acid (1 mL) was added. Two-thirds of a solution of ethylenediamine (En, 1.32 mL, 19.85 mmol) in water (3 mL) was added dropwise, yielding a pink precipitate. After warming to 60 °C and the addition of the remaining ethylenediamine solution, the solid dissolved. The mixture was filtrated and adjusted to pH 2.0 with half-diluted hydrochloric acid. The reaction tube was stored at 0 °C for 2 h, and a golden yellow crystalline precipitate of [Pd(En)Cl_2_] formed. The solid was filtrated and dried. The remaining solution was again adjusted to pH 2.0. More product precipitated in the course of 2 h. The combined yellow crystals were dried in vacuum (1.1 g, 96% yield). The exactly similar procedure is followed for N,N,N’,N’-Tetramethylethylenediamine (TMEDA) and 2,2’-Bipyridine (BiPy) complex preparation. In place of Ethylenediamine (En) ligand, TMEDA (1.5 ml, 20 mmol) as neat liquid reagent or BiPy (3.13 mg, 20 mmol) solution in water (15 ml water) was added in the first step. For BiPy complex preparation, cooling of the overall solution at 0 °C was not required.

[Pd(En)Cl_2_] (1.1 g, 4.634 mmol) was suspended in 60 mL water at room temperature. AgNO_3_ (1.567 g, 9.268 mmol) was added to the suspension and the mixture was stirred for 24 hr followed by heating at 60 °C for 2 h. A white solid of AgCl formed and was filtered off. The filtrate was evaporated in a rotary evaporator and the yellow solid was obtained (1.02 g, 87.4% yield). Similarly, [Pd(TMEDA)Cl_2_] and [Pd(BiPy)Cl_2_] were taken to prepare the corresponding nitro complexes with 82% and 93.7% yield, respectively.

2,4,6-tri(4-pyridyl)−1,3,5-triazine (L, 0.65 g, 2.09 mmol) was added to an aqueous solution (80 mL H_2_O) of Pd(X)(ONO_2_)_2_ [X = En, TMEDA, BiPy] (1.2 g, 3.13 mmol) in a 3:2 stoichiometric ratio. The obtained suspension was stirred at room temperature (RT) for overnight and subsequently heated to 80 °C, stirred for another 2 h. A trace amount of insoluble material was filtered out, and the clear solution was rotary evaporated to give 1.66 g (90%) of En-cage as pale-yellow crystals while the % yield of TMEDA and BiPy cages were 94% and 83%, respectively.

### Synthesis of Pd_4_L’_2_^12+^ nanocage

2,4,6-Tris(4-pyridyl)−1,3,5-triazine (TPT, 417 mg, 1.34 mmol, 3.5 equivalent) dissolved in DMF solution was treated with 2 ml DMF solution of α,α′-Dibromo-p-xylene (100 mg, 0.378 mmol, 1 equivalent) in Ar atmosphere and heated at 120 °C for 24 h. A green precipitate was obtained which was then filtered, washed with excess DMF. Then the filter cake was dissolved in hot water (70 °C) to remove excess TPT and then saturated NaBF_4_ solution was added to it to obtain an off-white precipitate of the organic ligand L’ (% Yield = 45%). Separately synthesized [Pd(BiPy)(ONO_2_)_2_] (102.8 mg, 0.266 mmol, 2 equivalent) was added to the suspension of ligand L’ (20 mg, 0.0199 mmol, 1 equivalent). The suspension was heated overnight at 60 ^0^C and yellow colored Pd_4_L’_2_^12+^.4BF_4_^-^.8NO_3_^-^ nanocage (120 mg, 0.036 mmol, % yield = 94 %) was obtained.

### Protocol for guest incarceration inside nanocages

To a 1 ml of 2.5 mM concentration of aqueous cage solution for all the hosts, 10 molar equivalents of styrene or styrene-derivatives as guest molecules with respect to cage was added. The solution was stirred for 45 min to 2 h depending on the nanocage and styrene derivatives chosen as the host and guest respectively. The host-guest complexes were then filtered by a 40 micron millex syringe filter and the filtrates was used for further characterization and photo-reactions. The incarceration protocols were carefully optimized by looking at the ^1^H-NMR signals for the host-guest inclusion complexes at different time intervals.

### NMR measurements

^1^H NMR spectra were recorded on Bruker-800 (800 MHz) and Varian-600 (600 MHz) spectrometer. 2D COSY and ROESY spectroscopic measurements were carried out in Bruker-800 (800 MHz) spectrometer. 2.5 mM cage solutions and 2.5 mM styrene-cage inclusion complexes were taken to perform all the NMR measurements. The measured volume-integral values of the off-diagonal cross-correlation peaks obtained in the ROESY spectrum were used to assess the relative host-guest proton distances. As a reference, we have taken the cross-peaks in between two spatially close protons of the cages [mainly α proton of the triazine versus the protons in the external capping ligands (For En and TMEDA cage, it’s the -CH_2_ protons of the ligands and for BiPy cage, it’s the aromatic phenylic proton next to the N atom)] for which the distance parameters are known either from the crystal structures or from the optimized geometry.

### Steady-state UV-Vis absorption measurements

All the steady-state absorption measurements were carried out using UV-Vis spectrophotometer (JASCO V670). Steady-state absorption has been used for identifying the charge transfer transitions in the host-guest combined manifold.

### Photoreaction setup

The styrene ⊂ host inclusion complexes were taken in a round-bottomed flask with a magnetic bead in a magnetic stirrer. At room temperature, the solution was kept on stirring with 400 nm light illumination by a blue LED (total power = 3 mW) for 6–24 h depending on whether the reaction is stoichiometric or catalytic. The photoreactions were performed in three conditions, one in the inert condition under Ar atmosphere after thorough deoxygenation via freeze-thaw method with the help of the Schlenk line; one in the ambient O_2_ pressure (0.2 atm) and one in the high O_2_ pressure (1.5 atm) using a O_2_ balloon attached with the R.B. flask via a bent-tube. The advancement of the photo-reactions is usually monitored by TLC and GC-MS (Gas Chromatography—Mass Spectroscopy). After completion of the photoreaction, the photoproducts are extracted by adding an optimum amount (0.5 ml for 1 ml aliquot) of dichloromethane (DCM, CH_2_Cl_2_) in the aqueous solution. The extracted photoproducts were either purified by column chromatography or analyzed by GC-MS and ^1^H-NMR.

### GC-MS characterization

The unreacted substrate as well as the formed products are extracted from the reaction mixture through DCM extraction. The extracts are taken for GCMS characterization in an Agilent GC coupled with a Mass spectrophotometer using a fixed temperature ramp and He as a carrying gas for all the samples for getting an identical retention time for one chemical throughout all the measurements.

### Raman measurement

Raman measurements of the host-guest inclusion complexes were performed using an excitation wavelength of 532 nm originated from a solid-state frequency-doubled DPSS Nd:YAG laser (WITec) which was coupled to the Alpha 300 R confocal Raman microscope, WITec GmbH, Ulm (Germany). Styrene was taken neat as a solvent in the cuvette while we took 7 mM aqueous solution of all three cages as well as styrene-cage inclusion complexes. In all the styrene-cage inclusion complexes the styrene was taken as 10 equivalents in comparison to the cages. Due to the poor Raman cross-section of the incarcerated styrene modes we have taken ~2.8 times concentrated host and host-guest solutions for Raman experiments. For En cage, TMEDA cage, and BiPy cage we have taken 20.9 mg (0.007 mmol), 23.3 mg (0.007 mmol), and 24.9 mg (0.007 mmol) of solid cages per 1 ml water solvent respectively to get the 7 mM strength. In the 7 mM cage solutions, 8 µL (0.077 mmol, 10 eq.) of styrene was added and stirred for 5 h in the absence of light at room temperature. The solution was filtered using 45 μm Millex non-sterile syringe-filter to make the final samples. All the Raman measurements were performed using samples prepared using this protocol. We assigned the Raman modes of free guest molecules based on the ab initio CCSD calculations carried out with 6-311 + +g** basis set as well as correlation corrected aug-ccpvdz basis set in gas phase.

### Transient absorption measurements

All pump-probe spectroscopic measurements for capturing the excited state dynamics of different host-guest complexes were carried out using an ultrafast transient absorption spectrometer in the Ultrafast Biophysics lab of the Department of Chemical Sciences at Tata Institute of Fundamental Research, India. A mode-locked Ti-Sapphire laser oscillator (Coherent Micra-5 Mode-locked Titanium: Sapphire Laser system) was used to generate 25 fs pulse with bandwidth of 100 nm and center wavelength of 810 nm, pulse energy 4 nJ/pulse and repetition rate of 80 MHz; further amplified 10^6^ times using a commercially regenerative amplifier. For pump-pulse, we directly took the fundamental 810 nm beam and focused on a 1 mm thick beta barium borate (BBO) crystal which acts as a doubling crystal under a proper phase-matching condition and generates a 400 nm pump pulse. For generating probe-pulse, a portion of the amplified 810-nm output is focused on a 2-mm-thick sapphire crystal to obtain the white-light broadband probe continuum (440 to 1400 nm) which is further directed to the detector. The pump and probe pulses were focused and overlapped spatially as well as temporally within the sample cuvette. The time delay between pump and probe pulses was varied using a Helios software-controlled motorized translation stage fitted with a quadra-pass mirror assembly and thus can generate the difference absorption (ΔA) spectra. All the measurements were performed in flow cuvettes of 2 mm path length to minimize the photodegradation of the samples using a peristaltic pump. Before and after the transient measurements, absorption and ^1^H NMR spectra were recorded on the samples to check for any damage that happened to the samples. Surface Xplorer and Glotaran software were used to perform global analysis of the data for spectral deconvolution.

### Computational methods

The initial coordinates of the Pd_6_L_4_^12+^ cationic cage (TMEDA) as well as the Pd_4_L’_2_^12+^ nanocage were obtained from the Cambridge Crystallographic Data Center (CCDC number 277006 and 276005 respectively). We retained one of the two Pd_6_L_4_^12+^ (L = μ3-2,4,6-tris(4-Pyridyl)−1,3,5-triazine; Pd = (N,N,N’,N’-tetramethyl ethylenediamine) palladium) units from the crystal structure and deleted the other small molecules (e.g., acenaphthylene, syn- 6b,6c,12b,12c-tetrahydrocyclobuta(1,2-a:3,4-a’) diacenaphthylene, nitrate and water molecules). Further, to get the En cage coordinate, we also removed the methyl groups from the (N,N,N’,N’-tetramethyl ethylenediamine) Palladium and replaced Hydrogen atoms in place of methyl groups. Similarly, to get the BiPy cage coordinate, we added bipyridine ligand in place of En ligand. The structure of the resultant Pd_6_L_4_^12+^ (Pd = Ethylenediamine-palladium) nanocages [En, TMEDA and BiPy cages] were all optimized using density functional theory (DFT) at a B3LYP/Lanl2dz/6-31 G* level of theory in the presence of water dielectric. Similarly, with the same level of theory, Pd_4_L’_2_^12+^ cage structure is also optimized. Using the Argus Lab software, a single styrene molecule was first placed close to one of the four triazine moieties of the optimized cationic host structures judiciously with the distance constraints obtained experimentally by 2D ^1^H-^1^H ROESY experiments. The obtained host-guest geometries were subsequently subjected to time-dependent DFT-based excited state calculations at a CAM-B3LYP/Lanl2dz/6-31 G* level of theory. We obtained all the simulated absorption spectra of the different styrene-host complexes. In addition to that, we characterize the red-most styrene to cage charge transfer transitions and associated majorly contributing molecular orbitals. The difference density plot further allowed us to find the exact distance parameter between the barycenter of electron enrichment region and electron depletion region which is termed as D_CT_. This parameter enables us to characterize the red-most guest-to-host CT transitions.

### Supplementary information

Supplementary Information

## Data Availability

All data in the manuscript are available from the corresponding author upon request.
