# Supplementary material for: Tuning light-driven oxidation of styrene inside water-soluble nanocages
Source: Nat Commun. 2024 Feb 28;15:1810. doi: 10.1038/s41467-024-45991-9 (PMC10902312; doi:10.1038/s41467-024-45991-9)
Supplement: Supplementary file 1 — Supplementary Information [file 41467_2024_45991_MOESM1_ESM.docx]

Supplementary Information for

**Tuning light-driven oxidation of styrene inside water-soluble nanocages**

Souvik Ghosal^1^, Ankita Das^1^, Debojyoti Roy^1^ and Jyotishman Dasgupta^1*^

^1^Department of Chemical Sciences, Tata Institute of Fundamental Research, 1 Homi Bhabha Road, Mumbai 400005, India.

Materials and Correspondence should be addressed to J.D.; ^*^Email: [dasgupta@tifr.res.in](mailto:dasgupta@tifr.res.in)

**Supplementary Methods**

**Materials**

Styrene (>99%) and all the *para*-substituted styrene derivatives (4-methylstyrene, 4-fluorostyrene), α-methylstyrene, Palladium (II) chloride (99%) and α,α’-dibromo-p-xylene were purchased from Sigma-Aldrich. 2,4,6-tri(4-pyridyl)-1,3,5-triazine was purchased from TCI Chemicals Pvt. Ltd. Solvents (dichloromethane, chloroform, dimethylformamide, methanol, acetone, n-hexane, ethyl acetate, diethyl ether) were purchased from SD Fine Chemicals. Analytical reagent (AR) grade solvents were used for chemical reactions and HPLC grade solvents were used for spectroscopic measurements. Other chemical reagents like hydrochloric acid, sodium bicarbonate etc. were purchased from Sigma-Aldrich. Heavy water (D_2_O; 99.9 atom %D) was procured from Sigma-Aldrich and used as supplied for the chemical reactions as well as for the NMR measurements.

**Pd_6_L_4_^12+^ Cationic Nanocage Synthesis**

**Pd(X)Cl_2_ [X = En, TMEDA, BiPy] complex synthesis**

To the brown suspension of palladium(II) chloride (1.0 g, 5.64 mmol) in water (5 mL), concentrated hydrochloric acid (1 mL) was added. Two thirds of a solution of ethylenediamine (1.32 mL, 19.85 mmol) in water (3 mL) was added dropwise, yielding a pink precipitate. After warming to 60 ^o^C and the addition of the remaining ethylenediamine solution, the solid dissolved. The mixture was filtrated and adjusted to pH 2.0 with half-diluted hydrochloric acid. The reaction tube was stored at 0^o^C for 2 hours, and a golden yellow crystalline precipitate of [Pd(en)Cl_2_] formed. The solid was filtrated and dried. The remaining solution was again adjusted to pH 2.0. More product precipitated in the course of 2 hours. The combined yellow crystals were dried in vacuum (1.1 g, 96% yield). The exactly similar procedure is followed for N,N,N’,N’-Tetramethylethylenediamine (TMEDA) and 2,2’-Bipyridine (BiPy) complex preparation. In place of Ethylenediamine (En) ligand, TMEDA (1.5 ml, 20 mmol) as neat liquid reagent or BiPy (3.13 mg, 20 mmol) solution in water (15 ml water) was added in the first step. For BiPy complex preparation, cooling of the overall solution at 0 ^0^C was not required.

**Pd(X)(ONO_2_)_2_ [X = En, TMEDA, BiPy] complex synthesis**

[Pd(en)Cl_2_] (1.1 g, 4.634 mmol) was suspended in 60 mL water at room temperature. AgNO_3_ (1.567 g, 9.268 mmol) was added to the suspension and the mixture was stirred for 24 hr followed by heating at 60 ^o^C for 2 hr. A white solid of AgCl formed and was filtered off. The filtrate was evaporated in rotary evaporator and the yellow solid was obtained (1.02g, 87.4% yield). Similarly, [Pd(TMEDA)Cl_2_] and [Pd(BiPy)Cl_2_] were taken to prepare the corresponding nitro complexes with 82% and 93.7% yield respectively.


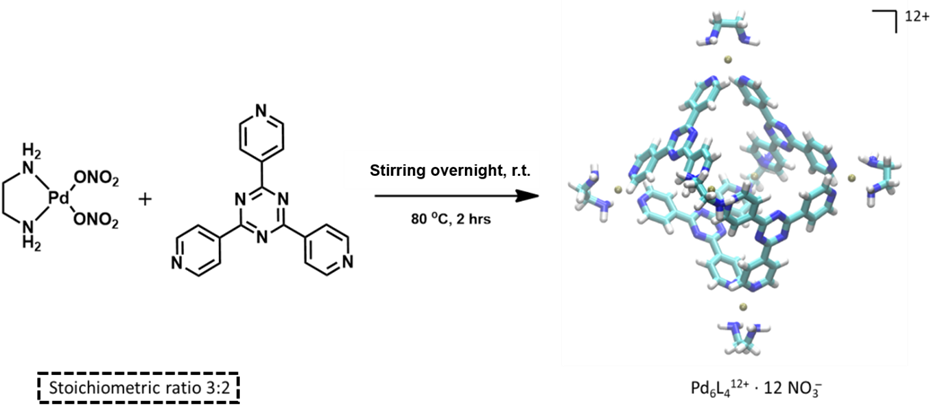


**Supplementary Figure 1.** Synthesis of (En)_6_Pd_6_L_4_^12+^ ∙ 12 NO_3_^-^ nanocage from two constituents: organic ligand 2, 4, 6-trypyridyl-1, 3, 5-triazine (L) and (En)Pd(ONO_2_)_2_ metal complex taken exactly at 2:3 stoichiometric ratio.

**Pd_6_L_4_^12+^ nanocage synthesis**

2,4,6-tri(4-pyridyl)-1,3,5-triazine (0.65 g, 2.09 mmol) was added to an aqueous solution (80 mL H_2_O) of Pd(En)(ONO_2_)_2_ [En = ethylenediamine] (1.2 g, 3.13 mmol) in a 3:2 stoichiometric ratio as per procedure reported ^1^ and protocol was slightly modified by us (as shown in Supplementary Fig 1).^2–4^ The obtained suspension was stirred at room temperature (RT) for overnight and subsequently heated to 80 °C, stirred for another 2 h. A trace amount of insoluble material was ﬁltered out, and the clear solution was rotary evaporated to give 1.66 g (90%) of the cage as pale-yellow crystals.

Physical data of En cage: ^1^H NMR (800 MHz, D_2_O, 27 ^0^C, water suppression): δ 9.05 (d, 24H, Hα of triazine), 8.55 (d, 24H, Hβ of triazine), 2.89 (s, 24H, CH_2_ of En).

Physical data of TMEDA cage: ^1^H NMR (800 MHz, D_2_O, 27 ^0^C, water suppression pulse sequence): δ 9.19 (d, 24H, Hα of triazine), 8.66 (d, 24H, Hβ of triazine), 3.06 (s, 24H, CH_2_ of TMEDA), 2.66 (s, 72H, CH_3_ of TMEDA).

Physical data of BiPy cage: ^1^H NMR (800 MHz, D_2_O, 27 ^0^C, water suppression pulse sequence): δ 9.41 (d, 24H, Hα of triazine), 8.85 (d, 24H, Hβ of triazine), 8.44 (d, 12H, H_m_ of BiPy), 8.32 (t, 12H, H_n_ of BiPy), 7.64 (d, 12H, H_p_ of BiPy), 7.54 (t, 12H, H_o_ of BiPy).

**
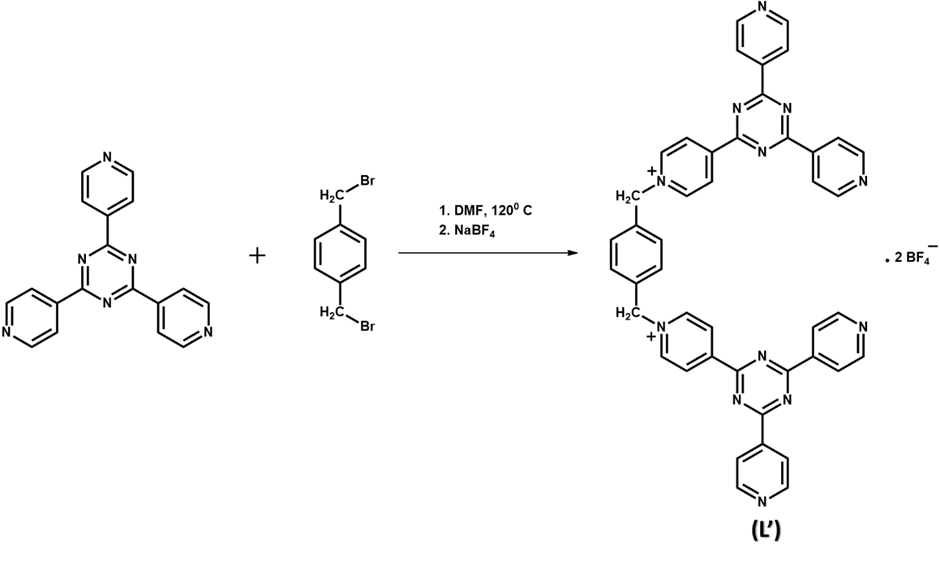
**

**Supplementary Figure 2.** The synthesis of the organic ligand L’ from 2, 4, 6-trypyridyl 1,3,5-triazine and α, α’-dibromoxylene at dimethylformamide (DMF) solvent.

**
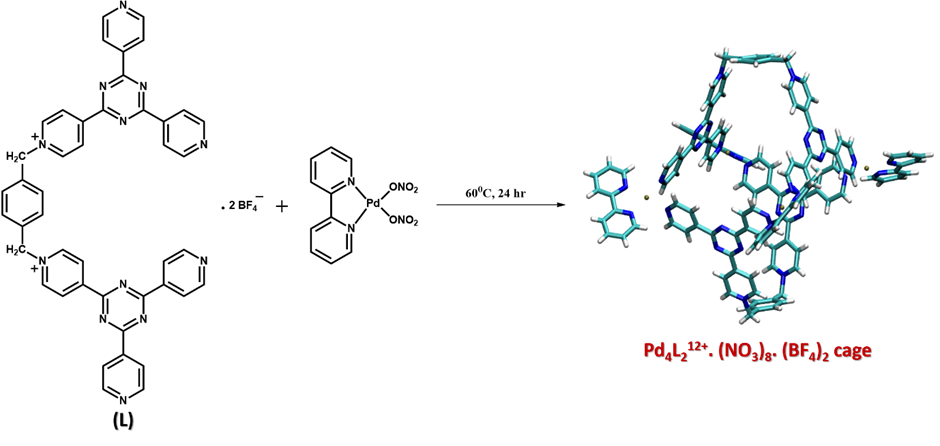
**

**Supplementary Figure 3.** The synthetic scheme for making the new Pd_4_L’_2_^12+^ nanocage from the synthesized organic ligand L’ with the Pd(BiPy)(ONO_2_)_2_ metal complex taken in the 1:2 molar equivalence in water followed by heating and stirring overnight.

**Pd_4_L’_2_^12+^ Nanocage Synthesis**

2,4,6-Tris(4-pyridyl)-1,3,5-triazine (TPT, 417 mg, 1.34 mmol, 3.5 equivalent) dissolved in DMF solution was treated with 2 ml DMF solution of α,α′-Dibromo-p-xylene (100 mg, 0.378 mmol, 1 equivalent) in Ar atmosphere and heated at 120^0^C for 24 hours. A green precipitate was obtained which was then filtered, washed with excess DMF. Then the filter cake was dissolved in hot water (70^0^C) to remove excess TPT and then saturated NaBF_4_ solution was added to it to obtain an off-white precipitate of the organic ligand L’ (% Yield = 45%). Separately synthesized [Pd(BiPy)(ONO_2_)_2_] (102.8 mg, 0.266 mmol, 2 equivalent) was added to the suspension of ligand L’ (20 mg, 0.0199 mmol, 1 equivalent). The suspension was heated overnight at 60 ^0^C and yellow-colored Pd_4_L’_2_^12+^.4BF_4_^-^.8NO_3_^-^ nanocage (120 mg, 0.036 mmol, % yield = 94 %) was obtained. The ^1^H-NMR characterization of the cage was carried out to ascertain its purity (Supplementary Fig 39).

Physical data of triazine based ligand L’: 1,1'-(1,4-phenylenebis(methylene))bis(4-(4,6-di(pyridin-4-yl)-1,3,5-triazin-2-yl)pyridin-1-ium) fluoroborate: ^1^H NMR (800 MHz, DMSO-*d*6, 298K): δ 9.44 (d, J=6 Hz, 4H), 9.33 (d, J=6 Hz, 4H), 8.97 (d, J=4.4 Hz, 8H), 8.69 (d, J=4.8 Hz, 8H), 7.68 (s, 4H), 6.03 (s, 4H) (Supplementary Fig 38).

Physical data of Pd_4_L’_2_^12+^. 8 NO_3_^-^. 4 BF_4_^-^ nanocage: ^1^H NMR (800 MHz, D_2_O, 298K): δ 9.46 (d, *J* = 5.8 Hz, 8H), 9.23 (m, 16H), 9.1 (d, *J* = 6.0 Hz, 8H), 8.88 (d, *J* = 5.6 Hz, 8H), 8.75 (d, *J* = 5.4 Hz, 8H), 8.36 (d, *J* = 8.2 Hz, 8H), 8.32 (t, *J* = 7.8 Hz, 8H), 7.61 (s, 8H), 7.45 (t, J=6.4 Hz, 8H), 7.36 (d, *J* = 5.5 Hz, 8H), 5.86 (s, 8H) (Supplementary Fig 39).

**Incarceration Protocol of Styrene and its Derivatives Inside Nanocages**

We have taken 2.5 mM concentration of aqueous cage solution for all the hosts. Thereafter we added 2.87 µL of liquid styrene (10 equivalents in comparison to the cage equivalence) as guest molecules to 1 ml of the cage solutions. We stirred the solution for 45 minutes to 2 hours depending on the nanocage and styrene derivatives chosen as the host and guest respectively. The host-guest complexes were then filtered by a 40 micron millex syringe filter and the filtrates was used for further characterization and photo-reactions (Supplementary Fig 5). The incarceration protocols were carefully optimized by looking at the ^1^H-NMR signals for the host-guest inclusion complexes at different time intervals.

Styrene (0.0276 mmol, 10 equivalents with respect to the cage molar equivalence) was suspended in a H_2_O/D_2_O solution (1 mL) of 0.0025 mmol of En cage (7.48 mg/ml), TMEDA cage (8.32 mg/ml) and BiPy cage(8.92 mg/ml) respectively to make the three styrene-cage solutions. ^1^H NMR analysis of the clean host-guest complexes revealed the quantitative formation of styrene-cage complexes and also hinted that each molecular cage contained four to five molecules of styrene on an average. For 4-Fluorostyrene, 2.98 µL of guest (10 eq. with respect to the Encage taken) was taken with 2.5 mM En cage solution (1 ml) and kept on stirring for 2 h for complete successful incarceration. For 4-Methylstyrene, similarly 3.3 µL of guest (10 eq. with respect to the En cage taken) was mixed with 2.5 mM of 1 ml En cage solution and stirred it for 1 h for complete successful incarceration. ^1^H NMR analysis of the clean solution for 4-fluorostyrene:Encage complex revealed the quantitative formation of the complex and also hinted that each molecular cage contained four molecules of guests on an average. Similarly, for 4-methylstyrene, NMR analysis predicts the 1:4 host:guest binding stoichiometry for Encage.

Physical data of **styrene ⸦ Encage** complex: ^1^H NMR (800 MHz, D_2_O, 27 ^0^C, water suppression pulse sequence): δ 9.06 (d, 24H, Hα of triazine), 8.49 (d, 24H, Hβ of triazine), 2.85 (s, 24H, CH_2_), 5.65 (t, 8H, meta H_b_ of styrene), 5.5(d, 8H, ortho H_c_ of styrene), 5.15(t, 4H, para H_a_ of styrene), 3.6(d, J = 18.6 Hz, 4H, H_f_ of styrene, trans-coupled with H_d_), 3.25(d, J = 7.2 Hz, 4H, H_e_ of styrene, cis-coupled with H_d_), the H_d_ peak got suppressed for the solvent suppression.

Physical data of **styrene ⸦ TMEDA cage** complex: ^1^H NMR (800 MHz, D_2_O, 27 ^0^C, water suppression pulse sequence): δ 9.24 (d, 24H, Hα of triazine), 8.6 (d, 24H, Hβ of triazine), 3.08 (s, 24H, CH_2_), 2.6 (s, 72H, CH_3_ of TMEDA), 5.55 (t, 10H, meta H_b_ of styrene), 5.3(d, 10H, ortho H_c_ of styrene), 4.82(t, para H_a_ of styrene), 4.3 (d of d, H_d_ of styrene), 3.51(d, J = 21.6 Hz, 5H, H_f_ of styrene, trans-coupled with H_d_), 3.23(d, J = 6.9 Hz, 5H, H_e_ of styrene, cis-coupled with H_d_), the H_a_ and H_d_ peaks got partially suppressed for the solvent peak suppression for which the exact integration could not be measured.

Physical data of **styrene ⸦ BiPy cage** complex: ^1^H NMR (800 MHz, D_2_O, 27 ^0^C, water suppression pulse sequence): δ 9.44 (d, 24H, Hα of triazine), 8.82 (d, 24H, Hβ of triazine), 8.4 (d, 12H, H_m_ of BiPy), 8.29 (t, 12H, H_n_ of BiPy), 7.59 (d, 12H, H_p_ of BiPy), 7.5 (t, 12H, H_o_ of BiPy), 5.78 (t, 10H, meta H_b_ of styrene), 5.55 (d, 10H, ortho H_c_ of styrene), 5.12 (t, 5H, para H_a_ of styrene), 3.74 (d, J = 18.6 Hz, 5H, H_f_ of styrene, trans-coupled with H_d_), 3.4 (d, J = 7.2 Hz, 5H, H_e_ of styrene, cis-coupled with H_d_), the H_d_ peak got suppressed for the solvent suppression.

Physical data of **4-fluorostyrene ⸦ En cage** complex: ^1^H NMR (800 MHz, D_2_O, 300 K): *δ* 9.17 (d, 24H, H*_α_*), 8.54 (d, 24H, H_β_), 2.89 (s, 24 H, En-CH_2_), 5.26-5.3 (broad and complex multiplicity pattern, 16H, aromatic protons), 4.08 (t, 4H, olefinic H_c_), 3.47 (d, J = 19 Hz, 4H, H_d_, trans-coupled with H_c_), 3.23 (d, J = 9.2 Hz, 4H, H_e_, cis-coupled with H_c_).

Physical data of **4-methylstyrene ⸦ En cage** complex: ^1^H NMR (800 MHz, D_2_O, 300 K): δ 9.015 (d, 24H, H*_α_*), 8.385 (d, 24H, H_β_), 2.72 (s, 24 H, En-CH_2_), 4.57-4.61 (broad and complex multiplicity pattern, underneath water peak, aromatic protons and olefinic Hc), 3.78-3.81 (complex multiplicity pattern, broad, 12H, olefinic H_d_ and H_e_), 0.18 (s, 12H, *para*-CH_3_ of styrene molecule).

**NMR Characterizations**

^1^H NMR spectra were recorded on Bruker-800 (800 MHz) and Varian-600 (600 MHz) spectrometer. 2D COSY and ROESY spectroscopic measurements were carried out in Bruker-800 (800 MHz) spectrometer. 2.5 mM cage solutions and 2.5 mM styrene-cage inclusion complexes were taken to perform all the NMR measurements.

In the COSY spectra, the off-diagonal peaks between two protons of the sample denotes that those two protons are coupled through chemical bonding (upto three-bond distance) and helps the spectral assignment. While in the ROESY spectra, the cross-peak intensity is a consequence of spatial dipolar coupling between two spins and hence, inversely proportional to the sixth power of the distance between the two spins. The presence of off-diagonal peaks for two spins in any system can give us relative quantitative idea about the distance parameters in between spins presence in the system. In the manuscript, we have taken the cross-peaks in between two spatially close protons of the cages [mainly α proton of the triazine versus the protons in the external capping ligands (For En and TMEDA cage, it’s the -CH_2_ protons of the ligands and for BiPy cage, it’s the aromatic phenylic proton next to the N atom)] as our reference for which the distance parameters are known either from the crystal structures or from the optimized geometry. Now comparing the volume integral i. e. the overall intensity of the other cross peaks appeared for different host-guest proton pairs with that reference cross-peak volume integral, we can calculate the corresponding distance parameters [using the formula: $\frac{V_{ij}}{V_{mn}}=\frac{r_{mn}^{6}}{r_{ij}^{6}}\ldots\ldots(1)$]. Many such distance constraints enable us making a reasonably good host-guest packing model. This further helps us understand the fundamental preorganization of guest molecules inside nano-confinement which ultimately dictates the selectivity in the photochemistry.

**Steady State Absorption Measurements**

All the steady state absorption measurements were carried out using UV-Vis spectrophotometer (JASCO V670). In the steady state absorption measurement, a continuous broad-band lamp source (here, Deuterium arc lamp for 190-350 nm source, Tungsten filament for 350-2500 nm source) is incident on the sample kept in the cuvette. After passing through the sample the transmitted light is passed through a dual-grating single monochromator (1200 grooves/mm in UV-VIS region and 300 grooves/mm in NIR region). A PMT detector is provided for the UV/VIS region and a Peltier-cooled PbS detector is employed for the NIR region. Steady state absorption has been used for identifying the charge transfer transitions in the host-guest combined manifold.

**GCMS Characterizations**

The unreacted substrate as well as the formed products are extracted from the reaction mixture through DCM extraction. The extracts are taken for GCMS characterization in an Agilent GC coupled with Mass spectrophotometer using a fixed temperature ramp and He as a carrying gas for all the samples for getting an identical retention time for one chemical throughout all the measurements.

**Raman Characterizations**

Raman measurements of the host-guest inclusion complexes were performed using an excitation wavelength of 532 nm originated from a solid-state frequency doubled DPSS Nd:YAG laser (WITec) which was coupled to the Alpha 300R confocal Raman microscope, WITec GmbH, Ulm (Germany). A 100 µm optical fiber was used to collect the back scattered light employing a lens-based ultra-high throughput spectrometer (UHTS300) with 1800 grooves/mm grating coupled to a back illuminated CCD-camera (1024$\times$128 pixels, Peltier-cooled to -65 °C) for detection. The spectral resolution of the spectrograph was ~ 2 cm^-1^. The laser was focused into the solution flowing through a flow-cuvette using a 10X objective. Styrene was taken neat as a solvent in the cuvette while we have taken 7 mM aqueous solution of all the three cages as well as styrene-cage inclusion complexes. In all the styrene-cage inclusion complexes the styrene was taken as 10 equivalents in comparison to the cages. The host-guest inclusion complexes were made in a similar fashion as mentioned in section 1.2.3 in details. Due to the poor Raman cross section of the incarcerated styrene modes we have taken ~2.8 times concentrated host and host-guest solutions for Raman experiments. For En cage, TMEDA cage and BiPy cage we have taken 20.9 mg (0.007 mmol), 23.3 mg (0.007 mmol) and 24.9 mg (0.007mmol) of solid cages per 1 ml water solvent respectively to get the 7 mM strength. In the 7 mM cage solutions, 8 µL (0.077mmol, 10 eq.) of styrene was added and stirred for 5 hours in the absence of light at room temperature. The solution was filtered using 45 μm Millex non-sterile syringe-filter to make the final samples. All the Raman measurements were performed using samples prepared using this protocol. We assigned the Raman modes of free guest molecules based on the *ab initio* CCSD calculations carried out with 6-311++g** basis set as well as correlation corrected aug-ccpvdz basis set in gas phase.


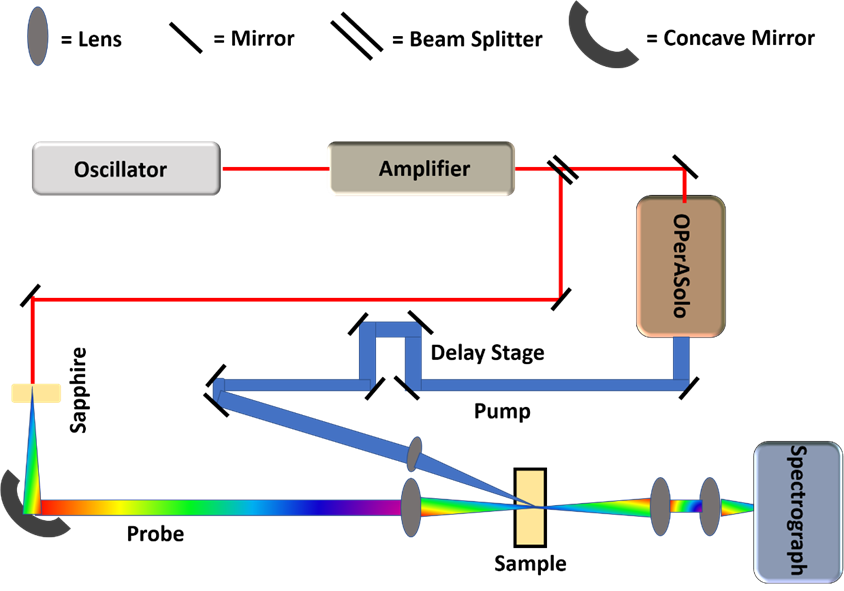


**Supplementary Figure 4.** Schematic representation of fs-TA setup. Femtosecond probe pulse and femtosecond actinic pump pulse are spatially overlapped on the sample and the transmitted probe profile is detected by the spectrograph.

**Transient Absorption Measurements**

All pump-probe spectroscopic measurements for capturing the excited state dynamics of different host-guest complexes had been carried out using an ultrafast transient absorption spectrometer in the Ultrafast Biophysics lab of the department of Chemical Sciences at Tata Institute of Fundamental Research, India.^5^ A mode-locked Ti-Sapphire laser oscillator (Coherent Micra-5 Mode-locked Titanium: Sapphire Laser system) is used to generate 25 fs pulses with bandwidth of 100 nm, pulse energy 4 nJ/pulse and repetition rate of 80 MHz. The ultrashort pulse is generated by self-focusing based Kerr effect mechanism due to the non-linearity introduced by the high power of the LASER field. A prism pair along with an adjustable slit is utilized to tune the center wavelength and bandwidth of output pulses. We usually keep the center wavelength at 810 nm for our measurements. The output beam from femtosecond oscillator is amplified based on chirped pulse amplification (CPA) technique using Coherent Legend Elite amplifier laser system. In brief, the femtosecond seed pulse is first stretched to picosecond using an optical grating in order to reduce its peak power. The amplification happens in a Ti-Sapphire cavity that is pumped by a 22 W of 532 nm nanosecond laser from an Nd:YLF laser with 1 kHz repetition rate. The Ti-Sapphire regenerative amplifier amplifies the pulse energy by a factor of ~10^6^ to obtain ~3.5-4 mJ/30 fs output at 1 kHz repetition rate. The regenerative amplification technique allows the seed pulse to multi-pass in the cavity where the stimulated emission photons are added at each pass resulting in a much higher overall gain. After the amplification, the pulse is recompressed using a grating compressor inside the amplifier. The output < 30 fs fundamental beam having its peak at 800 nm is subsequently split to generate the excitation pump pulse and the low power probe pulse by a beam-splitter. Part of the fundamental beam goes through an optical parametric amplifier (OPA) system (OPerA-Solo from Light Conversion) where it generates tunable ~30 fs actinic pump pulses with tunability from 285-2700 nm. OPerA-Solo is a two-stage parametric amplifier of white-light continuum where the first stage is pre-amplification and second stage is power amplification. It uses the second order nonlinear susceptibility (χ^(2)^) of a medium (β-barium borate crystal, BBO crystal which does not possess any inversion symmetry, hence anisotropic) to amplify selected frequency components of a white light seed beam. The wavelength tunability is achieved by a combination of changing the delay between white light and the pump pulse, changing the angle of nonlinear crystal (to change the phase matching condition in the optical indicatrix description defined in the birefringent crystals used) and using different mixing crystals. For 400 nm pump-pulse, however, we don’t utilize the OPA system due to insufficient power of the OPA generated pump pulse. Rather we directly take the fundamental beam and focused on a 1 mm thick BBO crystal which acts as a doubling crystal under a proper phase-matching condition and generates 400 nm pump pulse. Part of the fundamental pulse is focused onto a 2-mm thick sapphire crystal using a concave mirror. This generates broadband white light continuum via self-phase modulation. The broadband probe beam is then collimated using another concave mirror, then guided and focused to the sample cell. For measurements, a pump beam was attenuated to ∼300−450 nJ per pulse depending on the signal obtained for the host-guest complexes and the photo-stability of the corresponding host-guest complexes. The pump and probe pulses were focused and overlapped spatially as well as temporally within the sample cuvette. The time delay between pump and probe pulses were varied using a Helios software-controlled motorized translation stage fitted with a quadra-pass mirror assembly and thus can generate the difference absorption (ΔA) spectra. All the measurements were performed in flow cuvettes of 2 mm pathlength to minimize the photodegradation of the samples using a peristaltic pump. Before and after the transient measurements, absorption and ^1^H NMR spectra were recorded on the samples to check for any damage happened to the samples.

The instrument response function (IRF) was determined as ∼250 fs for 400 nm pump pulse, from an optical Kerr effect (OKE) experiment on a 1 mm glass. The experimentally measured transient signal decay *F(t)* is the convolution of the instrument response function (IRF), *R(t)* and signal intensity decay function *I(t)* which can be written as:

$F\left( t \right)=R\left( t \right)\otimes I\left( t \right)\ldots\ldots(2A)$

or

$F\left( t \right)=\int_{0}^{t} R\left( t^{'} \right)I\left( t-t^{'} \right)dt^{'}\ldots\ldots(2B)$

Where the signal intensity decay function *I(t)* can be expressed as sum of multiple exponentials such as

$I\left( t \right)=\sum_{t=1}^{n} \left( \alpha_{i} exp(-t/\tau_{i}) \right)\ldots\ldots(3)$

The IRF, *R(t)* can be described by a Gaussian as

$$R\left( t \right)=\frac{\left\{ exp(-log(2)(2\left( t-\mu\right)/{\Delta)}^{2}) \right\}}{\Delta^{'}\sqrt{2\pi}}\ldots\ldots(4)$$

Where *μ* is the mean and *Δ* is the full width half maximum (FWHM) of the IRF given by $\Delta^{'}= \Delta/(2\sqrt{log(2}))$

Time resolved data were analyzed by multi-exponential fitting with the help of IGOR 5 wavemetrics software. The used fitting equation consists of four exponential time constants in convolution with the instrument response function (IRF) which provides the decay time constants along with the associated amplitudes for any single point kinetics.^6–8^

The equation used is given below

$$y\left( t \right)= w_{1}\left[ \left( w_{2}e^{\frac{\left[ k_{0}^{2}-4bk_{0}\left( t-w_{9} \right) \right]}{4b}} \right)\mathrm{normdist}\left( \frac{2b\left( t-w_{9} \right)-k_{0}}{\sqrt{2b}} \right)+\left( w_{4}e^{\frac{\left[ k_{1}^{2}-4bk_{1}\left( t-w_{9} \right) \right]}{4b}} \right)\mathrm{normdist}\left( \frac{2b\left( t-w_{9} \right)-k_{1}}{\sqrt{2b}} \right)+ \left( w_{6}e^{\frac{\left[ k_{2}^{2}-4bk_{2}\left( t-w_{9} \right) \right]}{4b}} \right)\mathrm{normdist}\left( \frac{2b\left( t-w_{9} \right)-k_{2}}{\sqrt{2b}} \right) +\left( \left[ -1-\left( w_{4}-w_{6} \right) \right]e^{\frac{\left[ k_{3}^{2}-4bk_{3}\left( t-w_{9} \right) \right]}{4b}} \right)\mathrm{normdist}\left( \frac{2b\left( t-w_{9} \right)-k_{3}}{\sqrt{2b}} \right) \right]\ldots(5)$$

Where,

$$b=\frac{4\ln2}{w_{0}^{2}}k_{0}=\frac{1}{w_{3}}k_{1}=\frac{1}{w_{5}}k_{2}=\frac{1}{w_{8}}$$

$w_{0}$= IRF determined by the Optical Kerr experiment

$w_{1}$= common scaling factor that scales the curve

$w_{2}, w_{4}, w_{6}$= amplitudes of the first, second, third exponentials

$w_{3}, w_{5}, w_{7}, w_{8}$= Four time constants

$w_{9}$= zero time, the offset of actual pump probe

Global and target analyses (GTA) of the transient absorption data were carried out with the help of the Surface Xplorer software obtained from Ultrafast Systems for preparing the 3-D data matrix (http://www.ultrafastsystems.com/surface-xplorer/) and then Glotaran software (http://glotaran.org/) for further spectral deconvolution. In the Glotaran software, we initially estimated the number of principle components by Singular Value Decomposition (SVD) analysis by adding more and more components. Then we further optimized the principle decay/rise components by the global fitting of the whole 3-D data matrix consisting of time, wavelength and difference absorption intensity. The quality of fitting can be probed by checking the quality of root mean square deviation. For styrene and its derivatives $\subset$ Pd_6_L_4_^12+^ nanocages (En, TMEDA, BiPy cages), we optimally fitted the two species sequential model (A $\to$ B, where A is the first photoexcited state population and B is the population evolved from A and finally going back to the ground state) fit for the visible window data. We fitted the white light dispersion (group velocity dispersion, chirping) by a third order polynomial and kept the gaussian instrument response function (IRF) fixed at 0.2 ps. The global fitting assisted us in getting a spectrally deconvolved Evolution Associated Decay Spectra (EADS) as a signature of different populations existing in the excited state manifolds of the host-guest complexes.


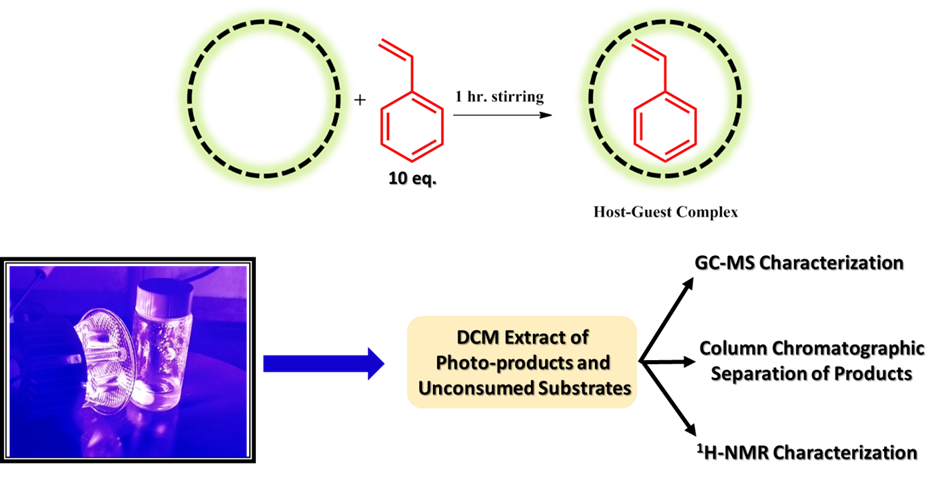


**Supplementary Figure 5.** The set-up for photochemical reaction on host-guest complexes.

**Photoreaction Set-up**

The **styrene ⸦ host** inclusion complexes were taken in a round-bottomed flask with a magnetic bead in a magnetic stirrer. At room temperature, the solution kept on stirring with 400 nm light illumination by a blue LED (total power = 3mW) for 6-24 hours depending on whether the reaction is stoichiometric or catalytic. The photo-reactions were performed in three conditions, one in the inert condition under Ar atmosphere after thorough deoxygenation via freeze-thaw method with the help of the Schlenk line; one in the ambient O_2_ pressure (0.2 atm) and one in the high O_2_ pressure (1.5 atm) using a O_2_ balloon attached with the R.B. flask via a bent-tube. The photocatalytic reactions are performed usually under high oxygen pressure due to faster turn-over and exclusive selectivity for the products. The advancement of the photo-reactions is usually monitored by TLC and GC-MS (Gas Chromatography - Mass Spectroscopy). After completion of the photo-reaction, the photo-products are extracted by adding optimum amount (0.5 ml for 1 ml aliquot) of dichloromethane (DCM, CH_2_Cl_2_) in the aqueous solution (Supplementary Fig 5). The extracted photoproducts were either purified by column chromatography or analyzed by GC-MS and ^1^H-NMR.

**Computational Methods: Electronic Structure Calculations**

The initial coordinates of the Pd_6_L_4_^12+^ cationic cage (TMEDA) as well as the Pd_4_L’_2_^12+^ nanocage were obtained from the Cambridge Crystallographic Data Centre (CCDC number 277006 and 276005 respectively). We retained one of the two Pd_6_L_4_^12+^ (L = μ3-2,4,6-tris(4-Pyridyl)-1,3,5-triazine; Pd = (N,N,N',N'-tetramethyl ethylenediamine) palladium) units from the crystal structure and deleted the other small molecules (e.g. acenaphthylene, syn- 6b,6c,12b,12c-tetrahydrocyclobuta(1,2-a:3,4-a') diacenaphthylene, nitrate and water molecules). Further, to get the En cage coordinate, we also removed the methyl groups from the (N,N,N',N'-tetramethyl ethylenediamine) palladium and replaced hydrogen atoms in place of methyl groups. Similarly, to get the BiPy cage coordinate, we added bipyridine ligand in place of En ligand. The structure of the resultant Pd_6_L_4_^12+^ (Pd = Ethylenediamine-palladium) nanocages [En, TMEDA and BiPy cages] were all optimized using density functional theory (DFT) at a B3LYP/Lanl2dz/6-31G* level of theory in the presence of water dielectric. Similarly, with the same level of theory, Pd_4_L’_2_^12+^ cage structure is also optimized. Now using the Argus Lab software, a single styrene molecule was first placed close to one of the four triazine moieties of the optimized cationic host structures judiciously with the distance constraints obtained experimentally by 2D ^1^H-^1^H ROESY experiments. The obtained host-guest geometries were subsequently subjected to time dependent DFT based excited state calculations at a CAM-B3LYP/Lanl2dz/6-31G* level of theory. The previously used B3LYP functional is also a hybrid functional but CAM-B3LYP is a range separated hybrid functional. The expression of the exchange-correlation functional in CAM-B3LYP holds electron-electron distance dependent optimized mixing of non-local Hartree-Fock exchange and local DFT exchange terms. Thus, use of CAM-B3LYP functional is usually recommended especially when presence of charge transfer transitions is expected.^9^ Now with the excited state calculations, we obtained all the simulated absorption spectra of the different styrene-host complexes. In addition to that, we characterize the red-most styrene to cage charge transfer transitions and associated majorly contributing molecular orbitals. The difference density plot further allowed us to find the exact distance parameter between the barycentre of electron enrichment region and electron depletion region which is termed as D_CT_.^10^ This parameter enables us to characterize the red-most guest to host CT transitions. We extensively used electronic structure calculations to predict the presence of CT transitions in order to validate the host-guest CT paradigm as well as to obtain the influence of different electronic coupling in different host-guest systems in the CT transitions associated with.


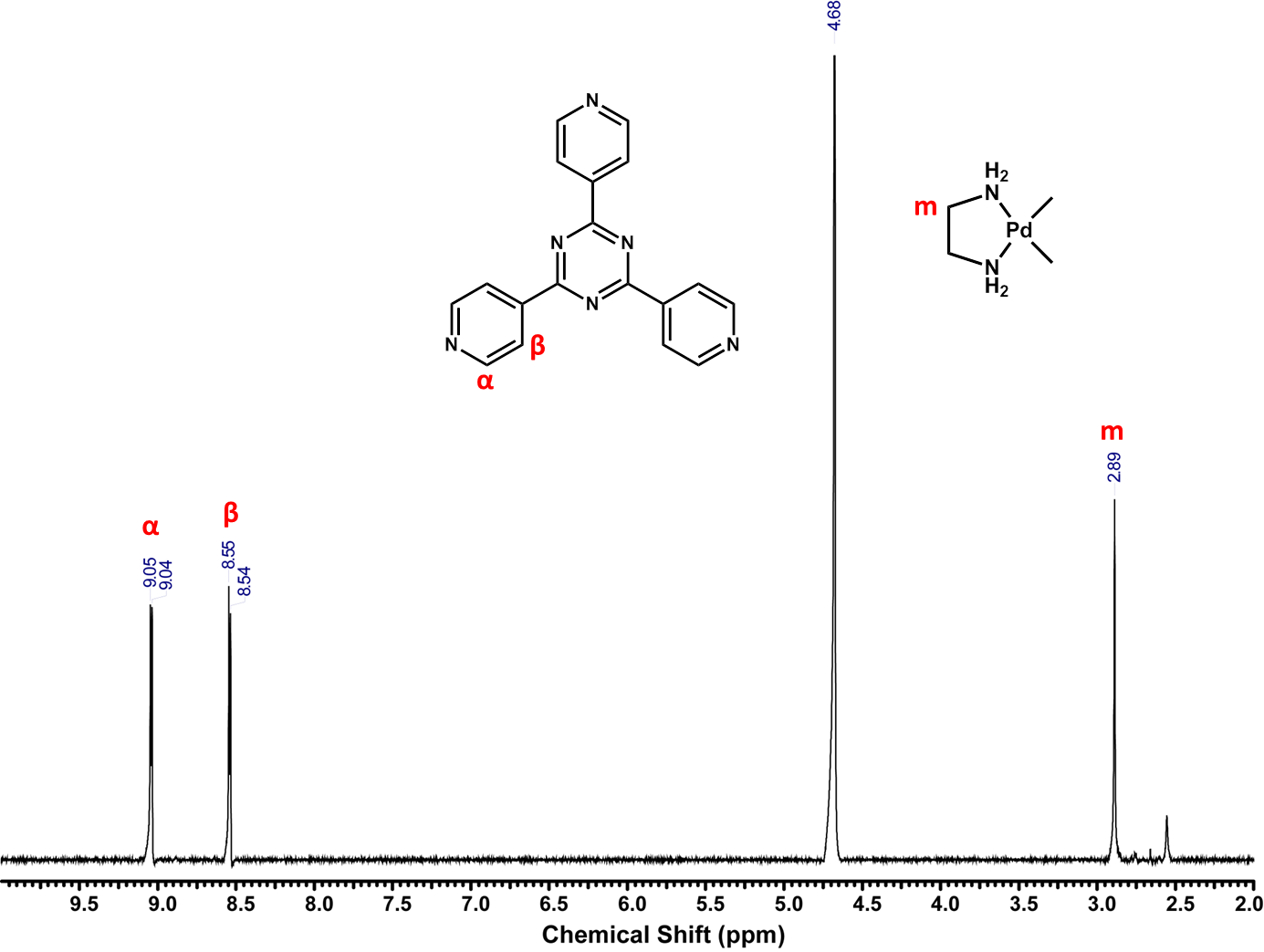


**Supplementary Figure 6.** ^1^H-NMR characterization of En cage (800 MHz, D_2_O, 298K)


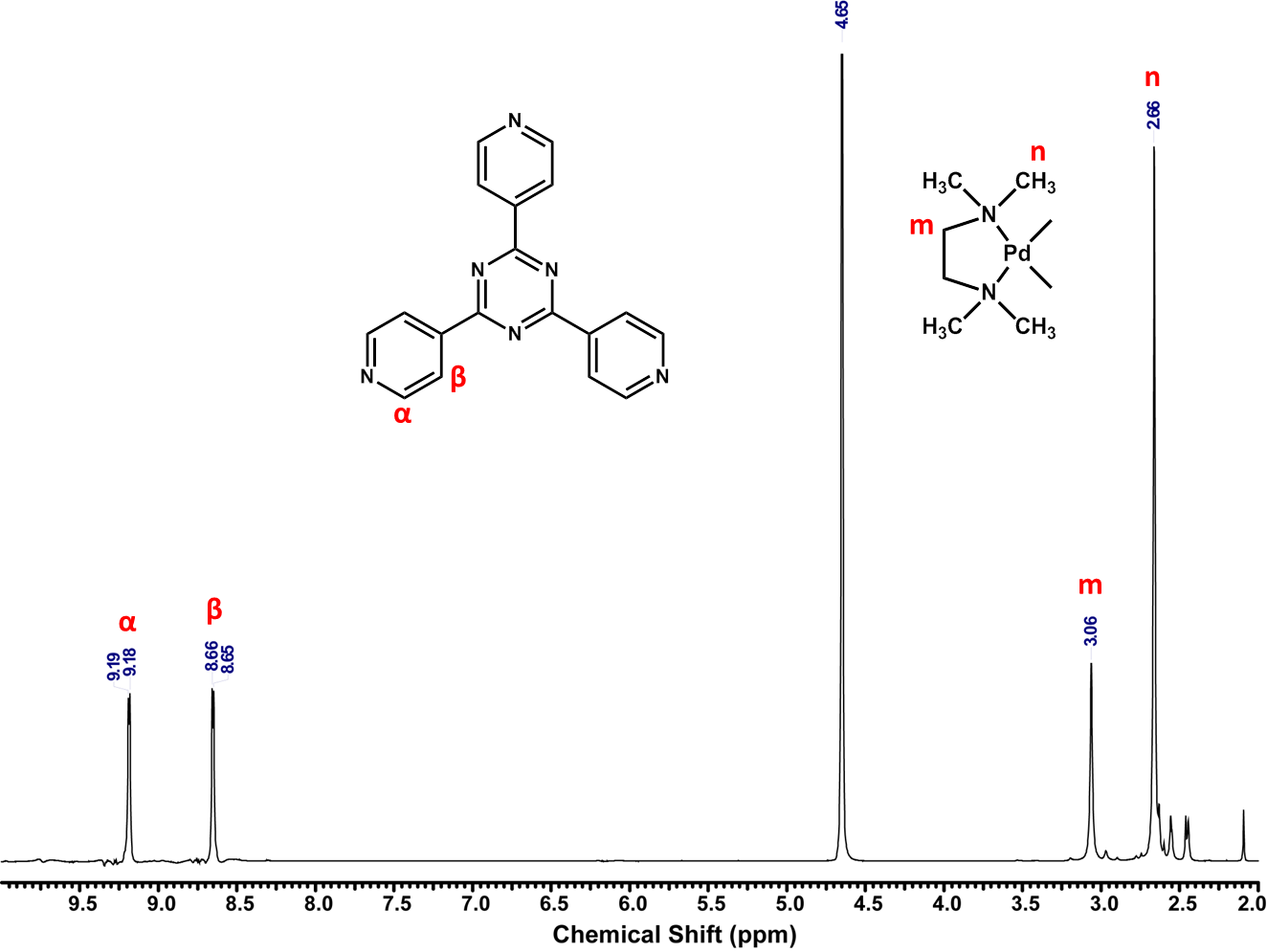


**Supplementary Figure 7.** ^1^H-NMR characterization of TMEDA cage (800 MHz, D_2_O, 298K).


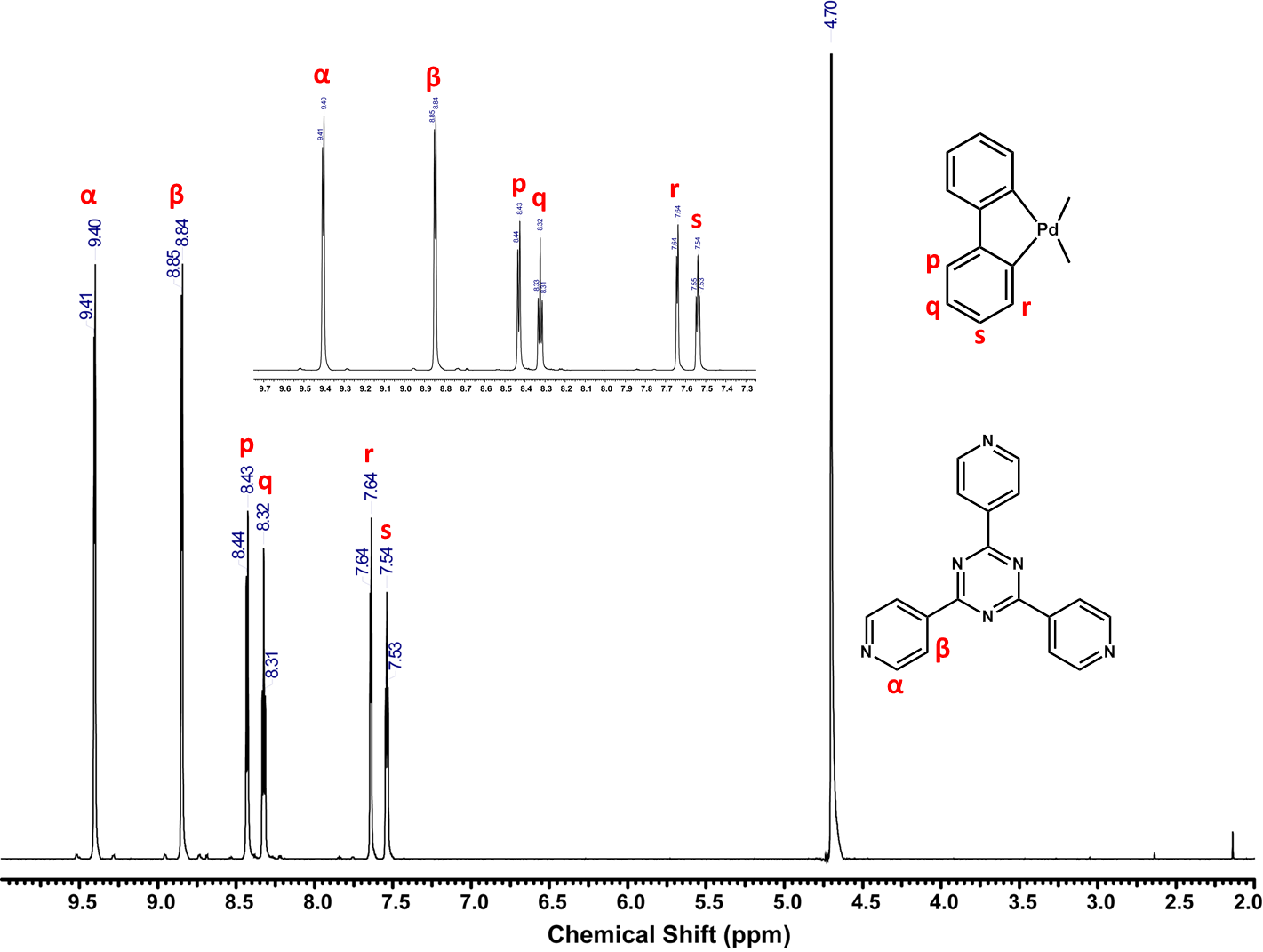


**Supplementary Figure 8.** ^1^H-NMR characterization of BiPy cage (800 MHz, D_2_O, 298K). The expanded aromatic region is shown in the inset.


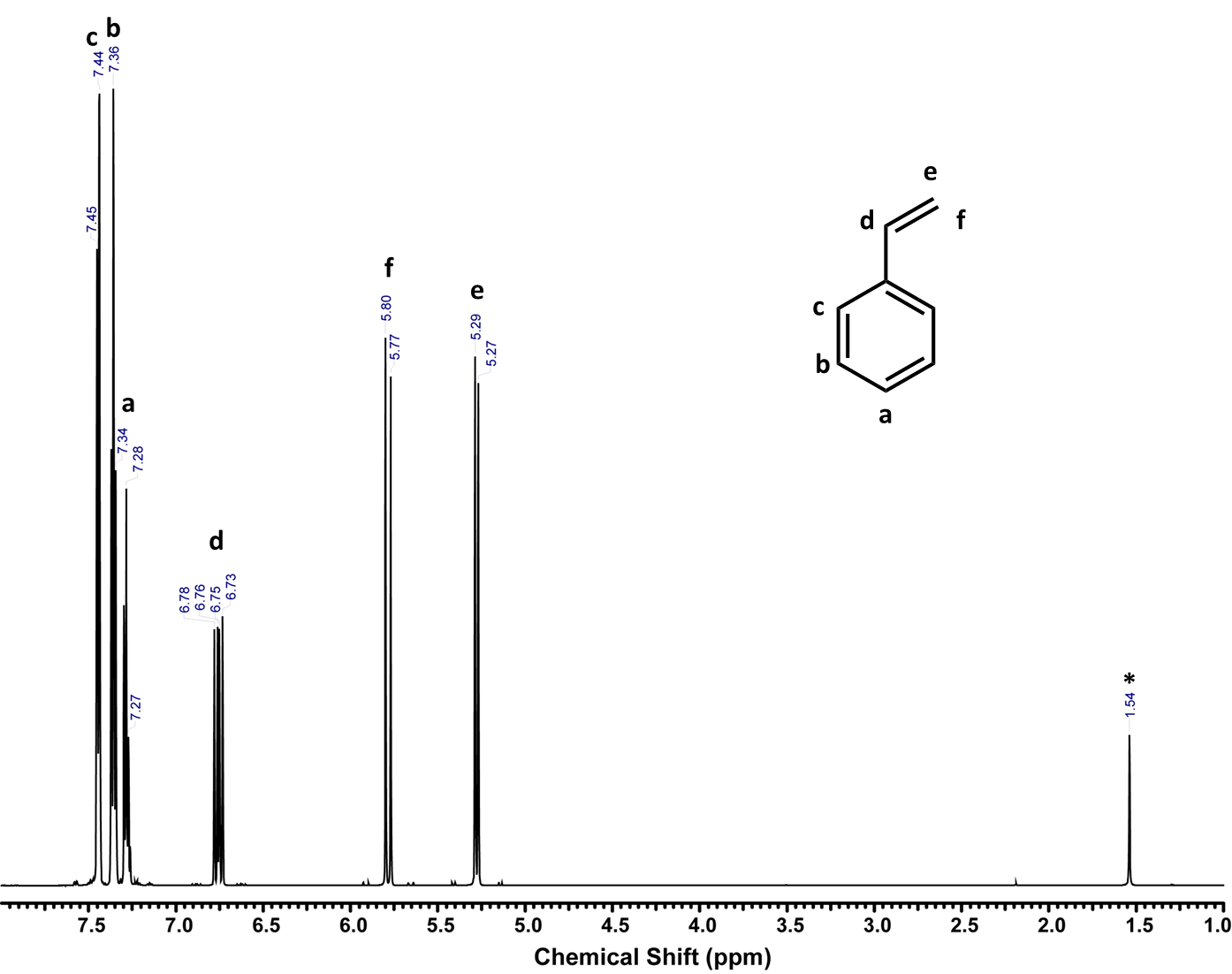


**Supplementary Figure 9.** ^1^H-NMR characterization of free styrene (800 MHz, CD_2_Cl_2_, 298K). The solvent impurities marked by asterisks. The sharp peak linewidth suggests fast rotational tumbling motion of the molecule.

**
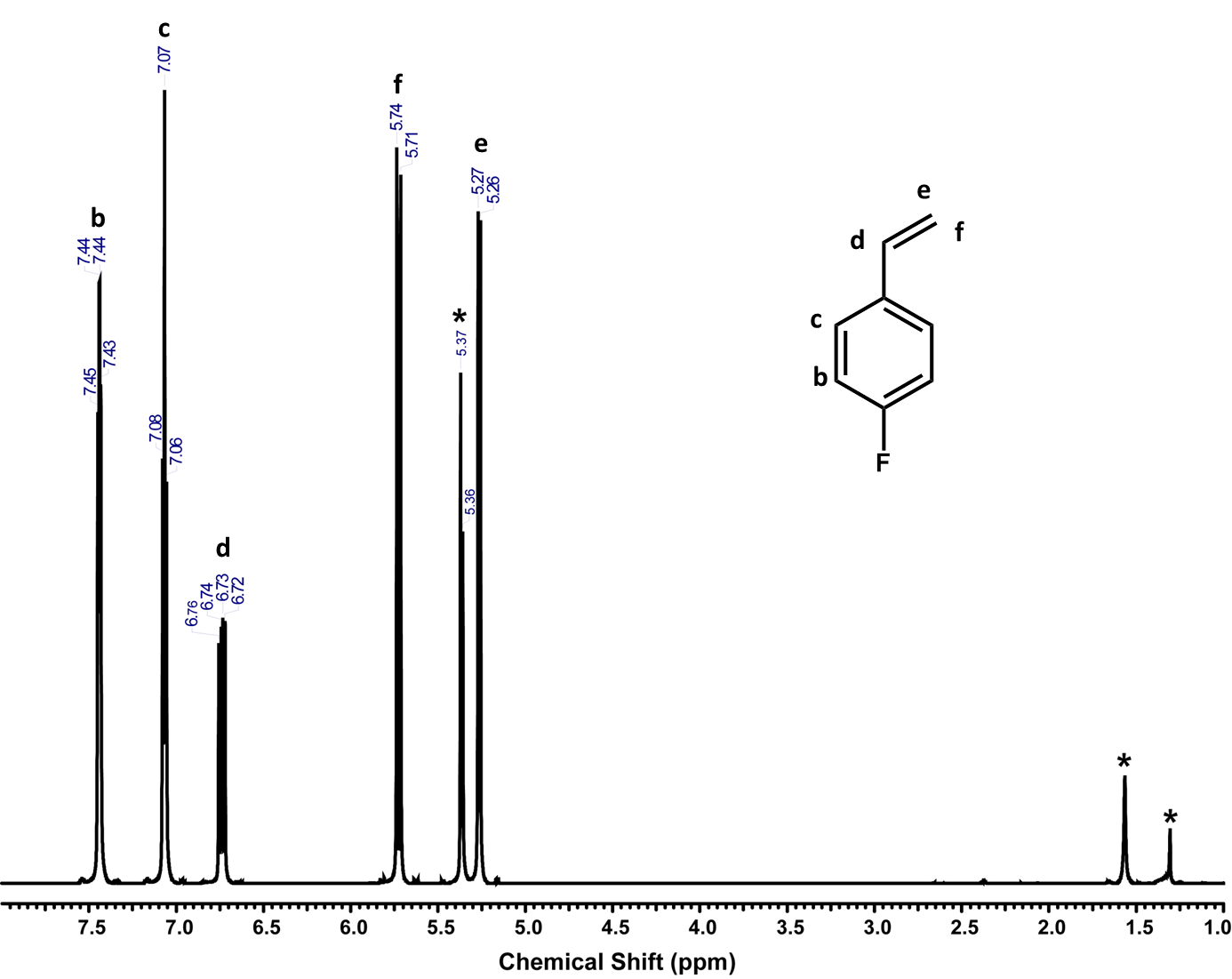
**

**Supplementary Figure 10.** ^1^H-NMR characterization of free 4-fluorostyrene (800 MHz, CD_2_Cl_2_, 298K). The solvent impurities marked by asterisks.


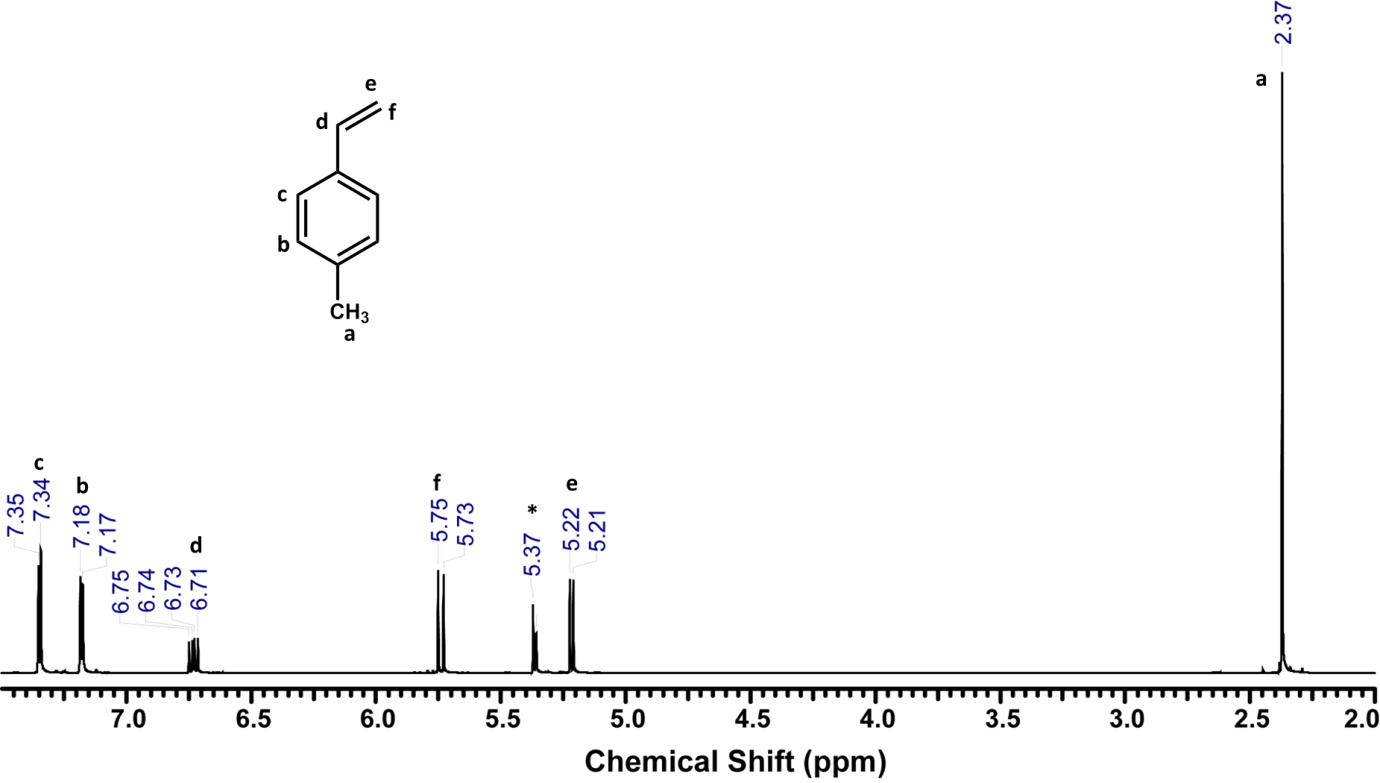


**Supplementary Figure 11.** ^1^H-NMR characterization of free 4-methylstyrene (800 MHz, CD_2_Cl_2_, 298K). The solvent impurities marked by asterisks.


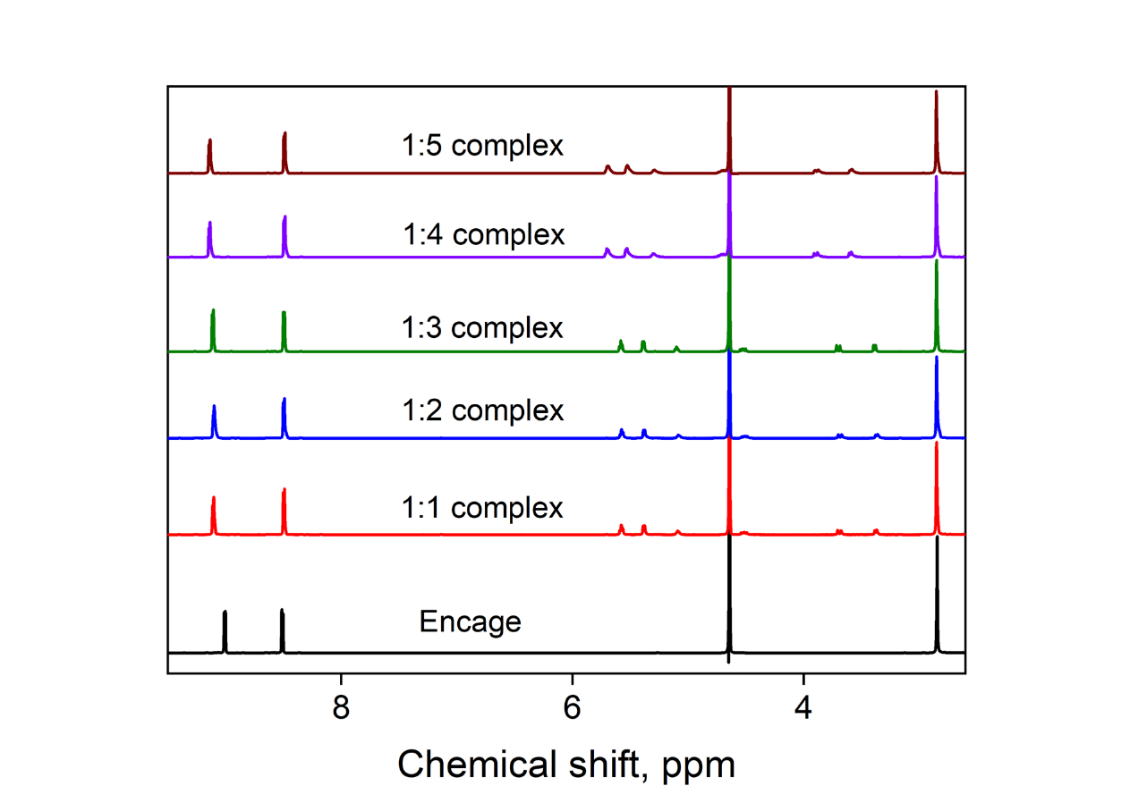


**Supplementary Figure 12.** Comparison of normalized proton-NMR spectra of Encage and **styrene** $\boldsymbol{\subset}$ **Encage** complexes (600 MHz, D_2_O, 298K). formed by incubating different molar equivalents of styrene to the cage solution. The stoichiometric ratios mentioned in the figure denote to the incubation conditions.


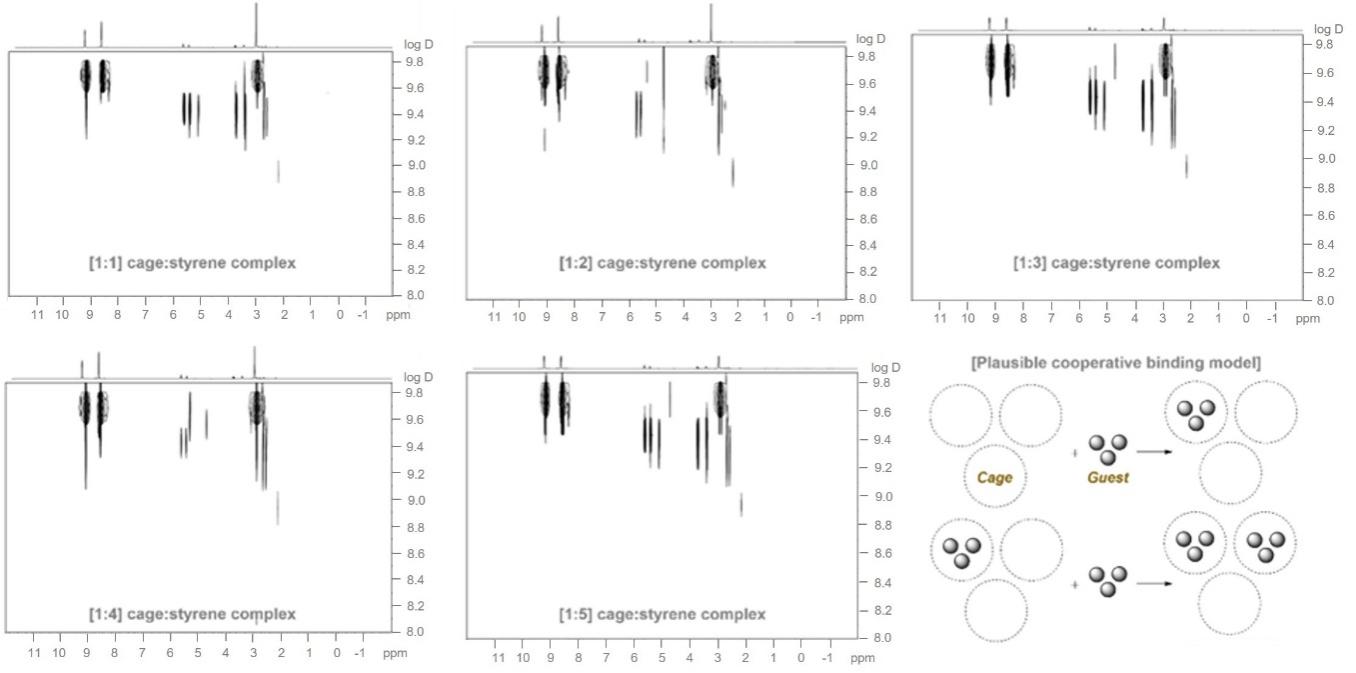


**Supplementary Figure 13.** Comparison of ^1^H-DOSY spectra of **styrene** $\boldsymbol{\subset}$ **Encage** complexes (600 MHz, D_2_O, 298K) formed by incubating different molar equivalents of styrene to the encage solution which indicates towards a plausible cooperative guest binding model.


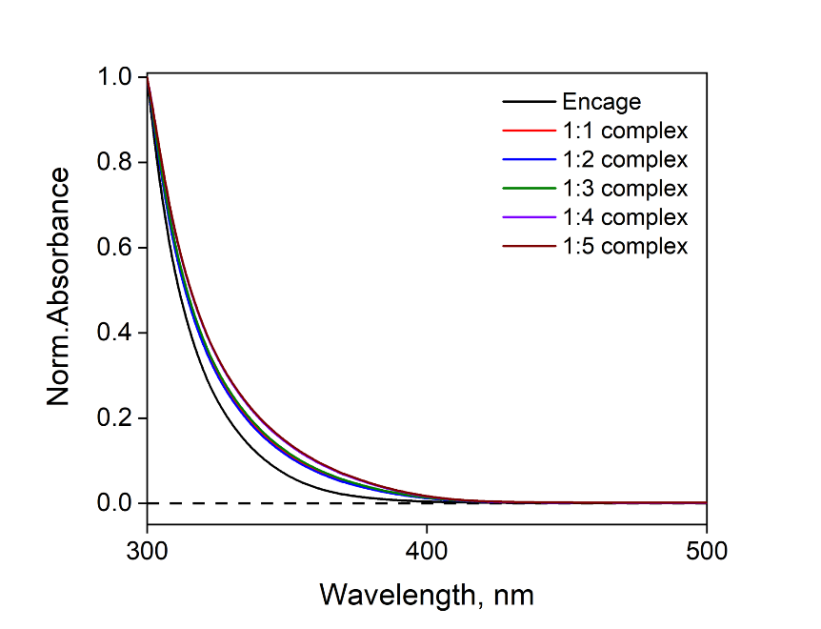


**Supplementary Figure 14.** Comparison of normalized steady-state absorption spectra of Encage and **styrene** $\boldsymbol{\subset}$ **Encage** complexes formed by incubating different molar equivalents of styrene to the cage solution.


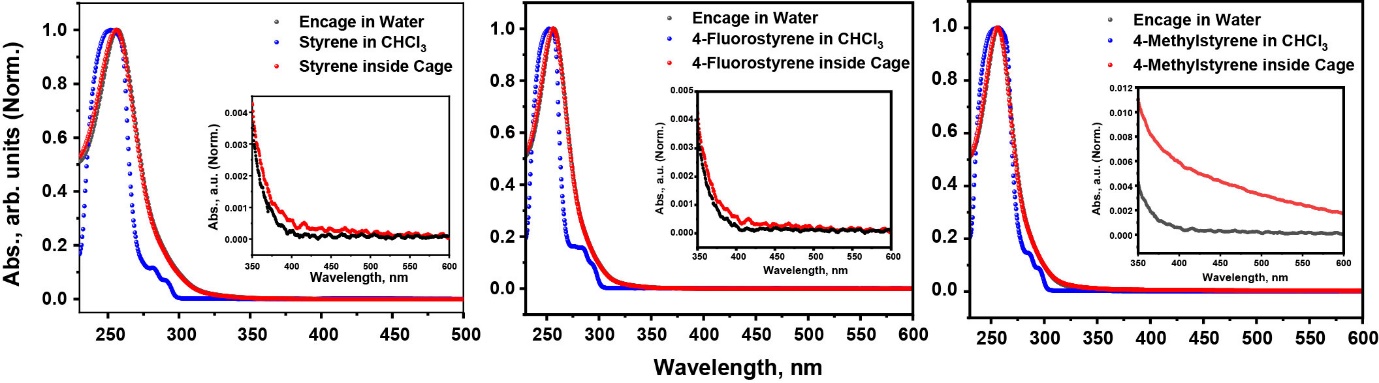


**Supplementary Figure 15.** Steady state absorption spectra of styrene and its derivatives inside Encage in water (in red dots) are compared to free styrene and its derivatives in CHCl_3_ (in blue dots). The Encage absorption is shown also in black dots showing a weak broadening of absorption spectra in the 400 nm region indicating the presence of any transition defined in the complete host-guest basis.


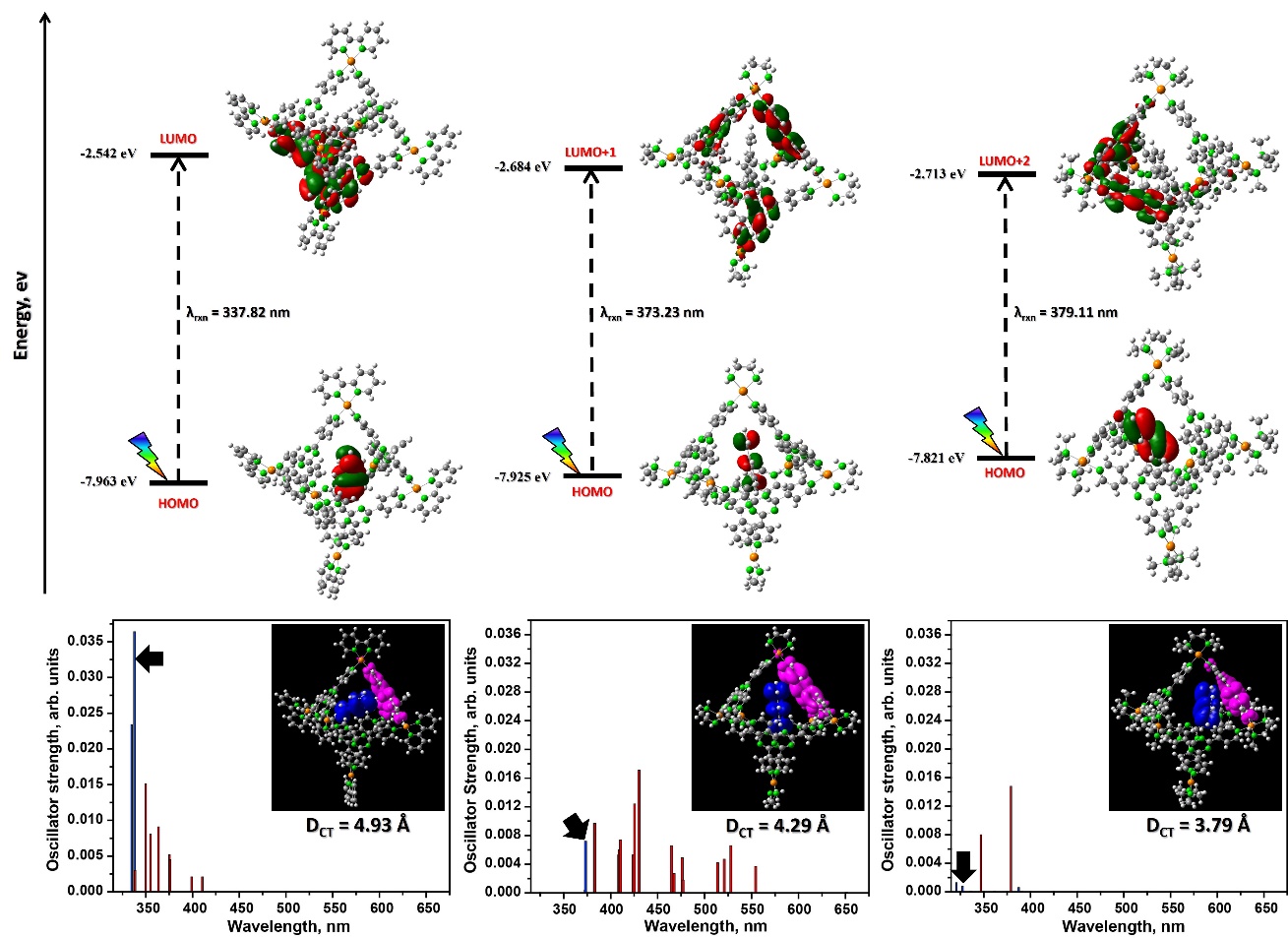


**Supplementary Figure 16.** Excited state calculations of incarcerated styrene complexes for three different Pd_6_L_4_^12+^ cages to predict guest to host charge transfer transitions and characterize those CT transitions with D_CT_ parameter which is the distance between electron displace zone barycenter and electron enrichment zone barycenter; it varies from 3.79 Å to 4.93 Å for three cages.


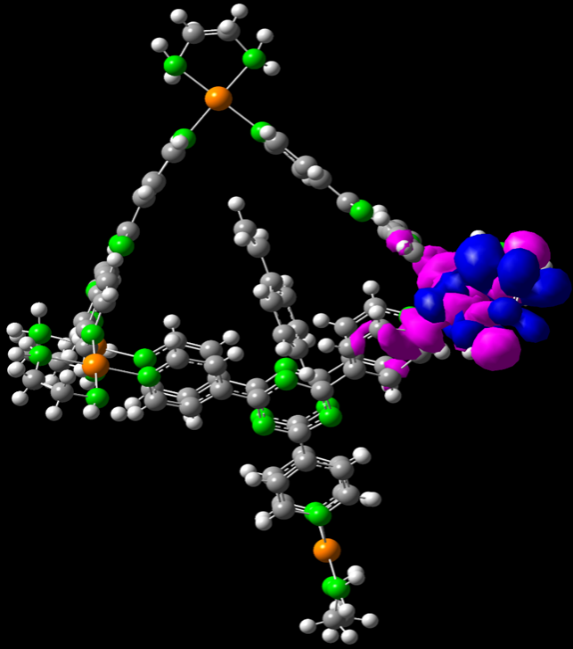


**Supplementary Figure 17.** The difference density plot for the 408 nm excitation energy of the **styrene** $\boldsymbol{\subset}$ **Encage** complex reveals the possibility of cage-centric (Pd site and triazine ligand based) transitions at the 400 nm spectral window along with the other guest to host CT transitions.


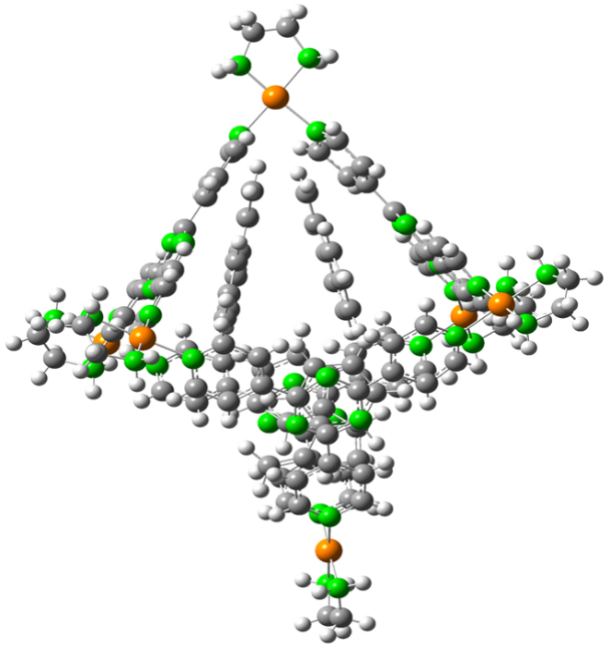


**Supplementary Figure 18.** Optimized geometry for **styrene ⊂ Encage** inclusion complex with 4:1 binding stoichiometry.

**
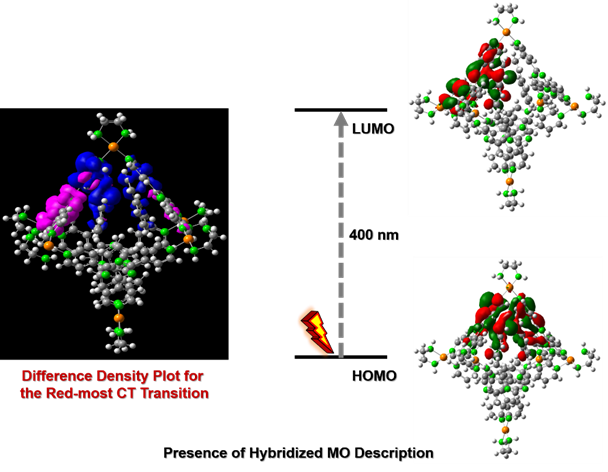
**

**Supplementary Figure 19.** Red-most guest to host charge transfer transition observed at 400.1 nm wavelength obtained through the excited state calculation on **styrene ⊂ Encage** complex with 4:1 binding stoichiometry. The deconvolution of the difference density plot reveals the most contributing MO pairs involved in that transition which can only be described through a hybridized host-guest combined molecular orbital basis.


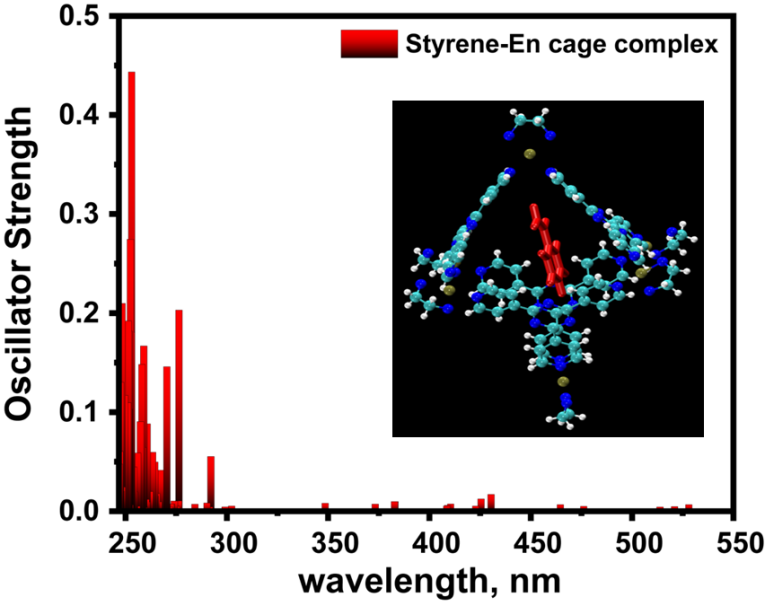


**Supplementary Figure 20.** Simulated absorption spectra for **styrene ⊂ Encage** inclusion complex.


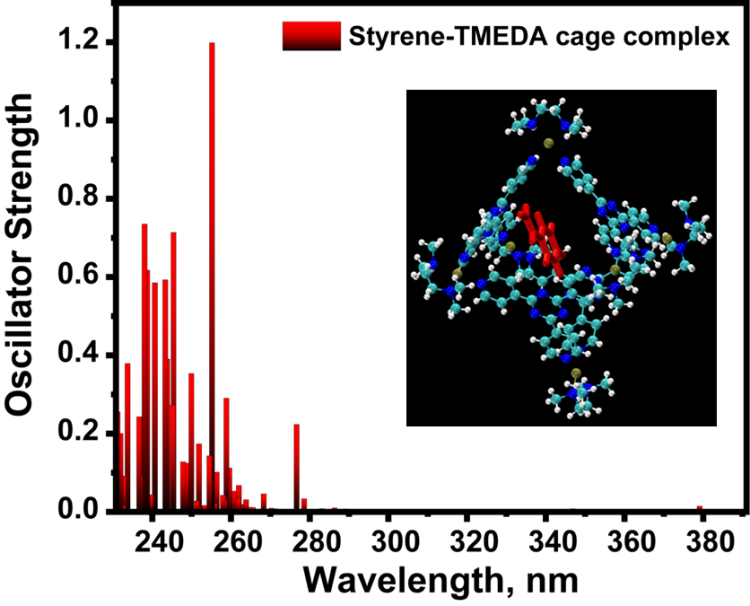


**Supplementary Figure 21.** Simulated absorption spectra for **styrene ⊂ TMEDA cage** inclusion complex.


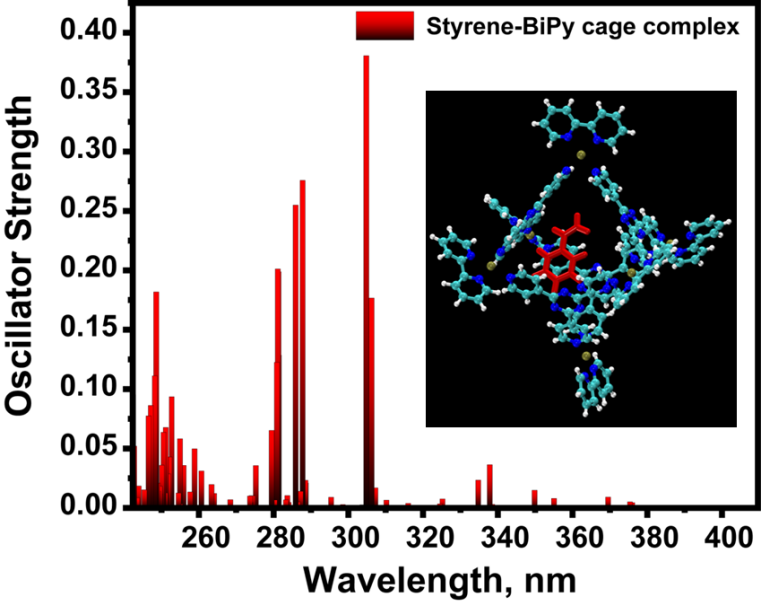


**Supplementary Figure 22.** Simulated absorption spectra for **styrene ⊂ BiPy cage** inclusion complex.


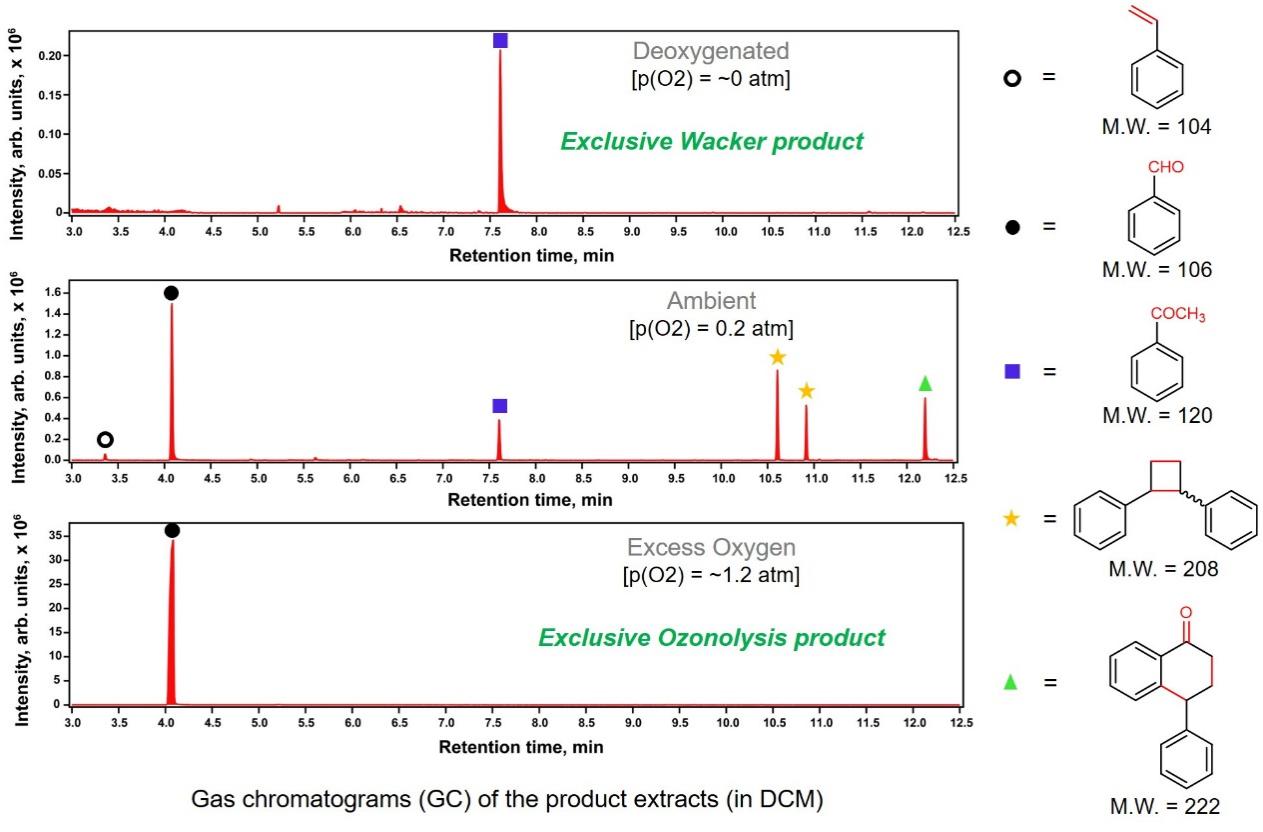


**Supplementary Figure 23.** Comparison of gas chromatograms of organic extracts obtained after the completion of photoreactions of **styrene**$\boldsymbol{\subset}$**Encage** inclusion complexes at deoxygenated condition (freeze-thaw method), ambient condition (0.2 atm) and excess O_2_ (1.2 atm) pressure, respectively. The product selectivity shows an interesting sensitivity on the O_2_ pressure. At deoxygenated condition we observe a single peak at 7.6 min retention time for acetophenone product and at high O_2_ pressure we observe again a single peak at 4.08 min retention time for benzaldehyde formation. While at ambient condition we observe additional peaks at 10.62 min and 10.9 min for the two diastereomers of the [2+2] coupled product and at 12.19 min for the oxidized [4+2] dimer with the peaks for benzaldehyde and acetophenone. The comparison clearly shows that acetophenone is exclusively produced at deoxygenated condition and benzaldehyde is exclusively produced at excess O_2_ pressure; while at ambient condition, dimeric products are additionally forming apart from benzaldehyde and acetophenone.


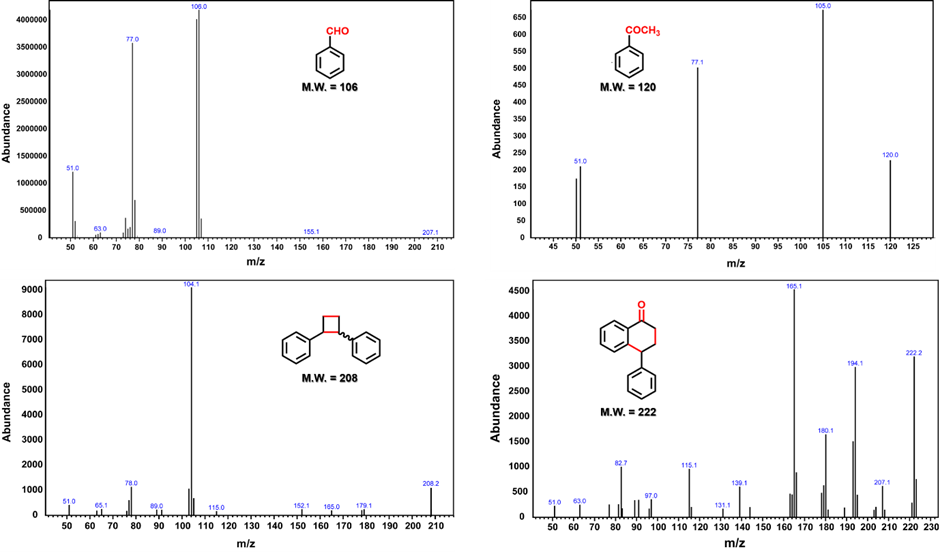


**Supplementary Figure 24.** Mass characterization of the GC fractions [retention times are 4.08 min (top left panel), 7.6 min (top right panel), 10.62 & 10.9 min (bottom left) and 12.19 min (bottom right panel) respectively] of the product extract obtained from styrene photo-reaction inside En cage with 400 nm light illumination at ambient condition.


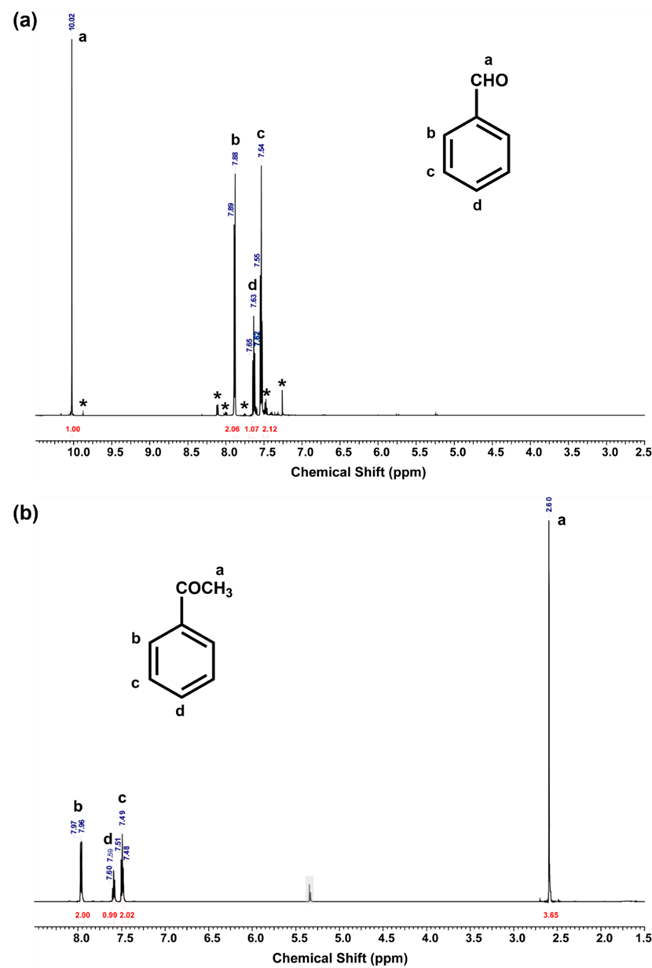


**Supplementary Figure 25.** ^1^H-NMR (800 MHz, CD_2_Cl_2_) characterization of the product extract obtained from the photo-reaction of styrene at high O_2_ pressure (a) and at deoxygenated condition (b) respectively. This clearly shows that under excess oxygen pressure exclusive benzaldehyde formation is taking place while at the deoxygenated condition exclusive acetophenone forms.


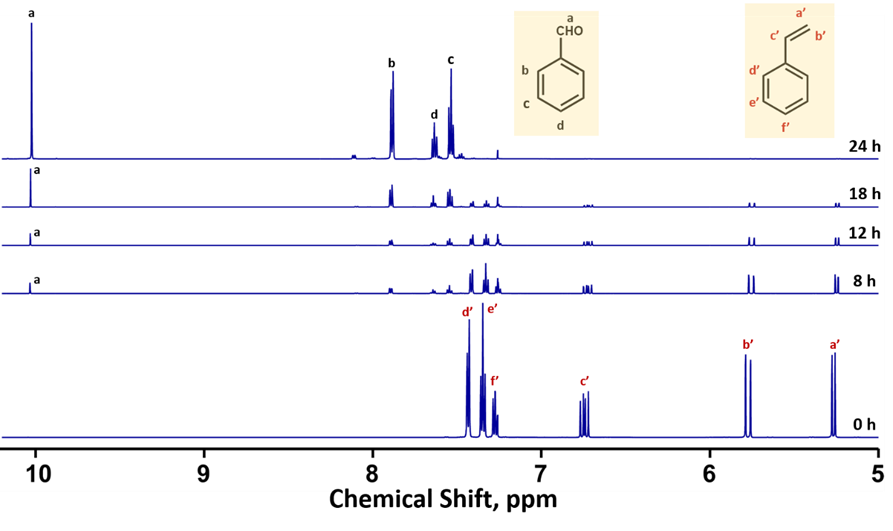


**Supplementary Figure 26.** ^1^H NMR spectra (800 MHz, CD_2_Cl_2_) taken at different timepoints during catalytic transformation of styrene into benzaldehyde. Same volume of filtered aliquots was taken from the reaction mixture at different timepoints, DCM extraction was performed, then dried, redissolved in CD_2_Cl_2_ and ^1^H NMR was taken. The marker aldehyde proton peak at 10.02 ppm enhances its intensity while the marker olefinic proton peaks for styrene at 5.6 and 5.72 ppm shows a correlated decrease in intensity with the advancement of reaction. At 24 h of reaction time, the 50 catalytic turn-over reaches to its completion.

***
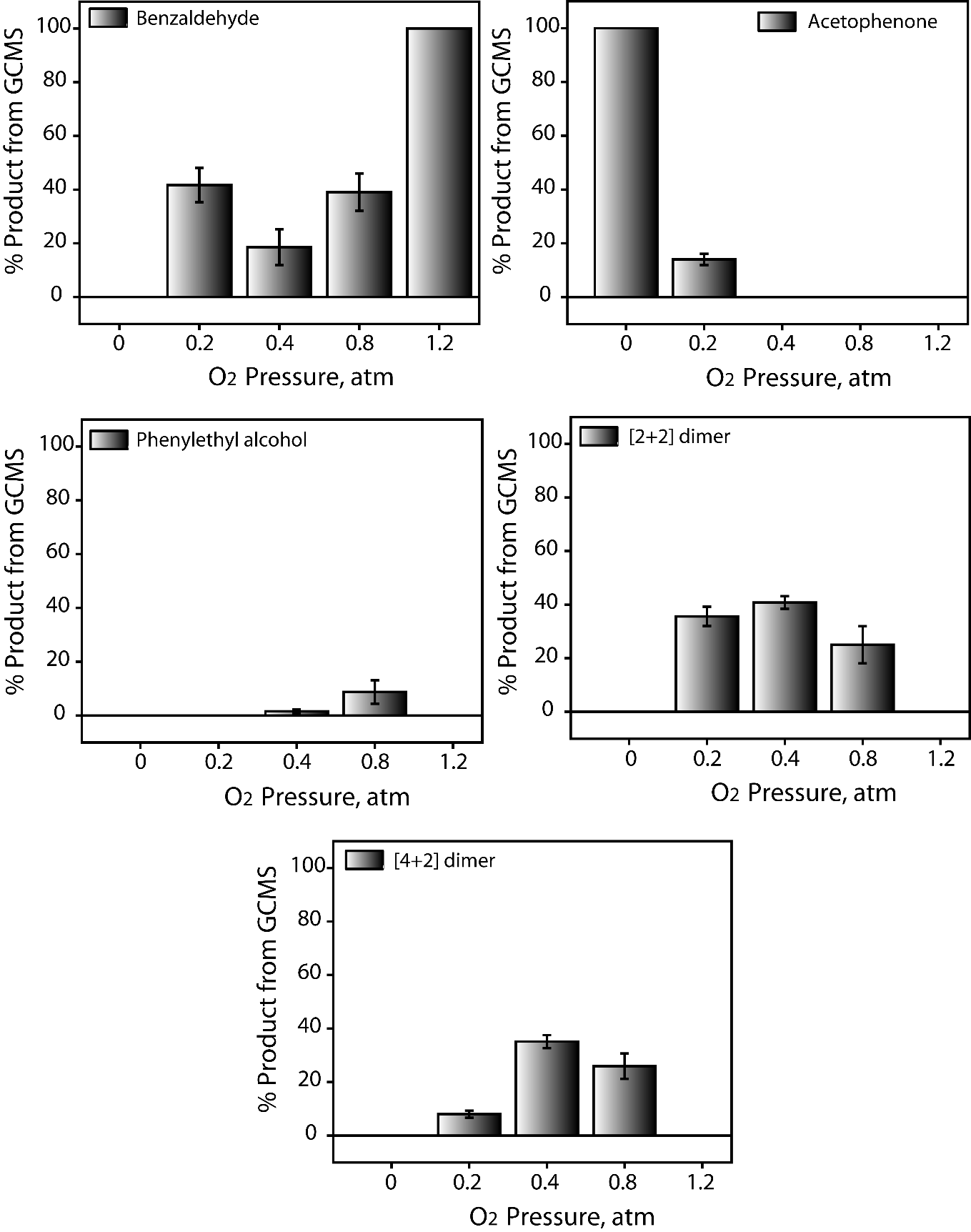
***

**Supplementary Figure 27.** Variation in the percentage of photoproducts under different O_2_ concentrations, derived from GCMS measurements.


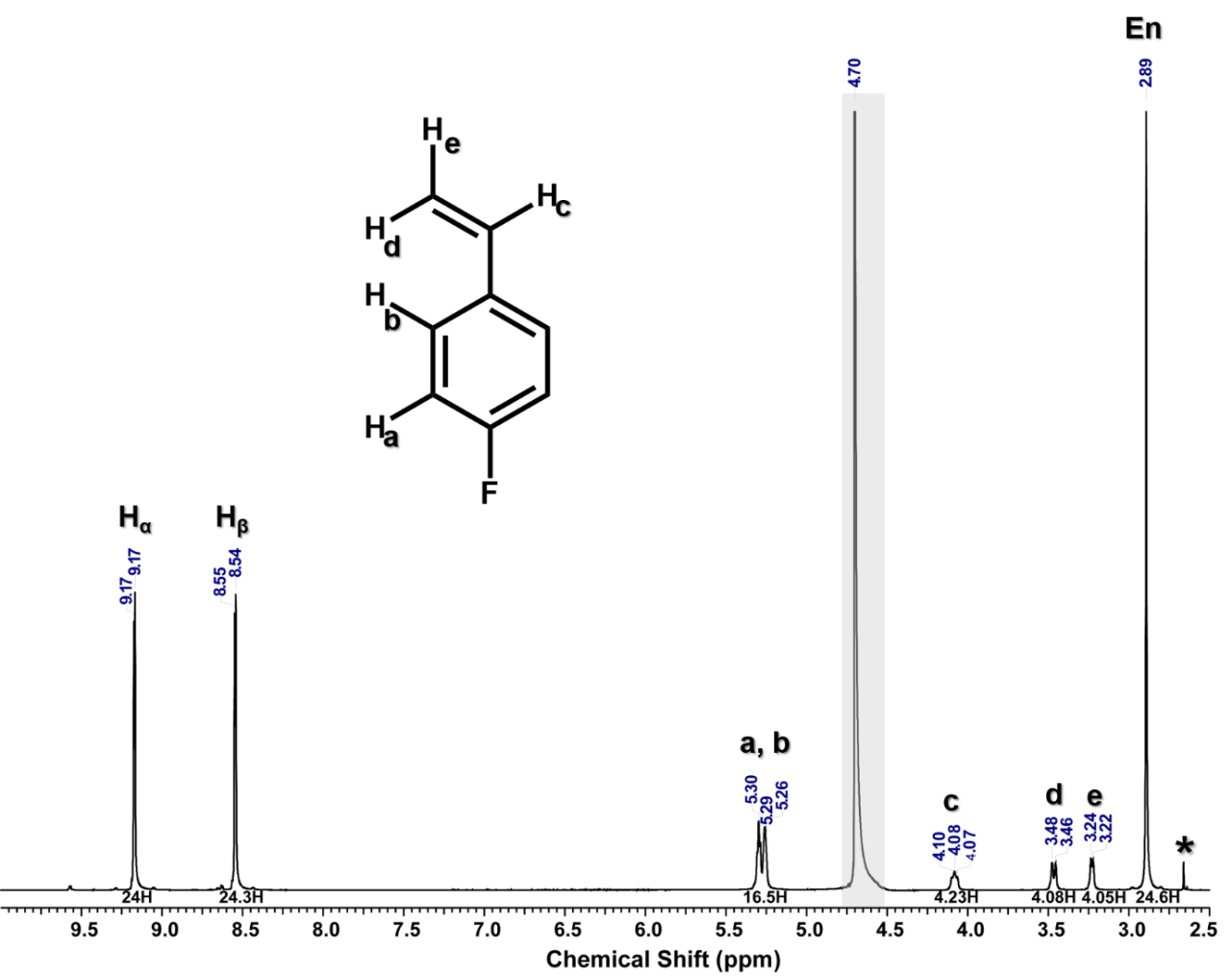


**Supplementary Figure 28.** ^1^H NMR spectrum of **4-fluorostyrene**$\boldsymbol{\subset}$**Encage** (800 MHz, D_2_O, 298K) inclusion complex showing clear upfield shift of 4-fluorostyrene proton peaks with respect to those for the free 4-fluorostyrene molecule (reference to Supplementary Fig 10). Further, peak integration ratio with respect to the host protons reflects 1:4 host:guest binding stoichiometry on an average.


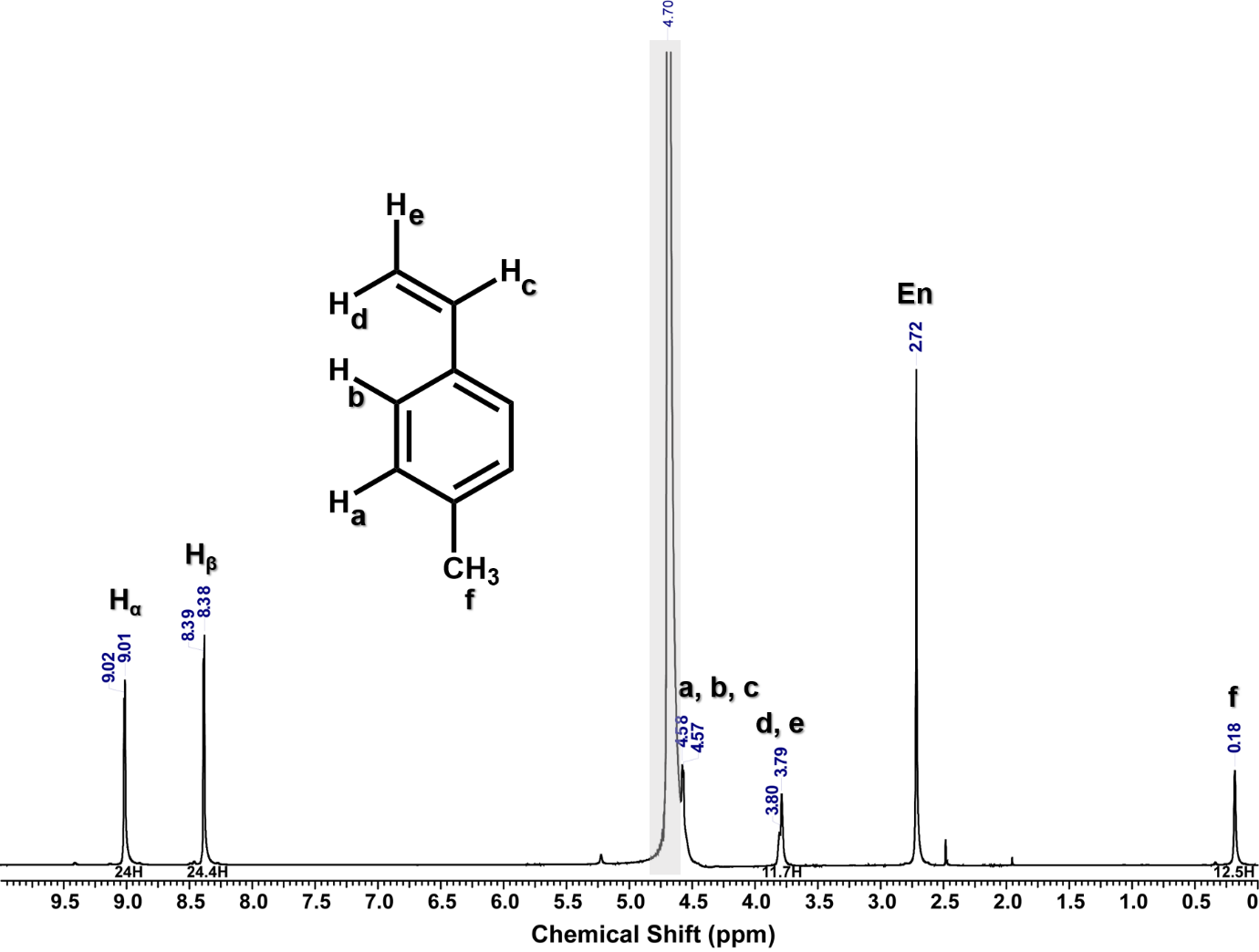


**Supplementary Figure 29.** ^1^H-NMR spectrum of **4-methylstyrene**$\boldsymbol{\subset}$**Encage** (800 MHz, D_2_O, 298K) inclusion complex showing clear upfield shift of 4-methylstyrene proton peaks with respect to those for the free 4-methylstyrene molecule (reference to Supplementary Fig 11). Further, peak integration ratio (methyl proton of guest appearing at 0.15 ppm chemical shift) with respect to the host protons reflects 1:4 host:guest binding stoichiometry on an average.


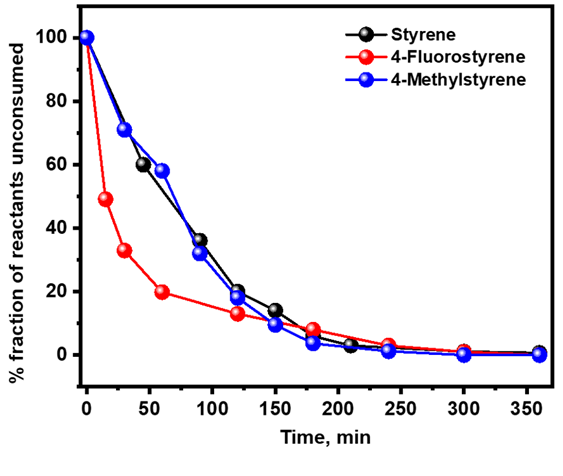


**Supplementary Figure 30.** Comparative rates of substrate consumption for styrene, 4-methylstyrene and 4-fluorostyrene showing the faster rate of reaction for the fluoro-derivative.


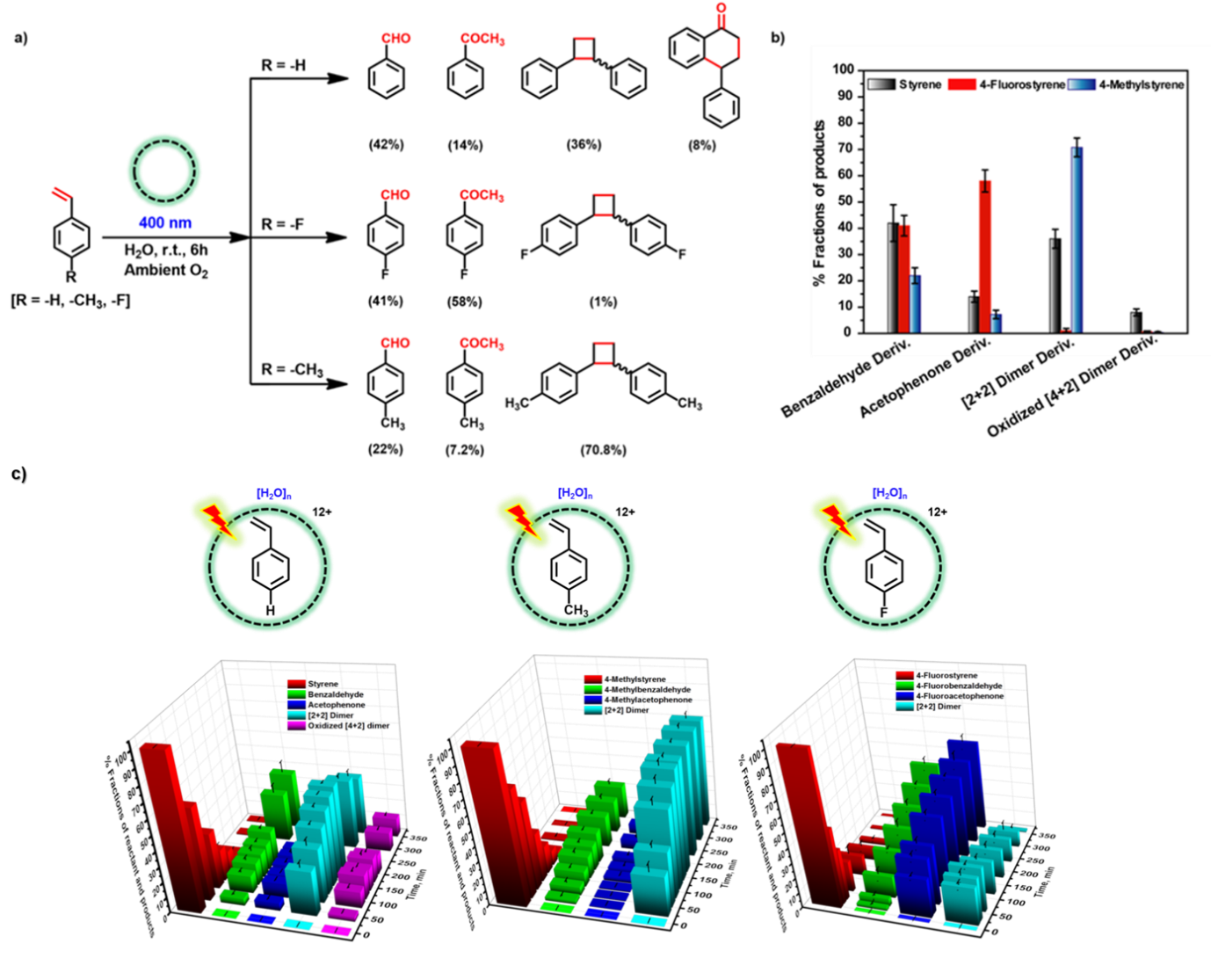


**Supplementary Figure 31.** The comparative product distribution for ambient photo-oxidation of styrene and its derivatives, both (a) in schematics and (b) in bar plot. The attachment of hydrophilic functional group (like -F) exposes 4-fluorostyrene more towards interfacial water at the pores leading to the water-addition mediated acetophenone formation as the major product. While the incorporation of hydrophobic functionality (like -CH_3_) buries styrene molecules more towards the center of the cavity leading to the inter-guest proximity amongst styrene molecules incarcerated inside one host. This consequences in the formation of more dimeric products for 4-methylstyrene. This demonstrates a nice control on reactivity in confined space by tweaking the host-guest preorganization through introducing variable functionalities.


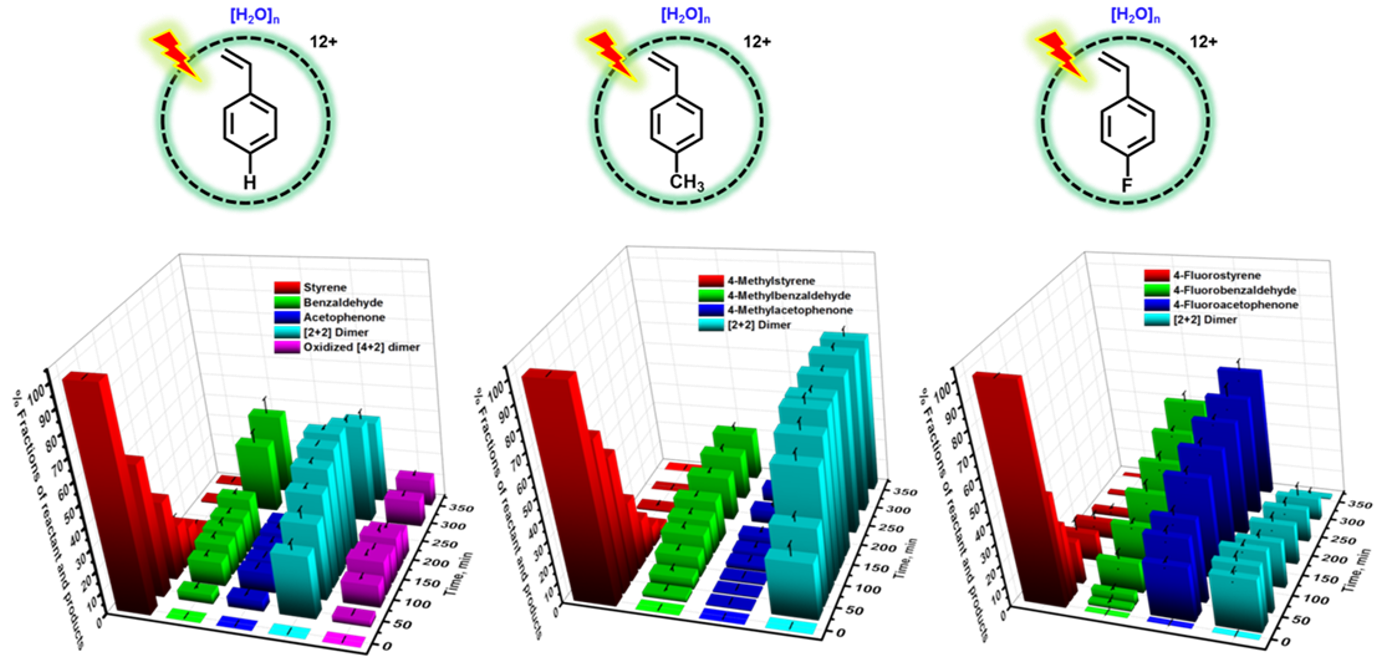


**Supplementary Figure 32.** Tuning the selectivity of photo-functionalization by introducing hydrophilic and hydrophobic functional groups in styrene: For 4-fluorostyrene, water addition mediated acetophenone formation was observed to be the major pathway while for 4-methylstyrene, intermolecular C-C coupling mediated dimer formation was observed to be the major reaction channel. Comparison amongst the time-courses of photoproduct formation with the advancement of reaction clearly shows faster rate of dimer formation for 4-methylstyrene and faster rate of acetophenone formation for 4-fluorostyrene. The variation of product distribution along with the different kinetic partitioning strongly emphasizes the influence of host-guest preorganization in confinement-chemistry.


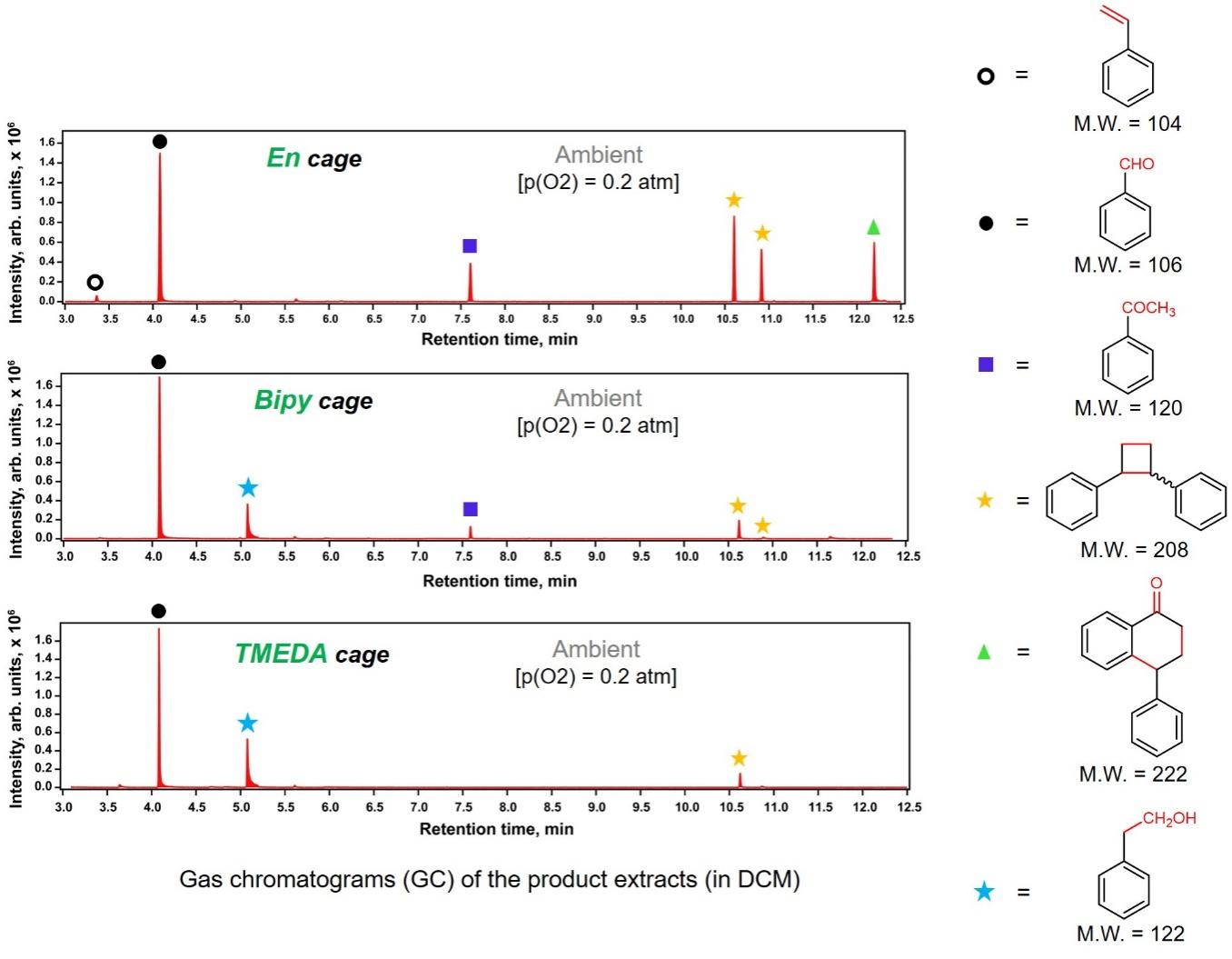


**Supplementary Figure 33.** Comparison of gas chromatogram of organic extracts obtained after the completion of photoreactions of **styrene**$\boldsymbol{\subset}$**nanocage** inclusion complexes for three different cages namely En cage, TMEDA cage and BiPy cage at ambient condition. The comparison clearly shows the influence of cage alteration and subsequent guest preorganization on the photo-selectivity. For En cage we obtained mostly benzaldehyde, acetophenone and [2+2] dimer as the major products; while for TMEDA and BiPy cages, we obtained mostly benzaldehyde and 2-phenyl-1-ethanol as the two major products.


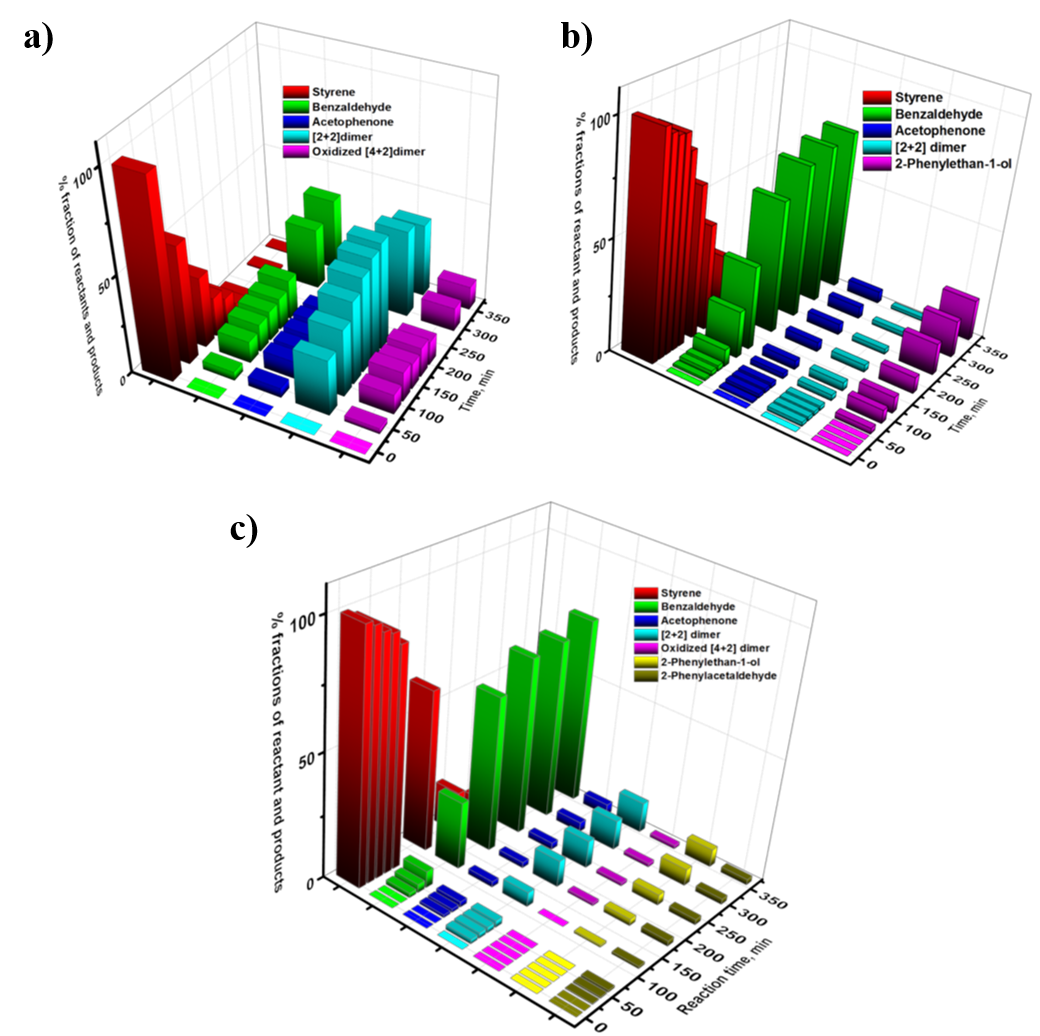


**Supplementary Figure 34.** The styrene consumption and product formation kinetics for ambient photo-oxidation of styrene in three different cages: (a) En cage, (b) TMEDA cage and (c) BiPy cage respectively. In TMEDA and BiPy cages, benzaldehyde formation kinetics is associated with an initial prominent fast phase which ultimately leads to benzaldehyde as the major product. While in En cage, the dimeric product forms with a larger fraction correlated with its fast rise kinetics.


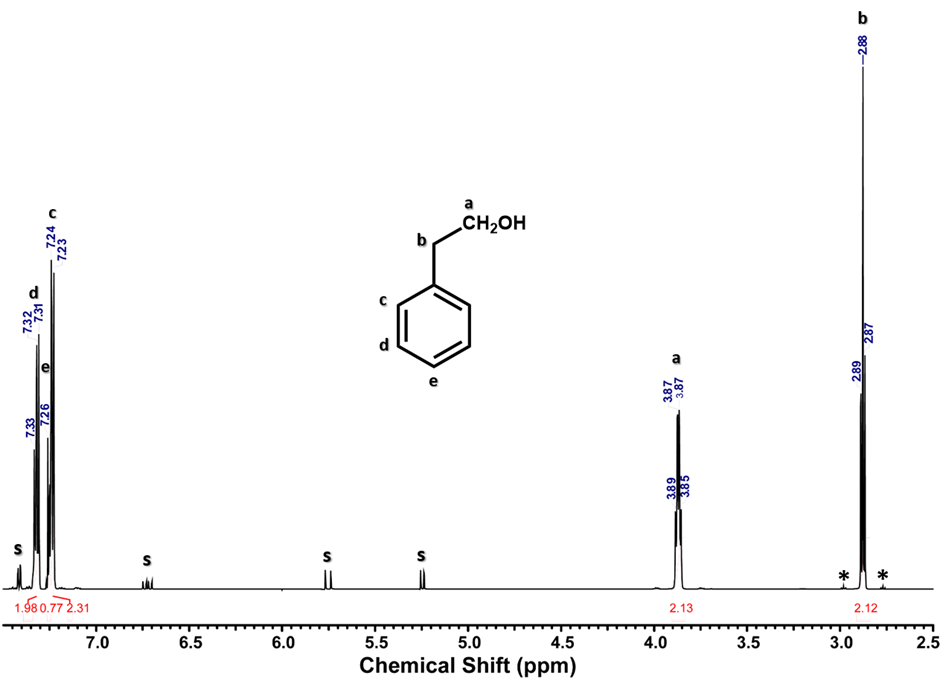


**Supplementary Figure 35.** ^1^H NMR characterization (800 MHz in CD_2_Cl_2_) of the crude organic extract of the reaction mixture obtained after 6 hours of photo-reaction on **styrene**$\boldsymbol{\subset}$ **TMEDA** inclusion complex under Ar atmosphere. It clearly shows the aromatic and aliphatic proton peaks of **2-phenyl-1-ethanol** and trace amount of unconsumed styrene proton peaks, marked by ‘s’.


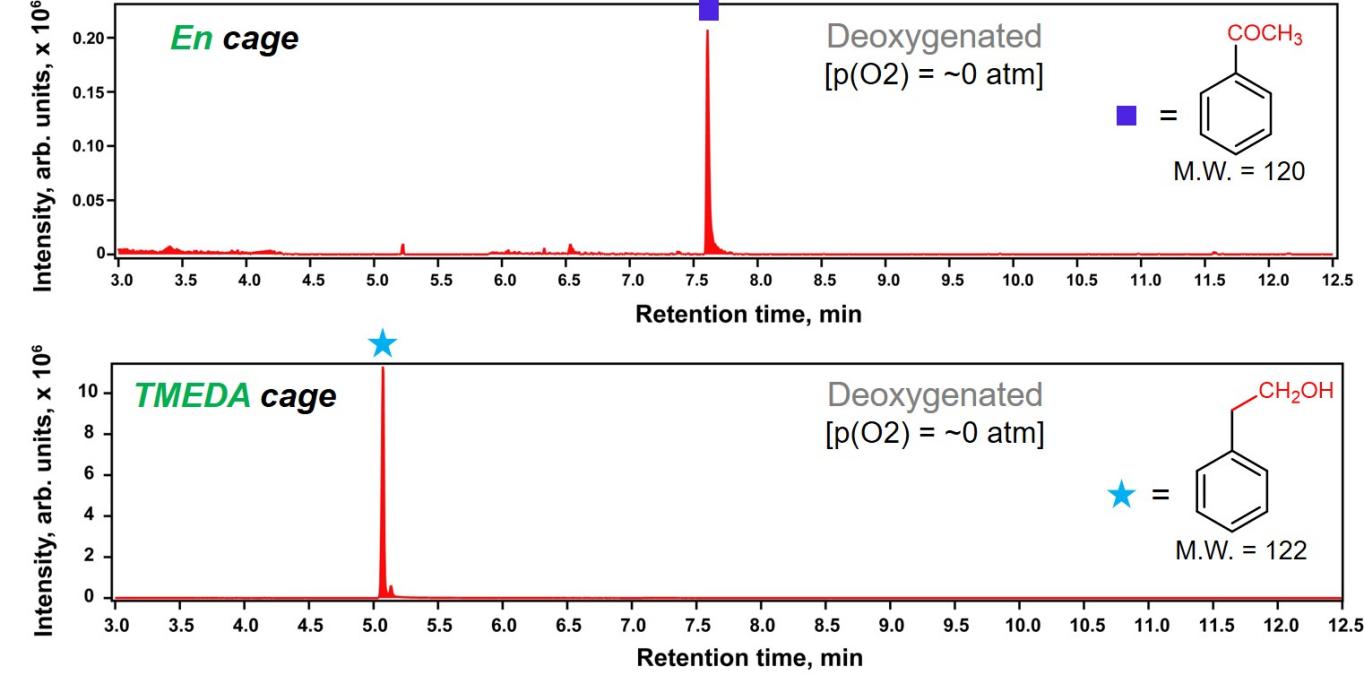


**Supplementary Figure 36.** Comparative GC chromatogram showing the formation of acetophenone and 2-phenylethanol as the photo-products obtained from styrene photo-oxidation at deoxygenated condition inside En and TMEDA cages respectively showing a beautiful control on product selectivity upon host structure alteration.


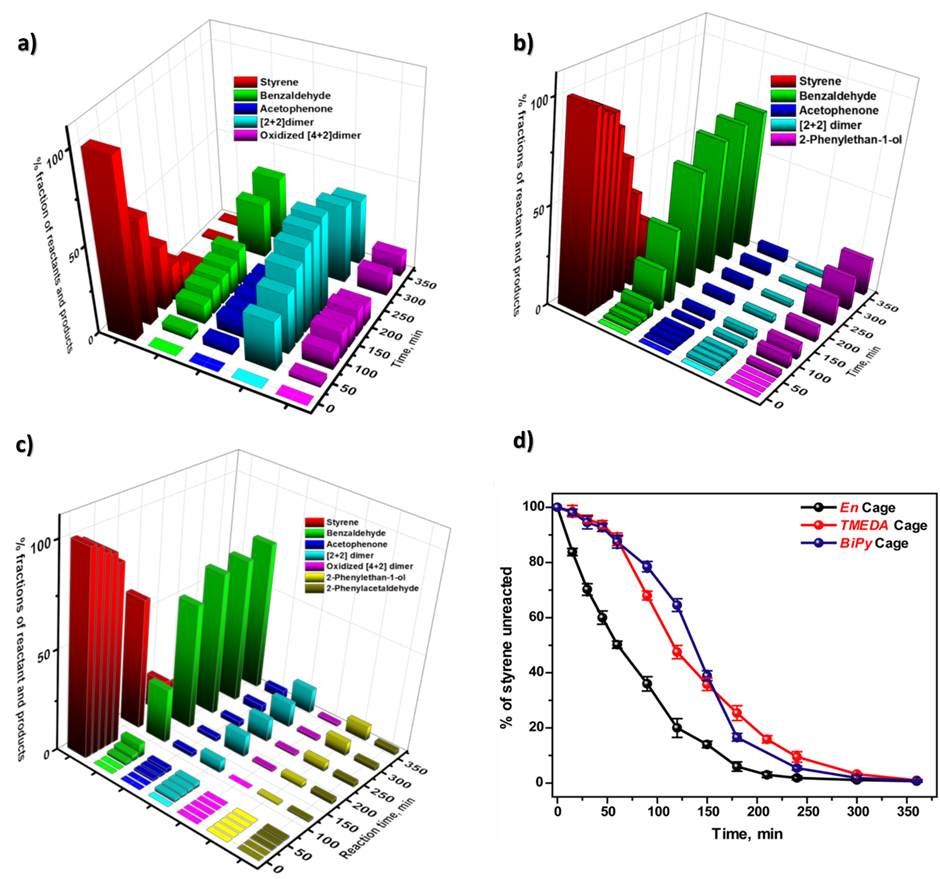


**Supplementary Figure 37.** The influence of the alteration of host ancillary ligands (En to TMEDA and BiPy) in the reaction kinetics. The styrene consumption follows an exponential decay kinetics for En cage while it obeys a sigmoidal decay kinetics for TMEDA and BiPy cages with an initial lag phase.


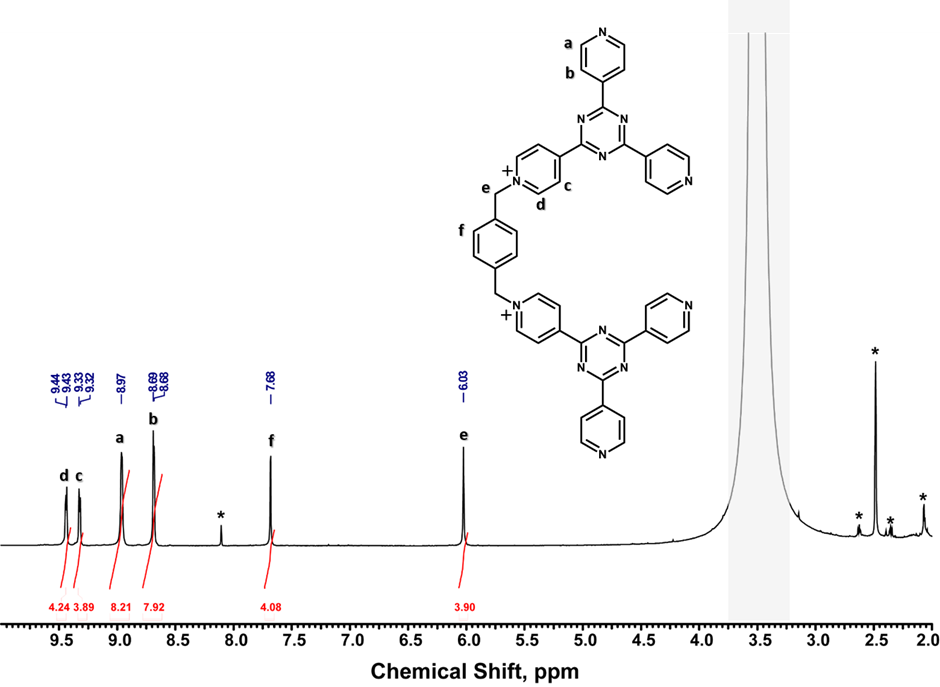
**Supplementary Figure 38.** ^1^H-NMR characterization of the synthesized organic ligand L’ (800 MHz, DMSO-d_6_, 298K).


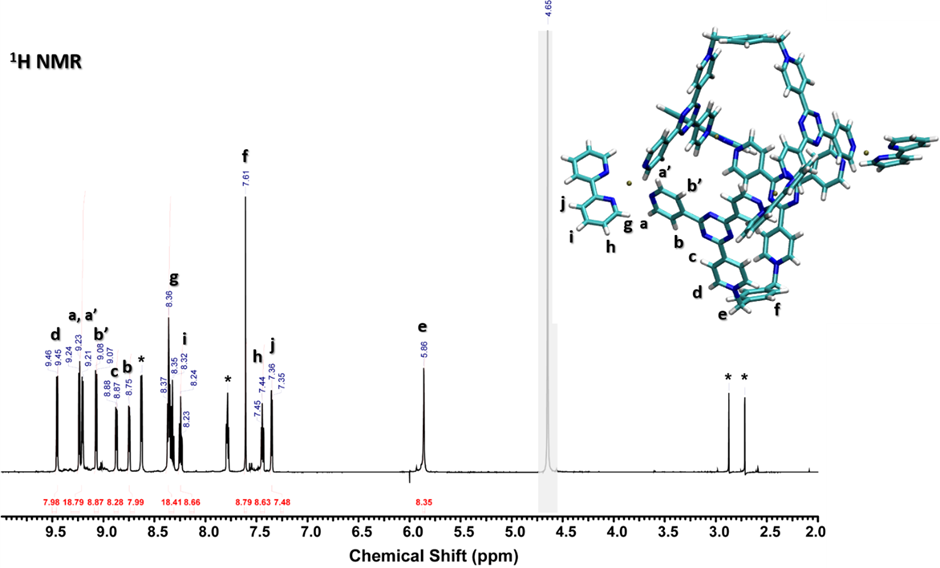


**Supplementary Figure 39.** ^1^H-NMR characterization of the synthesized Pd_4_L’_2_^12+^ Nanocage (800 MHz, D_2_O, 298K).


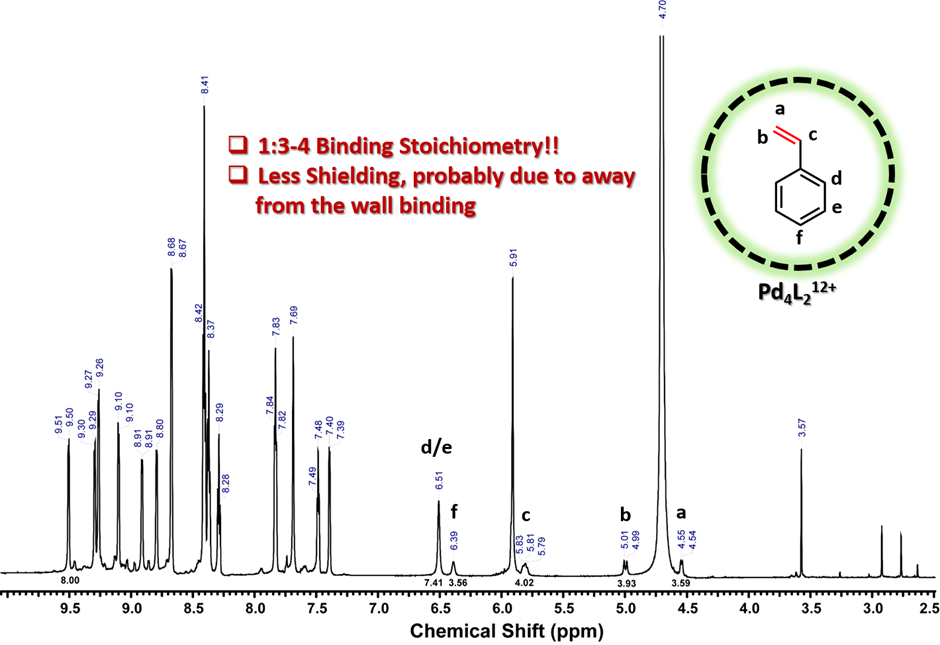


**Supplementary Figure 40.** ^1^H-NMR Characterization of **styrene**$\boldsymbol{\subset}$**Pd_4_L’_2_^12+^** host-guest inclusion complex (800 MHz, D_2_O, 298K) in which the styrene proton peaks are clearly assigned with the corresponding peak integration in addition to the cage proton peaks. The peak integration shows that three to four styrene molecules are getting incarcerated inside the nanocage.


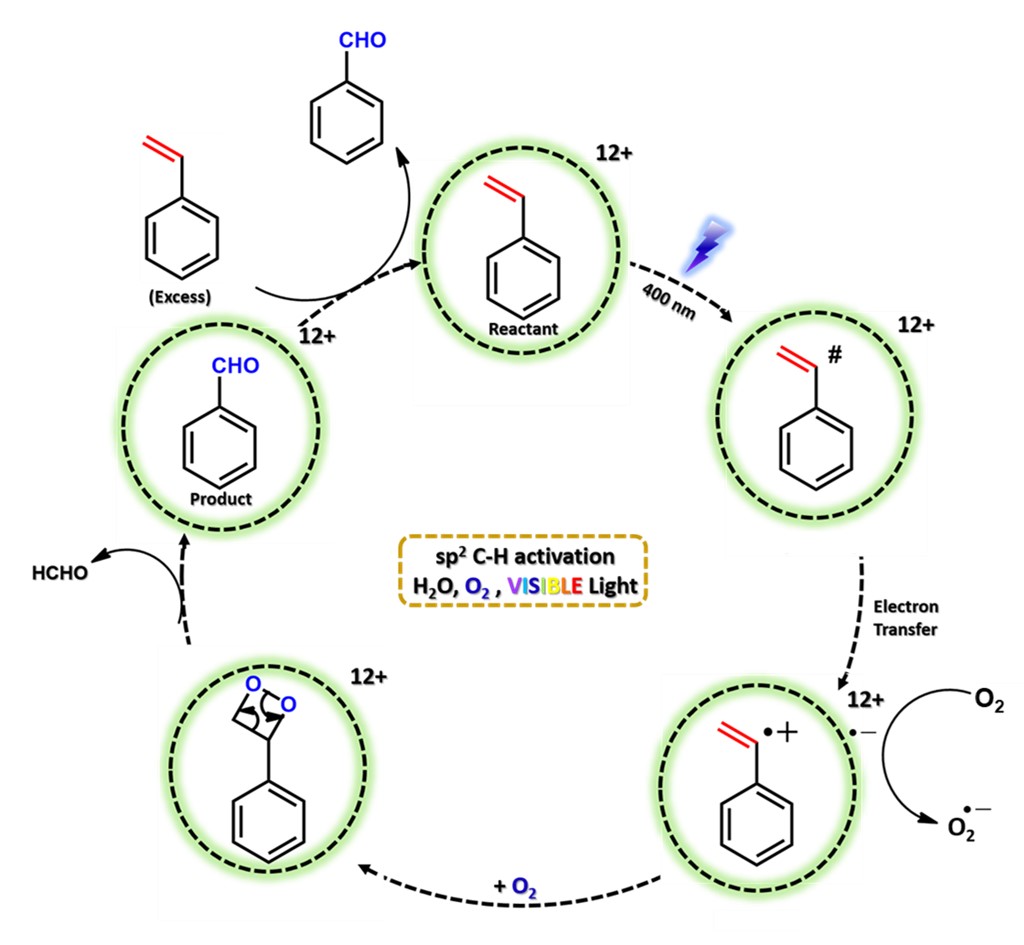


**Supplementary Figure 41.** The proposed photo-catalytic cycle for the catalytic conversion of styrene to benzaldehyde by water, dioxygen and visible light as the green reagents. The light activation step involves styrene to cage electron transfer leading to the formation of styrene radical cation as the transient photo-intermediate; while the subsequent light-independent dark reaction steps involves bimolecular collision of styrene radical cation with freely diffusing dioxygen molecule leading to formation of benzaldehyde as the final photo-product. Subsequently excess styrene outside the cavity displaces the slightly more hydrophilic benzaldehyde from the cage leading to the turnover of the catalyst. The dioxygen molecule has another crucial role for taking the excess electron (coming from the guest) off the cage wall to make the cage ready for another photo-redox reaction with another styrene and thus the process becomes catalytic. In absence of dioxygen, we never get such photo-catalytic turn-over supporting our hypothesis of the additional role of dioxygen as the terminal sacrificial electron acceptor.


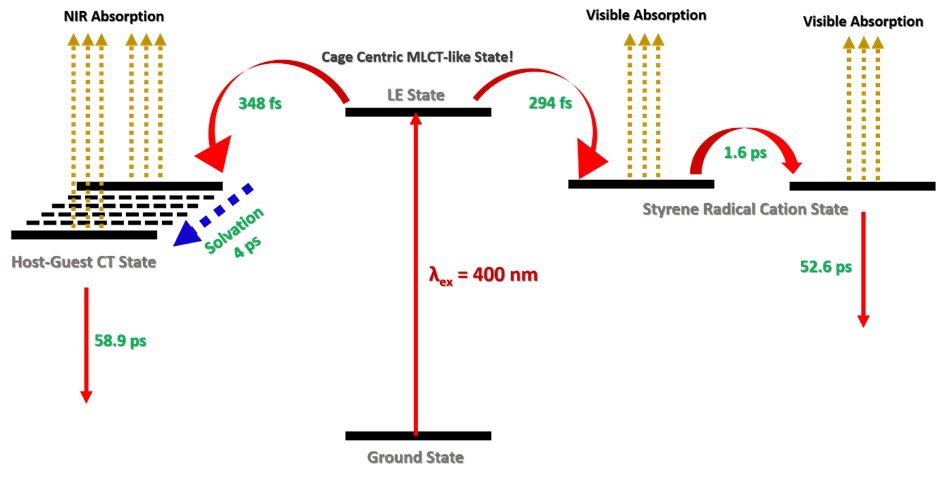


**Supplementary Figure 42.** The proposed kinetic scheme for the excited state dynamics of the **styrene** $\boldsymbol{\subset}$ **Encag**e. The initial 400 nm excitation leads to formation of the styrene radical cation as well as the host-guest centric CT states.


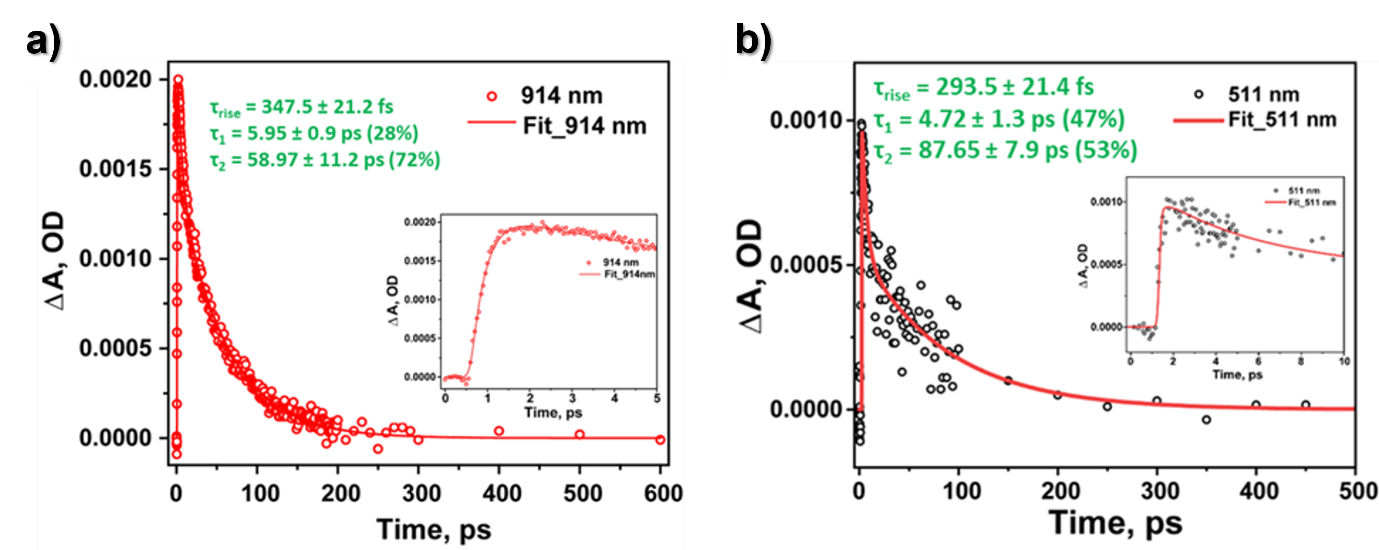


**Supplementary Figure 43.** Single point spectral kinetics for **styrene** $\boldsymbol{\subset}$ **Encage** complex (a) at 914 nm indicates the population dynamics of the host-guest CT state while (b) at 511 nm represents the population dynamics of the styrene radical cation population. The associated lifetimes are mentioned in the plots indicating of styrene CT lifetime as below 100 ps inside En cage.


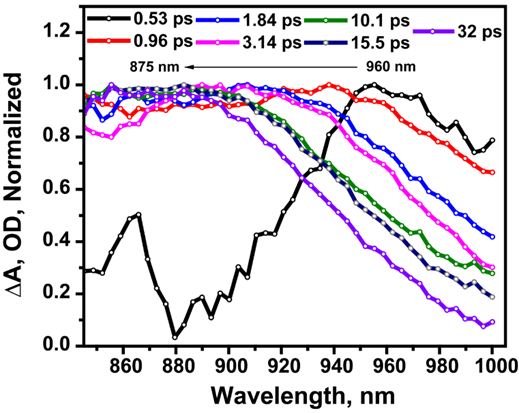


**Supplementary Figure 44.** The blue-shift in the transient spectra traces from 960 nm to 875 nm within few picoseconds after photoexcitation of the **styrene** $\boldsymbol{\subset}$ **Encage** complex signifies solvation.


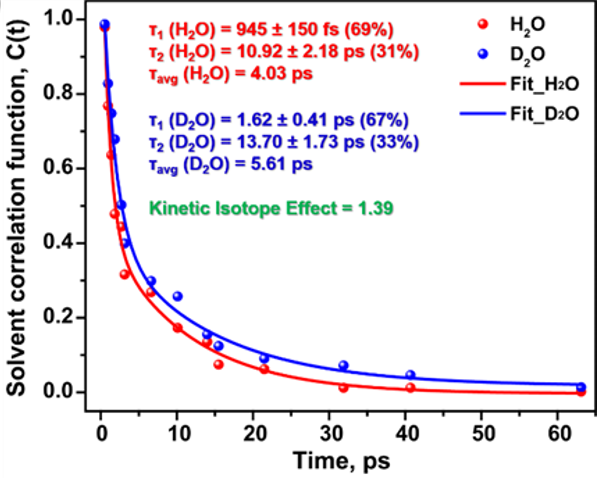


**Supplementary Figure 45.** The comparison of the solvent correlation function plots with time analysed from the NIR spectral data for **styrene** $\boldsymbol{\subset}$ **Encage** complex in H_2_O and D_2_O unequivocally shows a clear solvation kinetic isotope effect of 1.39.


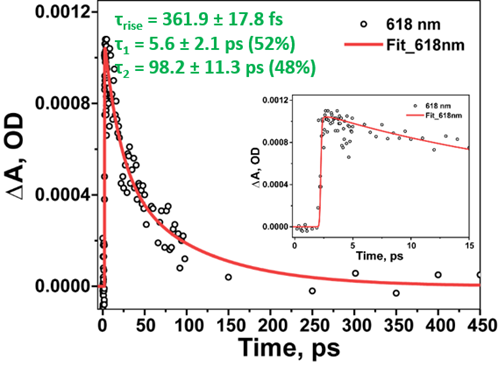


**Supplementary Figure 46.** Spectral kinetics of **styrene** $\boldsymbol{\subset}$ **Encage** inclusion complex at 618 nm shows a prominent rise time of 362 fs followed by a 6 ps decay component (52% contributing) and a 98 ps decay component (48% contributing).


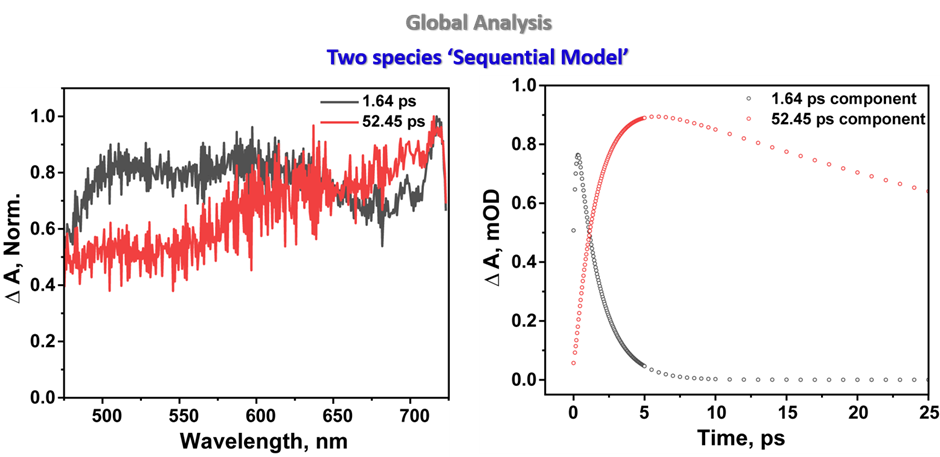


**Supplementary Figure 47.** The normalized species associated spectra obtained from the SVD (Singular value decomposition) analysis on the transient absorption signal matrix in the visible spectral regime for **styrene** $\boldsymbol{\subset}$ **Encage** inclusion complex. The spectral deconvolution by the global analysis shows the best fit with the ‘two-species sequential model’ predicting an initial 1.64 ps long species and the decay of that species gives rise to another species having 52.45 ps lifetime. The corresponding kinetics is also shown for the two species absorbing in the same region.


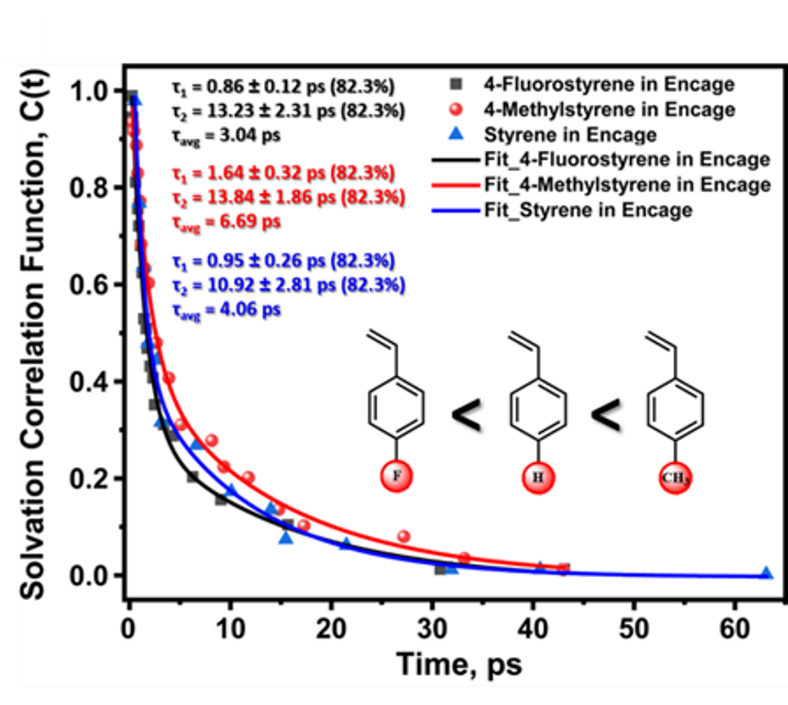


**Supplementary Figure 48.** Comparison of solvation dynamics for styrene and its derivatives inside Encage shows the fastest solvation dynamics for the **4-fluorostyrene ⊂ Encage** complex while the **4-methylstyrene ⊂ Encage** complex shows a slow solvation. The data reveals more solvent exposure for the 4-fluorostyrene while 4-methylstyrene is possibly the most buried inside the En cage.


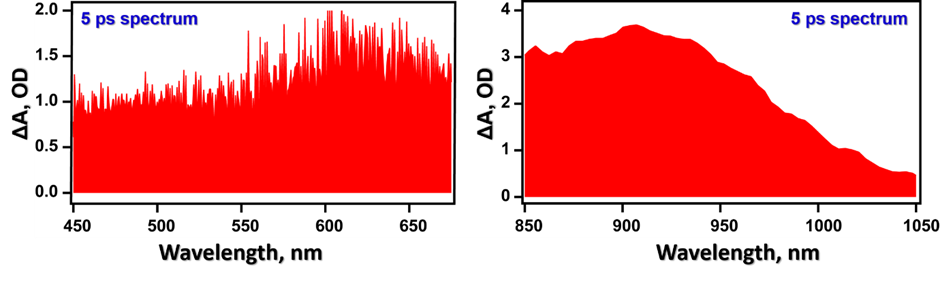


**Supplementary Figure 49.** Transient absorption spectrum of **4-methylstyrene** $\boldsymbol{\subset}$ **Encage** inclusion complex both in visible spectral window as well as NIR spectral region. Visible regime shows the 4-methylstyrene radical cation absorption as a broad weak feature and NIR region shows the absorption of the host-guest coupled CT state. Both of the representative spectra are transient signals obtained at 5 picosecond delay.


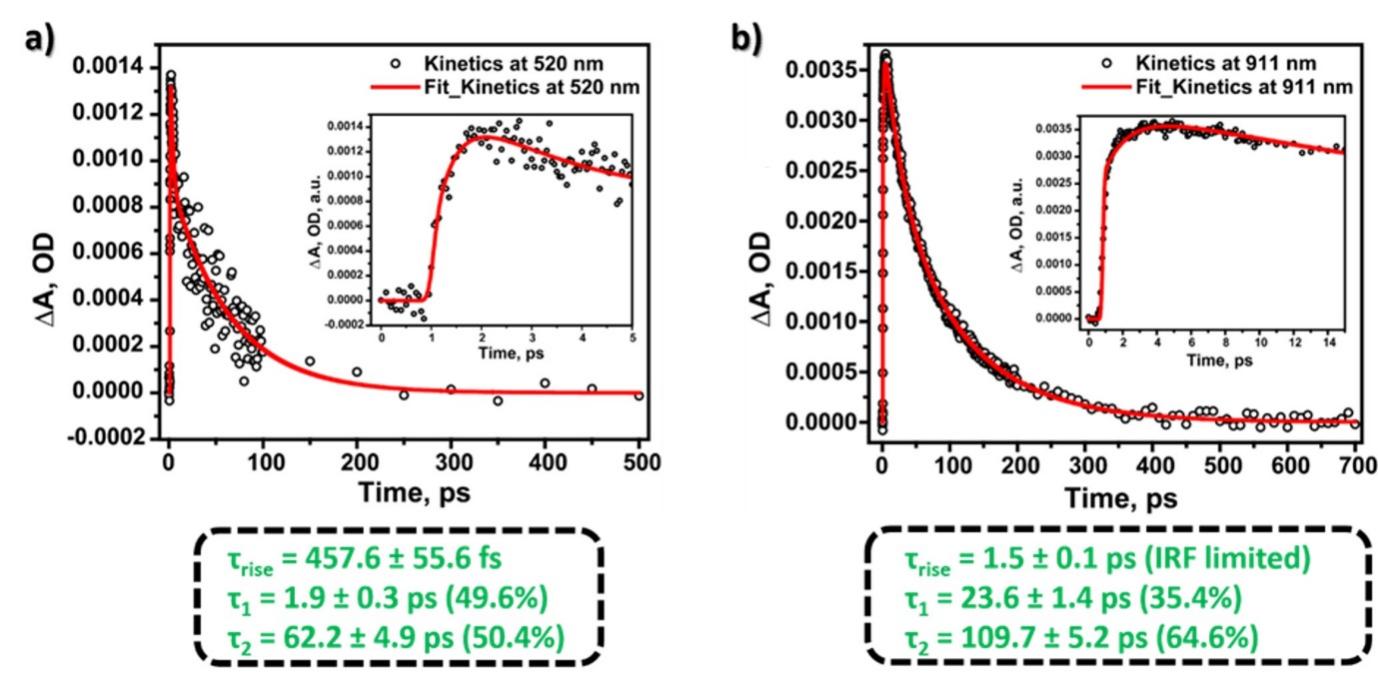


**Supplementary Figure 50.** Single point spectral kinetics (a) at 520 nm for 4-methylstyrene radical cation state absorption in the visible as well as (b) at 911 nm for **4-methylstyrene ⊂ Encage** CT state absorption at NIR regime. The associated time components are mentioned accordingly.


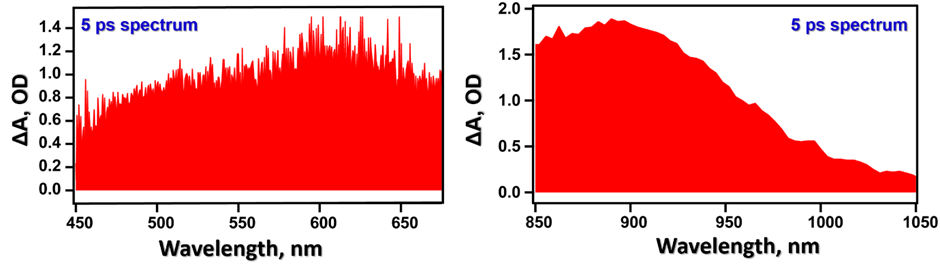


**Supplementary Figure 51.** Transient absorption spectrum of **4-fluorostyrene** $\boldsymbol{\subset}$ **Encage** inclusion complex both in visible spectral window as well as NIR spectral region. Visible regime shows the 4-fluorostyrene radical cation absorption as a broad weak feature and NIR region shows the absorption of the host-guest coupled CT state. Both of the representative spectra are transient signals obtained at 5 picosecond delay.


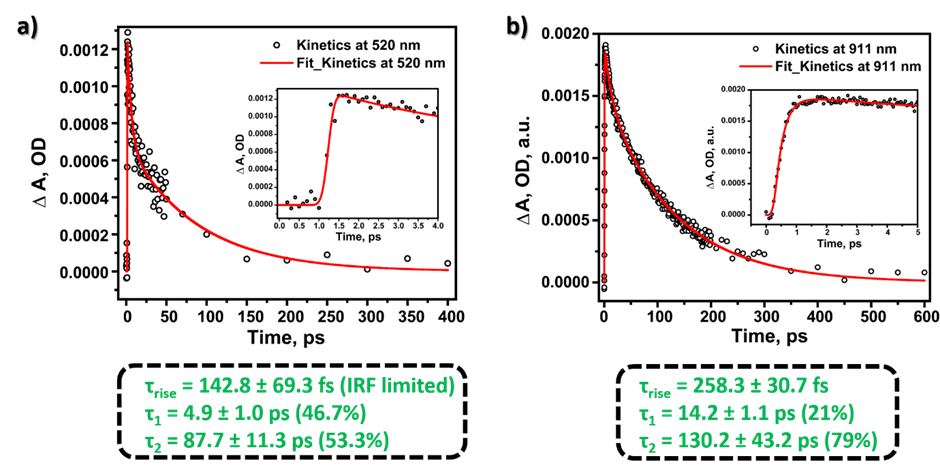


**Supplementary Figure 52.** Single point spectral kinetics (a) at 520 nm for 4-fluorostyrene radical cation state absorption in the visible as well as (b) at 911 nm for **4-fluorostyrene ⊂ Encage** CT state absorption at NIR regime. The associated time components are mentioned accordingly.


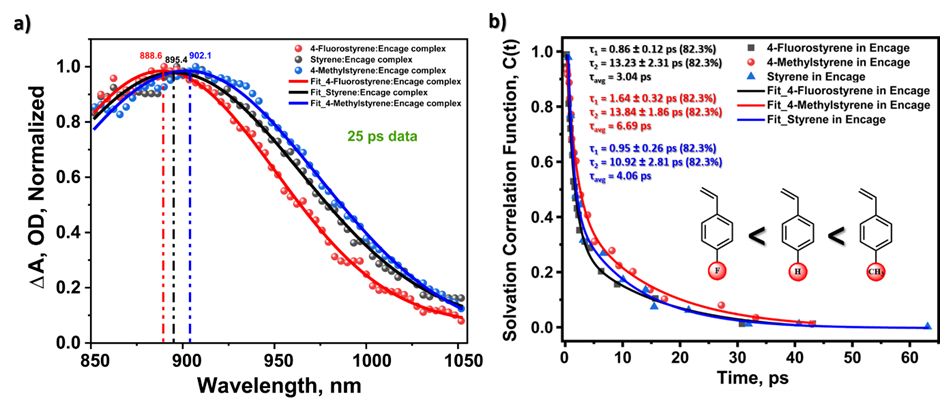


**Supplementary Figure 53.** The comparison of the associated host-guest CT spectra for styrene and its derivatives after solvation which shows the most solvent-stabilized CT state for **4-fluorostyrene ⊂ Encage** complex (red) reflected from the blue-most absorption peak maxima (~889 nm) while the least solvent-stabilized CT state for **4-methylstyrene ⊂ Encage** complex (blue) reflected from the red-most absorption peak maxima (~902 nm).


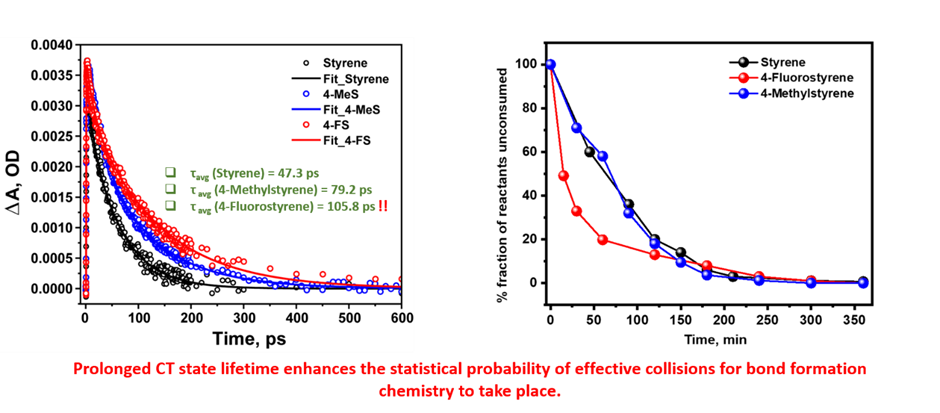


**Supplementary Figure 54.** The comparative spectral kinetics of host-guest CT absorption feature at NIR spectral regime for three different guest molecules: styrene and its two derivatives. This shows that **4-fluorostyrene ⊂ Encage** coupled CT is the most long-lived one (τ_avg_ = 106ps) while the **styrene ⊂ Encage** coupled CT is the least stable (τ_avg_ = 47 ps) due to fastest charge recombination and **4-methylstyrene ⊂ Encage** coupled CT lifetime falls in between (τ_avg_ = 79 ps). We have a remarkable correlation in between the CT state lifetime and the rate of photocatalysis reactions. 4-fluorostyrene having a substantially high CT state lifetime in comparison to the other derivatives consumes in a faster rate than the other two derivatives which indicates that prolonged CT lifetime probably allows more frequent effective bimolecular collisions with H_2_O or O_2_ to enhance the effective reaction yield. However, this CT state lifetime can’t be the only dictating factor. We believe that the overall rate of reaction is dependent on many other parameters for the subsequent dark reaction steps as well.


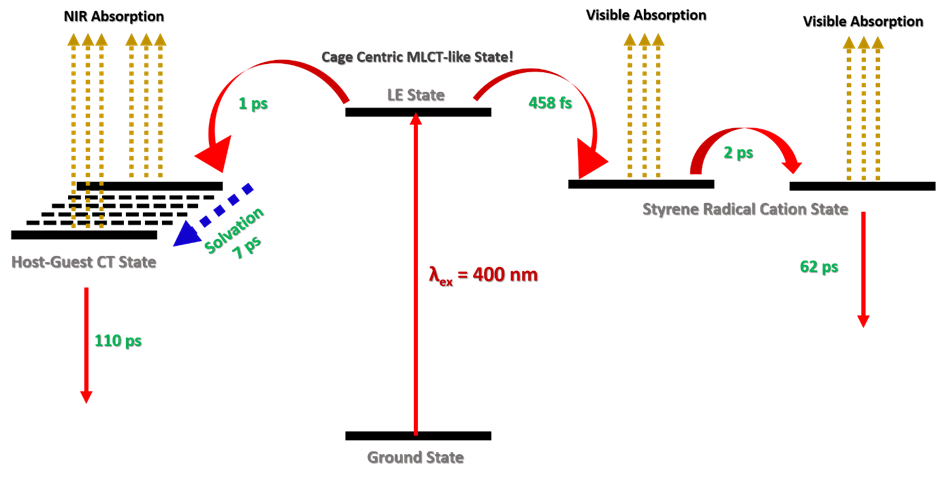


**Supplementary Figure 55.** The proposed kinetic scheme for the excited state dynamics of the **4-methystyrene** $\boldsymbol{\subset}$ **Encage**. The initial 400 nm excitation leads to formation of the 4-methylstyrene radical cation as well as the host-guest centric CT states.


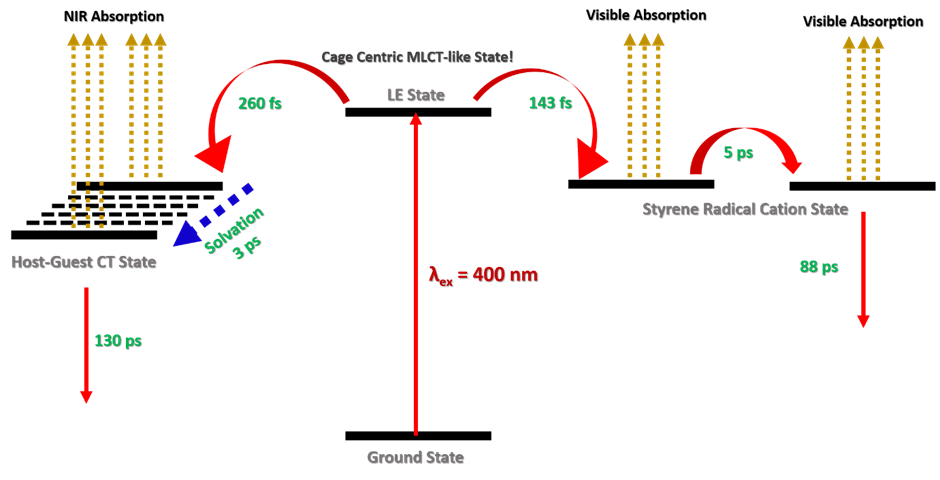


**Supplementary Figure 56.** The proposed kinetic scheme for the excited state dynamics of the **4-fluorostyrene** $\boldsymbol{\subset}$ **Encage**. The initial 400 nm excitation leads to formation of the 4-fluorostyrene radical cation as well as the host-guest centric CT states.


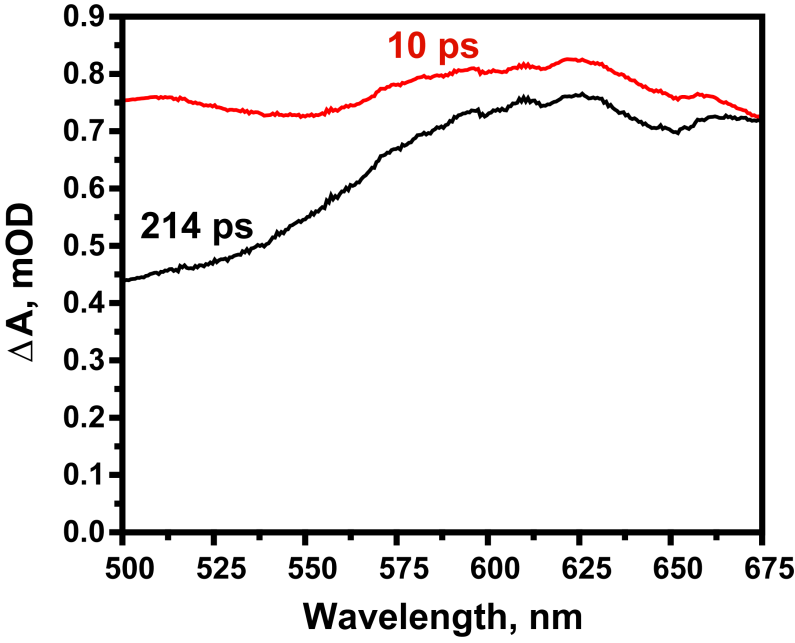


**Supplementary Figure 57.** Evolution associated spectra obtained from spectral deconvolution in the visible region of TA spectrum of **4-fluorostyrene** $\boldsymbol{\subset}$ **Encage** inclusion complex showing a 10 ps long species and 214 ps long species. Optimized two species sequential kinetic model (A$\to$B) was used for this global analysis and 0.2 ps was taken as the gaussian IRF.


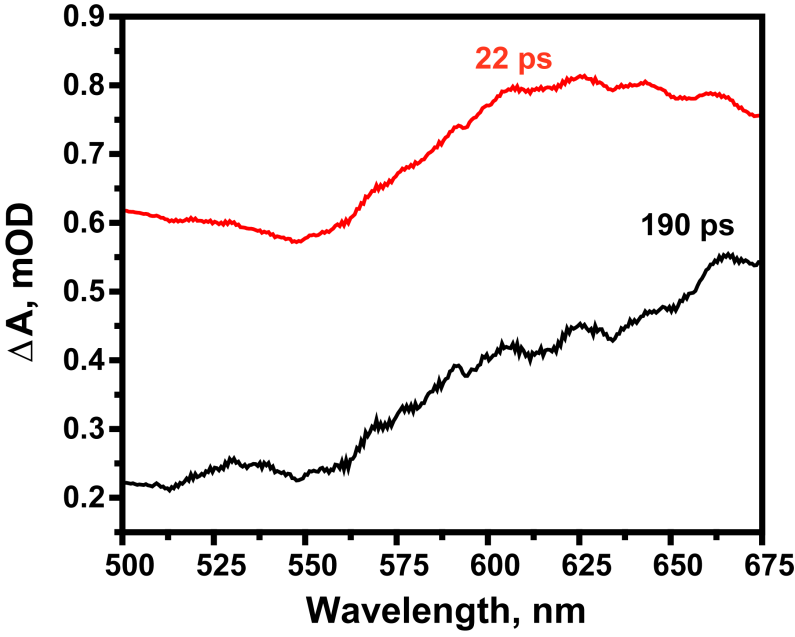


**Supplementary Figure 58.** Evolution associated spectra obtained from spectral deconvolution in the visible region of TA spectrum of **4-methylstyrene** $\boldsymbol{\subset}$ **Encage** inclusion complex showing a 22 ps long species and 190 ps long species. Optimized two species sequential kinetic model (A$\to$B) was used for this global analysis and 0.2 ps was taken as the gaussian IRF.


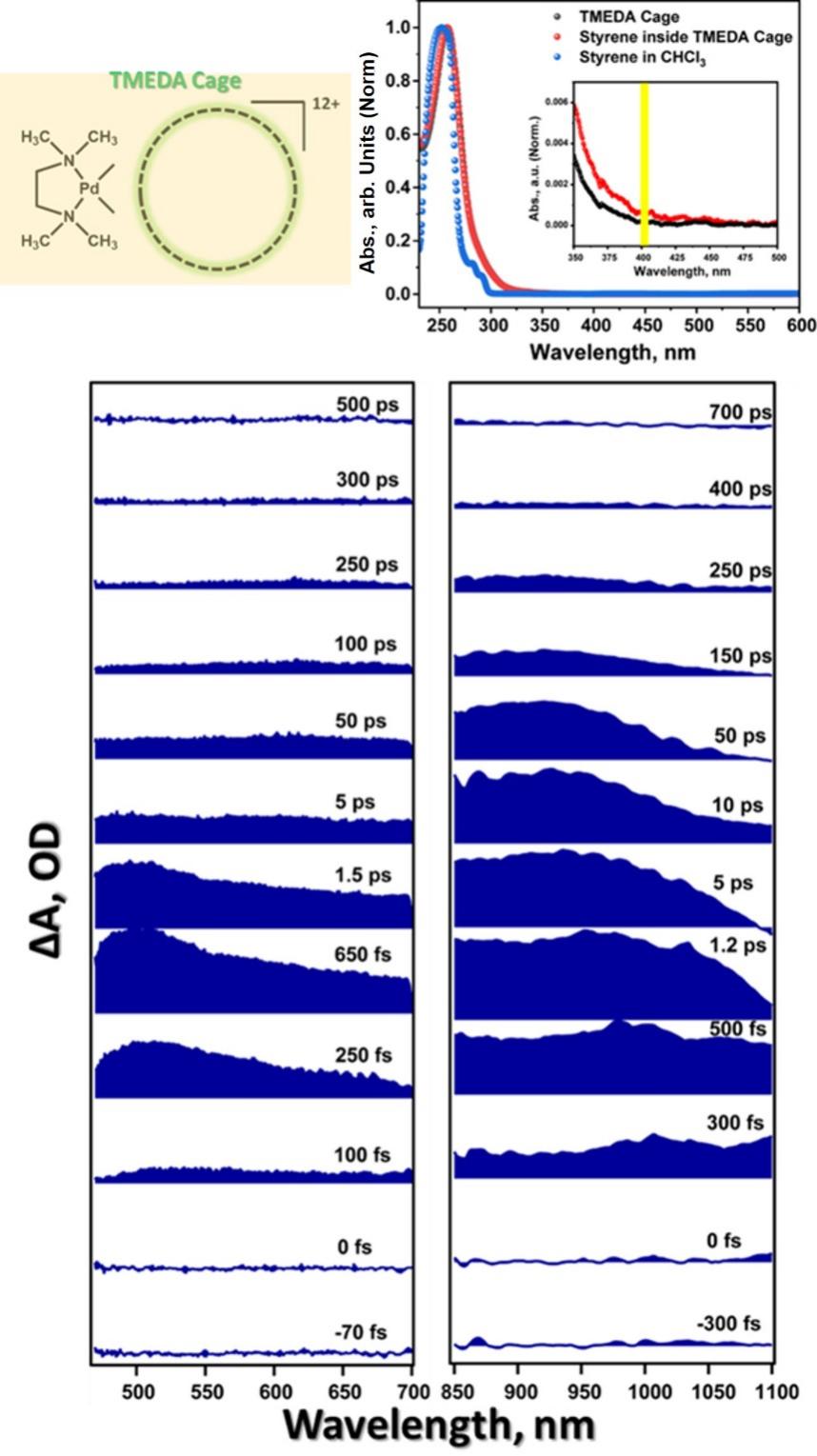


**Supplementary Figure 59.** Transient absorption spectrum for **styrene**$\boldsymbol{\subset}$**TMEDA cage** inclusion complex in both visible and NIR spectral region. Literature report suggests a broad absorption of styrene radical cation at 450 nm to 750 nm spectral region which we also observed in the same region suggesting the formation of styrene radical cation upon 400 nm light excitation. The spectral feature appearing at the NIR region shows a blue-shift of spectrum suggesting the formation of host-guest CT state prone towards showing solvation dynamics as the spectral movement.


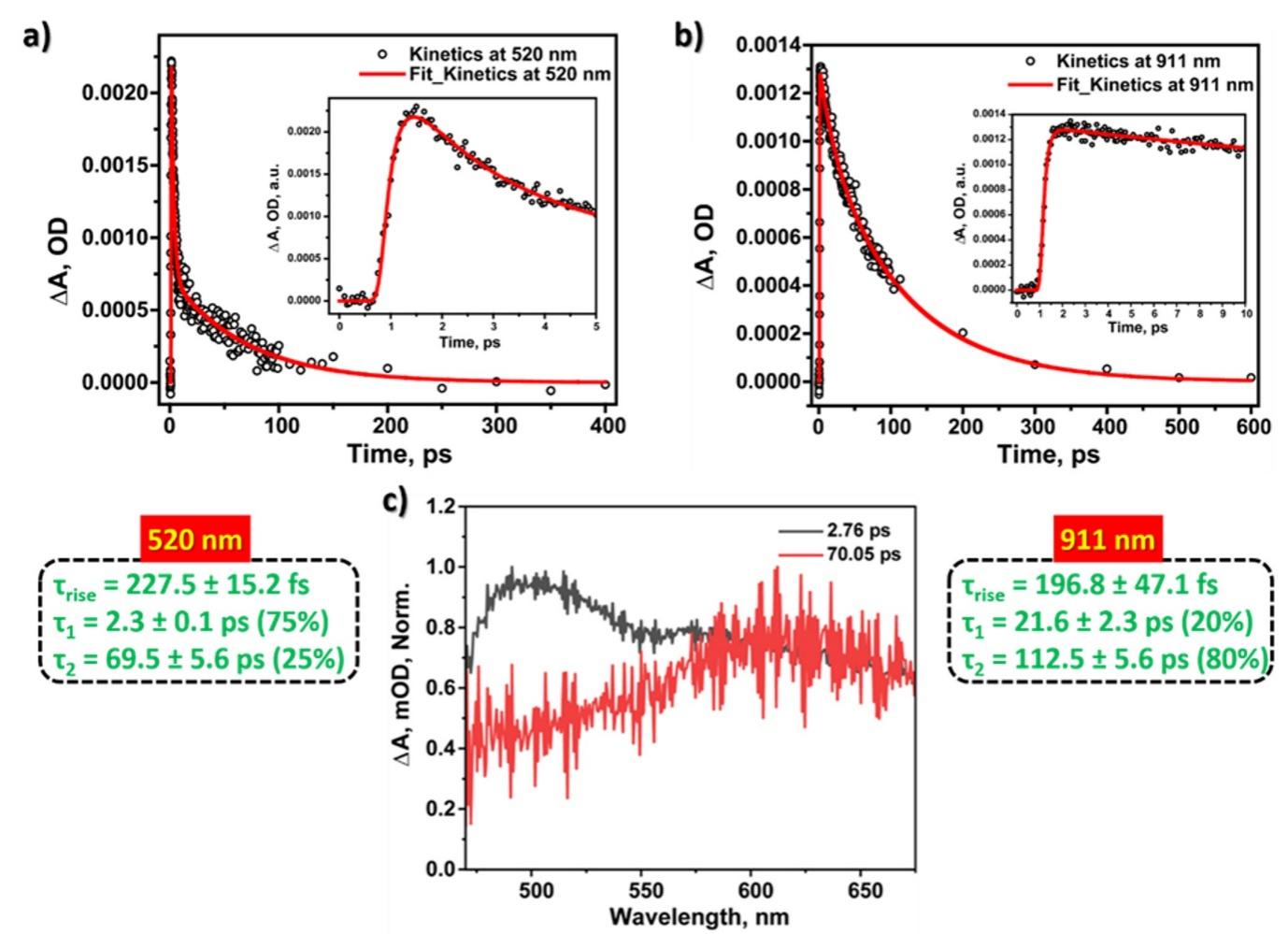


**Supplementary Figure 60.** Single point spectral kinetics (a) at 520 nm for styrene radical cation state absorption in the visible as well as (b) at 911 nm for **styrene ⊂ TMEDA cage** CT state absorption at NIR regime. The associated time components are mentioned accordingly. (c) SVD analysis of visible data matrix predicts the possible existence of one 3 ps long species and another 70 ps long species sequentially generated from the previous species.


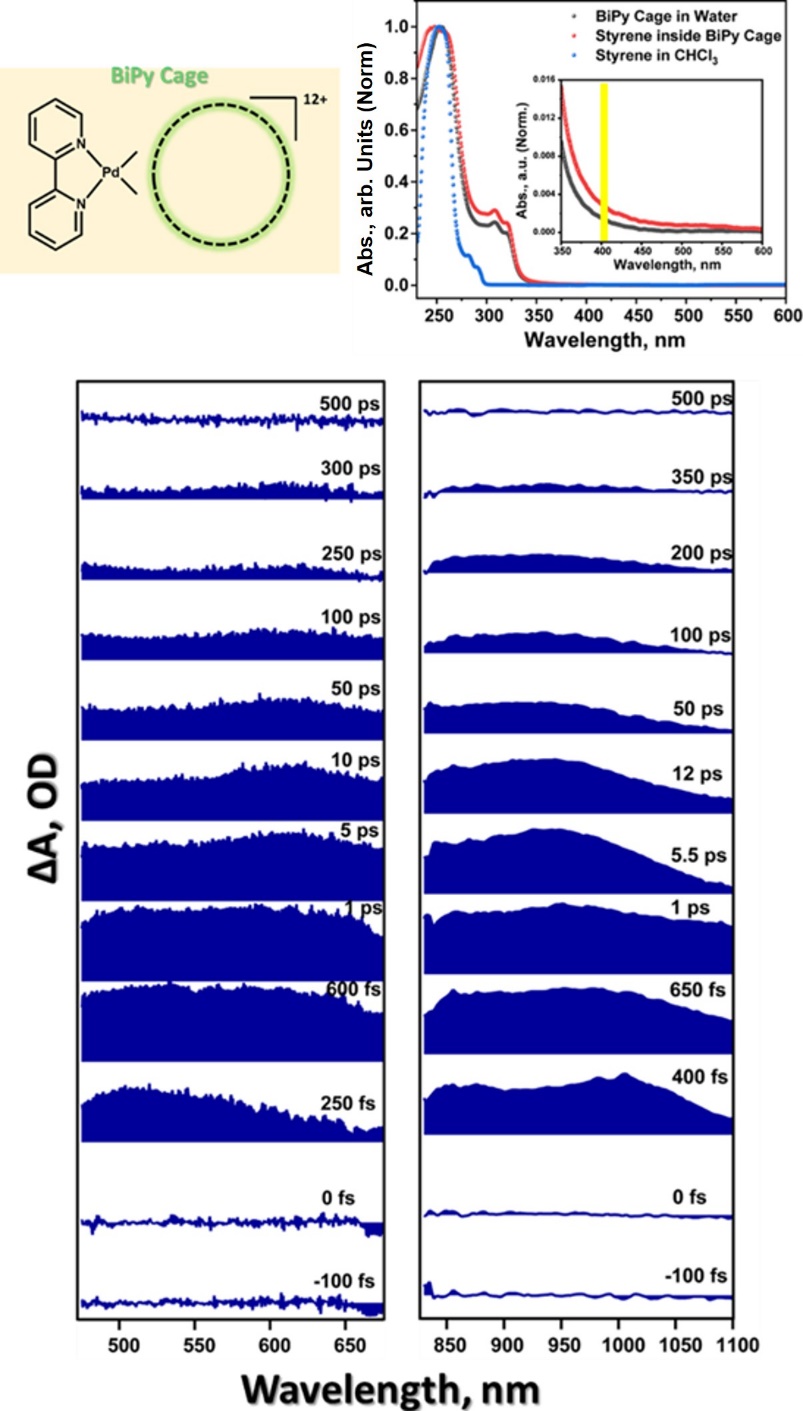


**Supplementary Figure 61.** Transient absorption spectrum for **styrene**$\boldsymbol{\subset}$**BiPy cage** inclusion complex in both visible and NIR spectral region. Literature report suggests a broad absorption of styrene radical cation at 450 nm to 750 nm spectral region which we are also observing in the same regime suggesting the formation of styrene radical cation upon 400 nm light excitation. The spectral feature appearing at the NIR region shows a blue-shift of spectrum suggesting the formation of host-guest CT state prone towards showing solvation dynamics as the spectral movement.


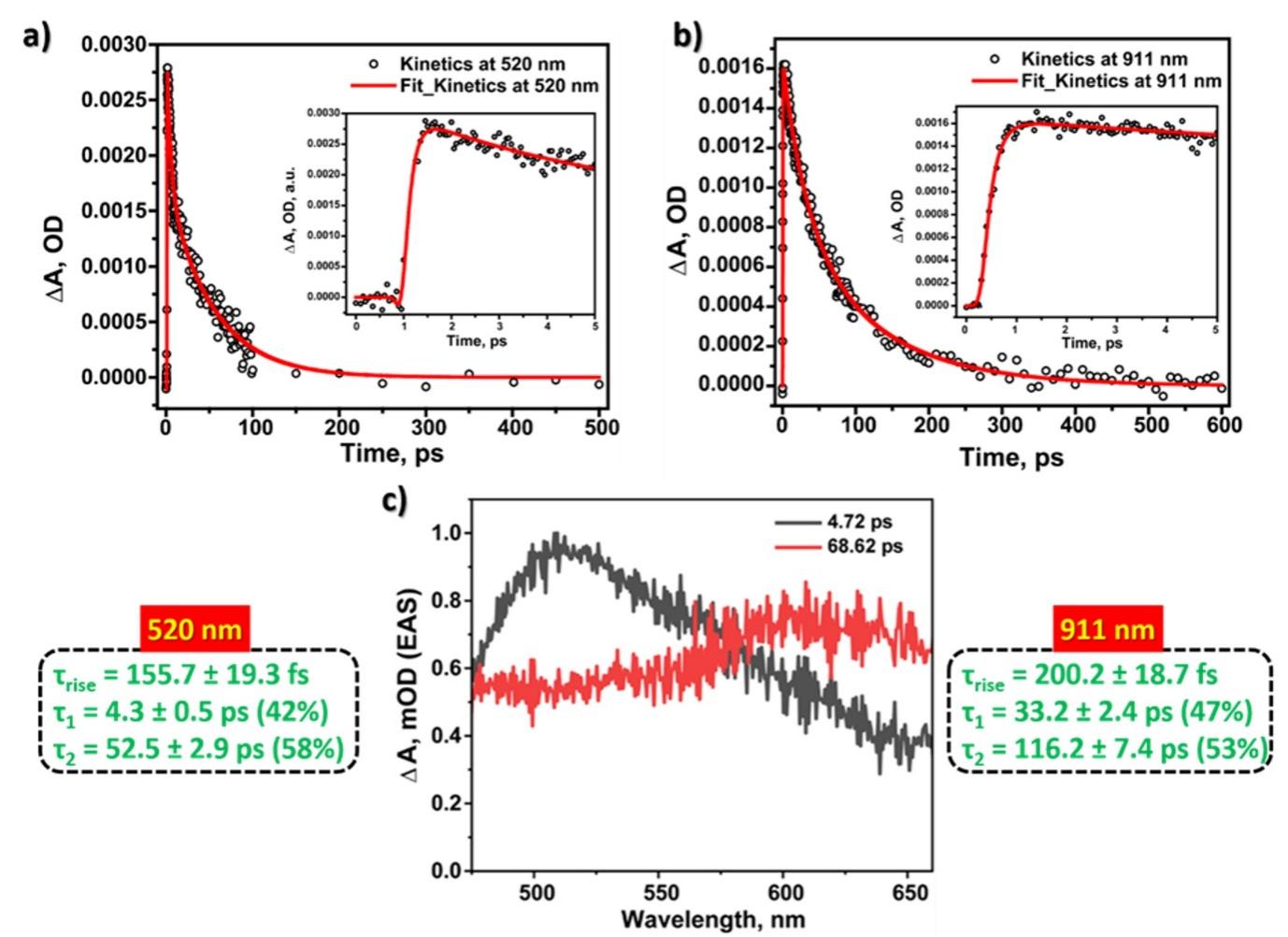


**Supplementary Figure 62.** Single point spectral kinetics (a) at 520 nm for styrene radical cation state absorption in the visible as well as (b) at 911 nm for **styrene ⊂ BiPy cage** CT state absorption at NIR regime. The associated time components are mentioned accordingly. (c) SVD analysis of visible data matrix predicts the possible existence of one ~ 5 ps long species and another 69 ps long species sequentially generated from the previous species.


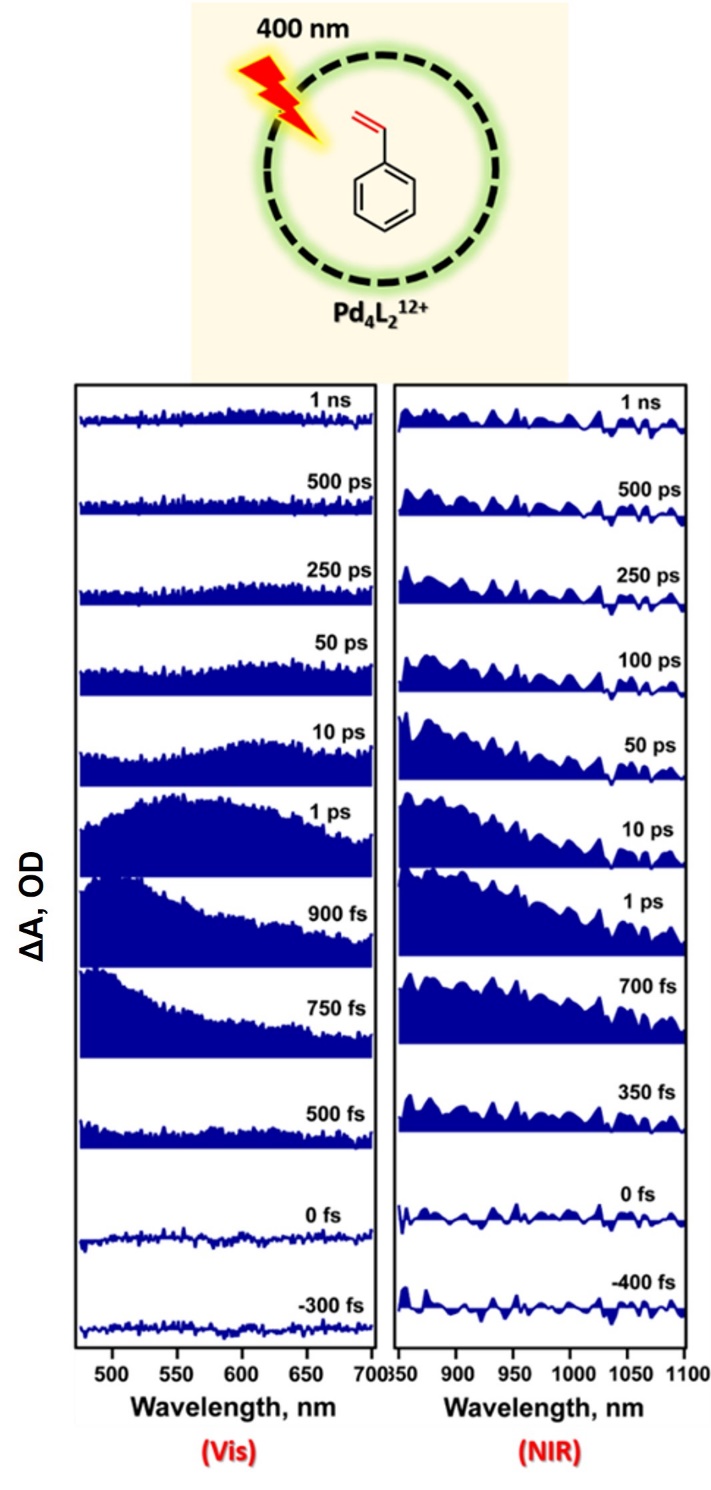


**Supplementary Figure 63.** Transient absorption spectra of **styrene ⊂ Pd_4_L’_2_^12+^ nanocage** complex at 400 nm light excitation. The visible spectral region arises due to the styrene radical cation formation while the NIR spectral regime denotes the host-guest coupled CT transitions. The corresponding excited state dynamics is shown for the whole spectral window of probing.


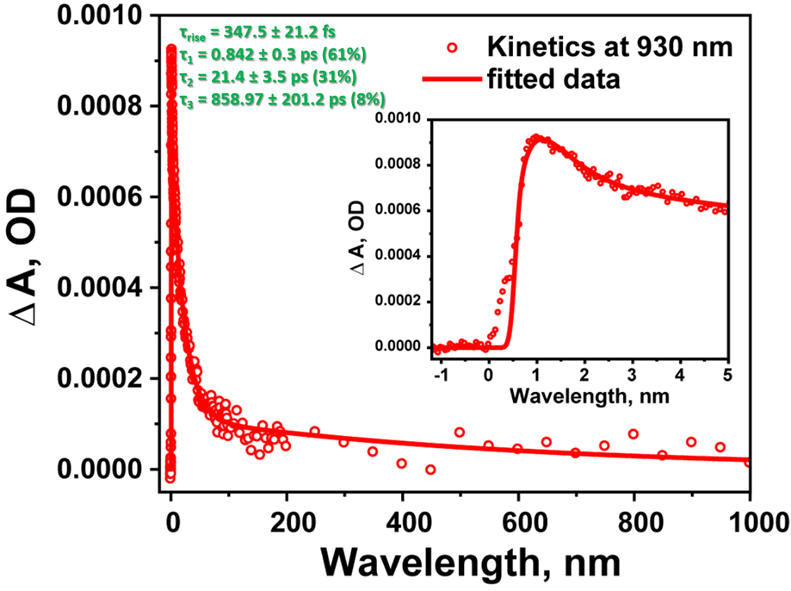


**Supplementary Figure 64.** Single point spectral kinetics of the transient absorption spectrum at 930 nm for **styrene ⊂ Pd_4_L’_2_^12+^ nanocage** inclusion complex which fits with 350 fs risetime and three decay components: 1 ps, 22 ps and 860 ps respectively.


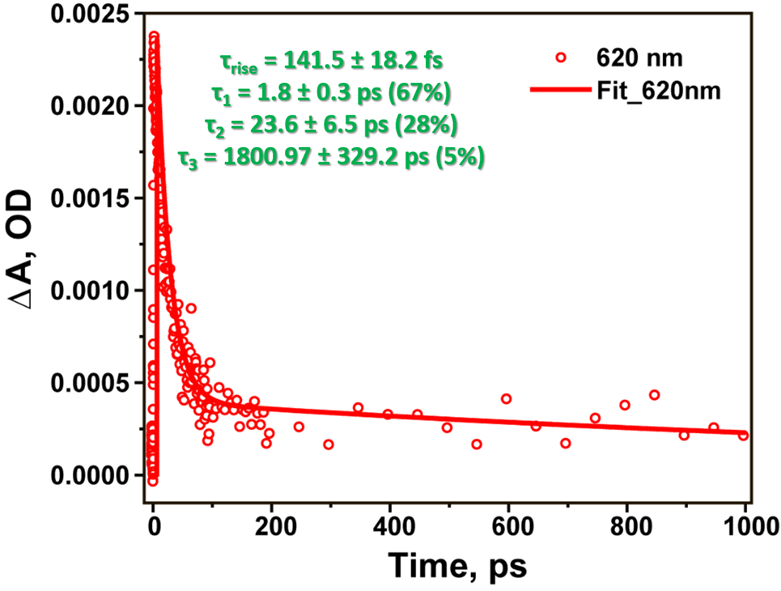


**Supplementary Figure 65.** Single point spectral kinetics of the transient absorption spectrum at 620 nm for **styrene ⊂ Pd_4_L’_2_^12+^ nanocage** inclusion complex which fits with 142 fs risetime (IRF limited) and three decay components: 2 ps, 24 ps and 1600 ps respectively.


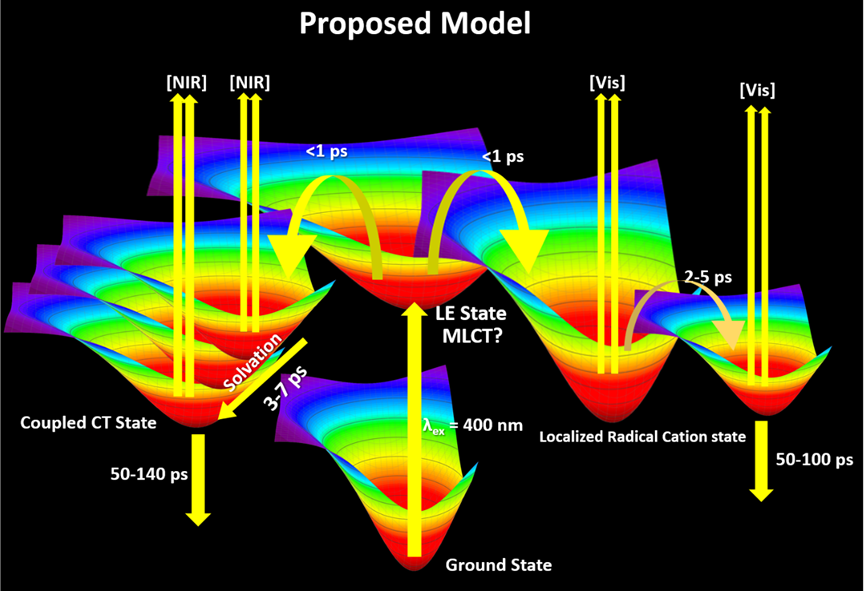


**Supplementary Figure 66.** Final plausible kinetic model for the excited state photophysics of styrene & its derivatives inside the Pd-nanocages (En, TMEDA, BiPy cages) involving a local cage centric excitation followed by population evolution to a host-guest coupled CT state and a localized guest radical cation state which are the possible photo-intermediates for the subsequent dark steps in photocatalysis.


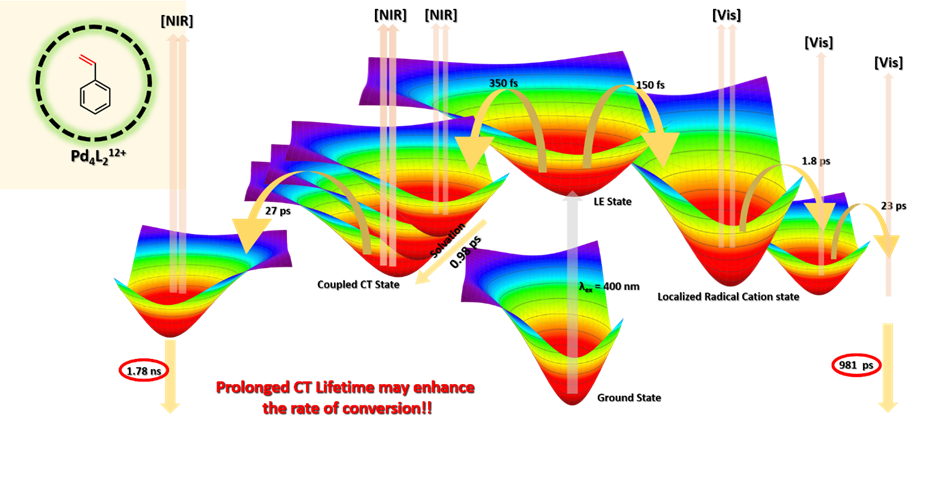


**Supplementary Figure 67.** Plausible kinetic model obtained from the spectral analysis of the transient absorption spectrum for **styrene ⊂ Pd_4_L’_2_^12+^ nanocage** inclusion complex throughout the spectral window.


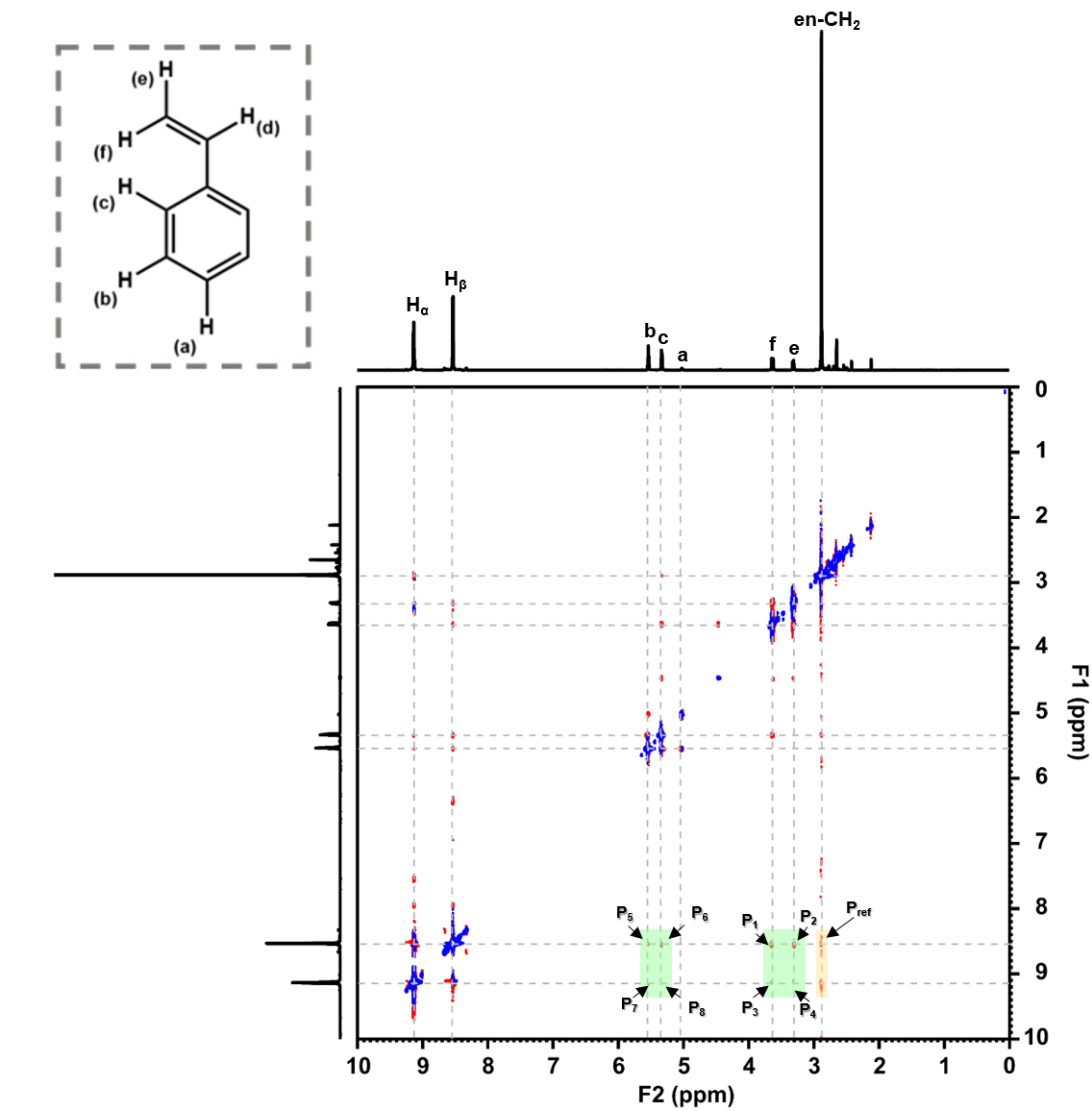


**Supplementary Figure 68.** ^1^H-^1^H ROESY spectrum of **styrene** $\boldsymbol{\subset}$ **Encage** complex (800 MHz, D_2_O, 298K); the P_ref_ was taken as the reference cross correlation peak originated due to the spatial dipolar coupling between H_α_ proton of the triazine wall of the En cage and the en-CH_2_ proton of the ancillary ligand. The known distance parameter in between these two protons (4.2 Å, calculated from the optimized geometry of En cage) was plugged in the equation 1 to calculate the other distance parameters in between different cage protons and styrene protons by simply comparing the volume integral values of those off-diagonal peaks (P_1_, P_2_, ….., P_8_) with that of the reference off-diagonal peak (P_ref_). The distance parameters helped us figure out the plausible host-guest packing model.

**Supplementary Table 1.** The relative distances between the triazine protons (H_α_ and H_β_) of **En-cage** and protons of the styrene host molecule. The labelling of guest styrene protons is shown in Supplementary Fig 68.

| Distances between protons | Calculated value (in Å) |
| --- | --- |
| **H_β_, e** | **2.75** |
| **H_β_, f** | **2.71** |
| **H_β_, b** | **4.52** |
| **H_β_, c** | **4.46** |
| **H_α_, e** | **2.96** |
| **H_α_, f** | **2.93** |
| **H_α_, b** | **4.41** |
| **H_α_, c** | **4.49** |


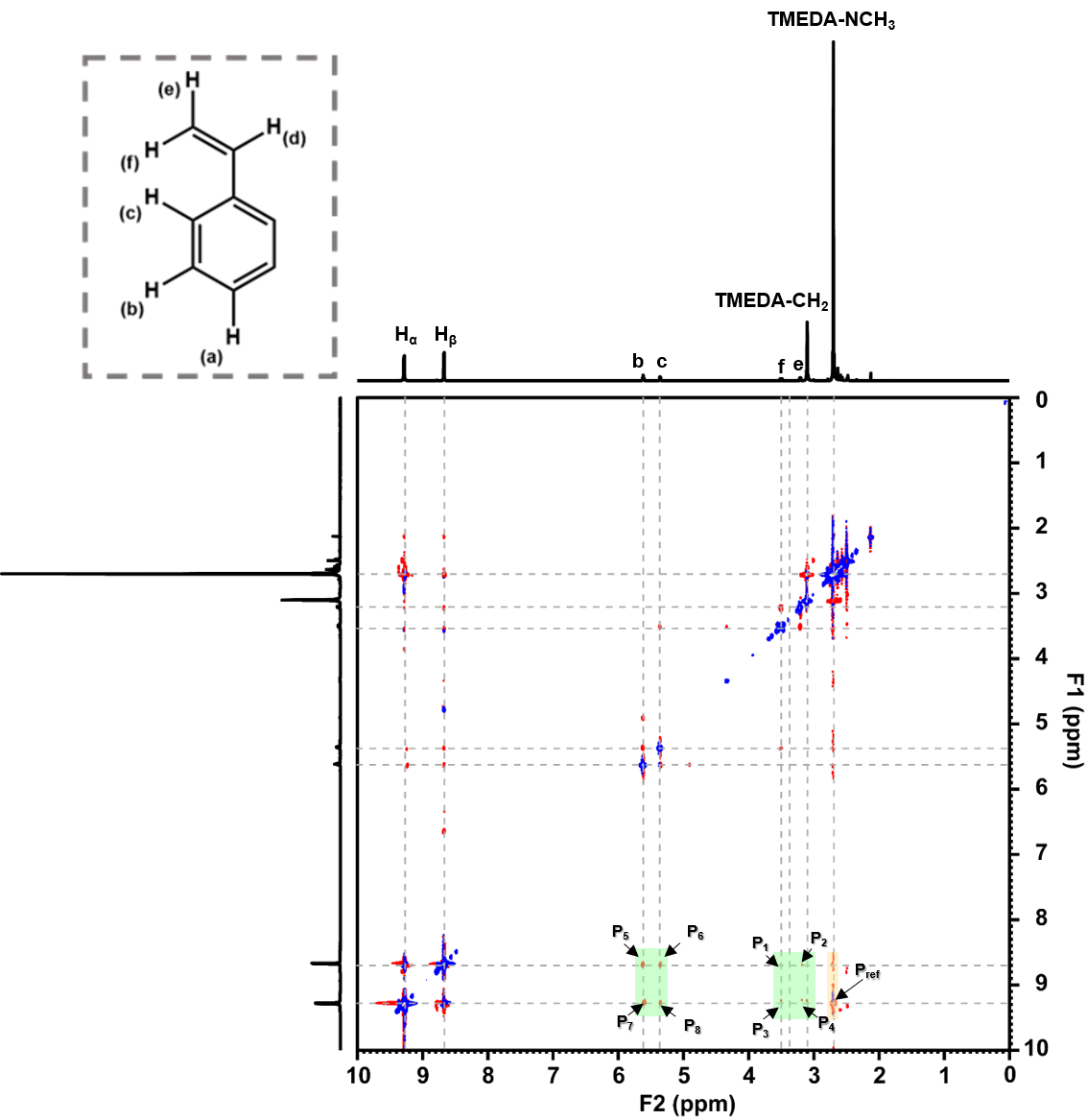


**Supplementary Figure 69.** ^1^H-^1^H ROESY spectrum of **styrene** $\boldsymbol{\subset}$ **TMEDA cage** complex (800 MHz, D_2_O, 298K); the P_ref_ was taken as the reference cross correlation peak originated due to the spatial dipolar coupling between H_α_ proton of the triazine wall of the TMEDA cage and the TMEDA-N(CH_3_)_2_ proton of the ancillary ligand. The known distance parameter in between these two protons (3.64 Å, calculated from the available crystal structure of TMEDA cage) was plugged in the equation 1 to calculate the other distance parameters in between different cage protons and styrene protons by simply comparing the volume integral values of those off-diagonal peaks (P_1_, P_2_, ….., P_8_) with that of the reference off-diagonal peak (P_ref_). The distance parameters helped us figure out the plausible host-guest packing model.

**Supplementary Table 2.** The relative distances between the triazine protons (H_α_ and H_β_) of **TMEDA** cage and protons of the styrene host molecule. The labelling of guest styrene protons is shown in Supplementary Fig 69.

| Distances between protons | Calculated value (in Å) |
| --- | --- |
| **H_β_, e** | **5.23** |
| **H_β_, f** | **5.98** |
| **H_β_, b** | **1.92** |
| **H_β_, c** | **2.07** |
| **H_α_, e** | **6.61** |
| **H_α_, f** | **6.26** |
| **H_α_, b** | **1.74** |
| **H_α_, c** | **2.12** |


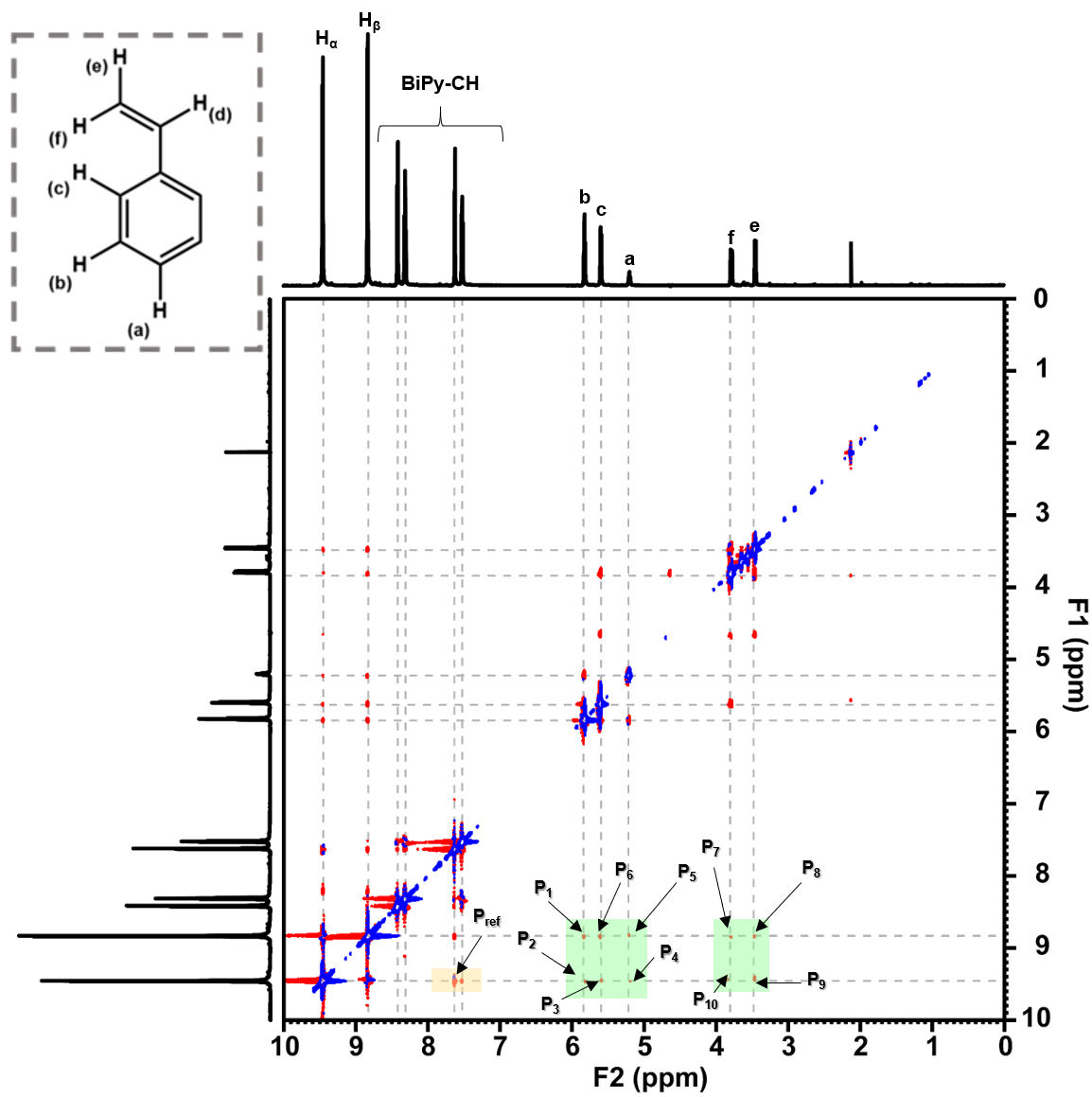


**Supplementary Figure 70.** ^1^H-^1^H ROESY spectrum of **styrene** $\boldsymbol{\subset}$ **BiPy cage** complex (800 MHz, D_2_O, 298K); the P_ref_ was taken as the reference cross correlation peak originated due to the spatial dipolar coupling between H_α_ proton of the triazine wall of the BiPy cage and the BiPy-aromatic CH proton of the ancillary ligand. The known distance parameter in between these two protons (3.39 Å, calculated from the available crystal structure of BiPy cage) was plugged in the equation 1 to calculate the other distance parameters in between different cage protons and styrene protons by simply comparing the volume integral values of those off-diagonal peaks (P_1_, P_2_, ….., P_10_) with that of the reference off-diagonal peak (P_ref_). The distance parameters helped us figure out the plausible host-guest packing model.

**Supplementary Table 3.** The relative distances between the triazine protons (H_α_ and H_β_) of **BiPy** cage and protons of the styrene host molecule. The labelling of guest styrene protons is shown in Supplementary Fig 70.

| Distances between protons | Calculated value (in Å) |
| --- | --- |
| **H_β_, e** | **4.79** |
| **H_β_, f** | **4.82** |
| **H_β_, b** | **4.76** |
| **H_β_, c** | **4.79** |
| **H_β_, a** | **4.89** |
| **H_α_, e** | **5.38** |
| **H_α_, f** | **5.43** |
| **H_α_, b** | **5.34** |
| **H_α_, c** | **5.37** |
| **H_α_, a** | **5.43** |


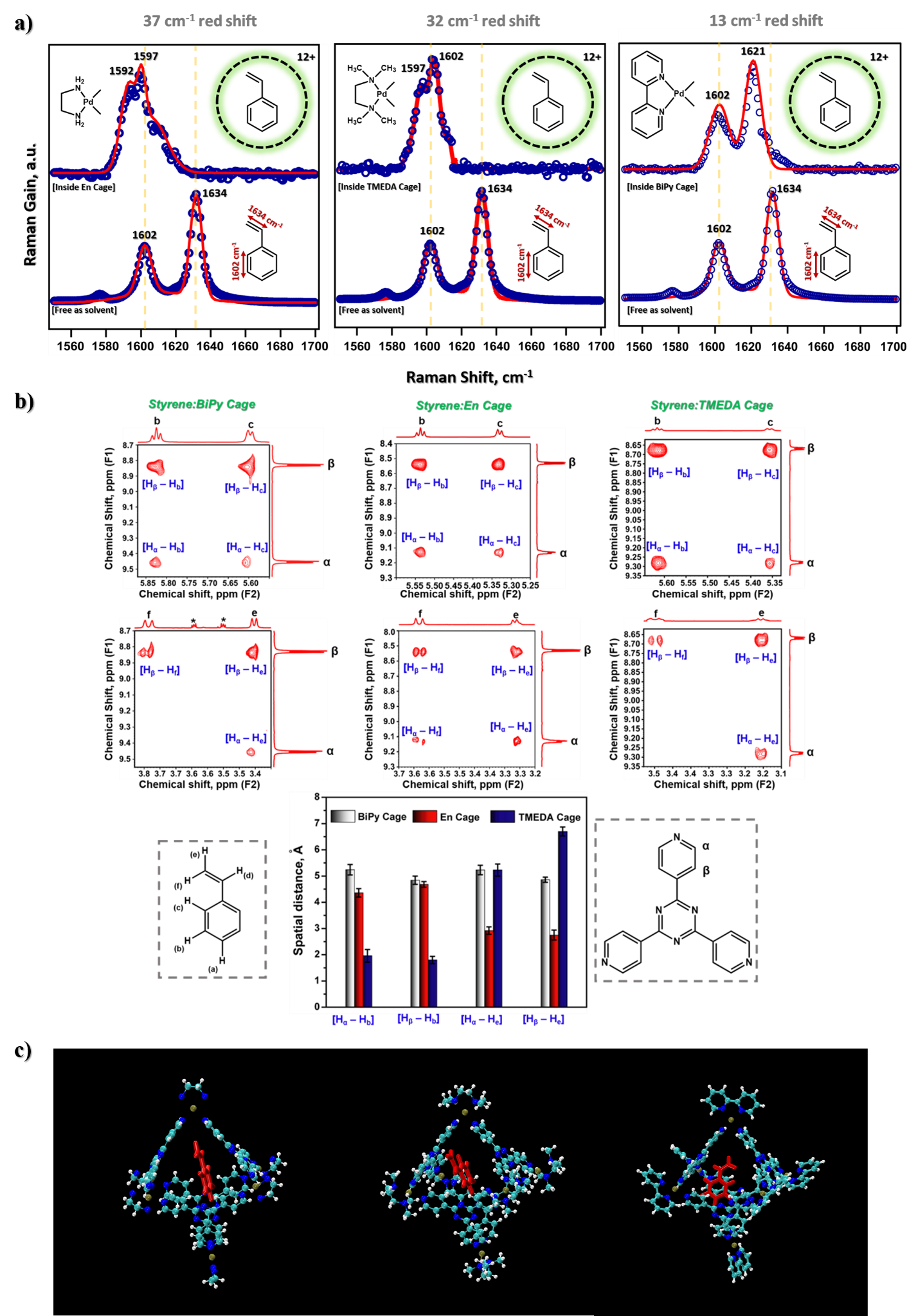


**Supplementary Figure 71.** The simplified schematic representation of host-guest packing models for styrene incarcerated inside the three different cages (En, TMEDA and BiPy, from left to right) which has been built from the distance parameters obtained from ROESY experiments.


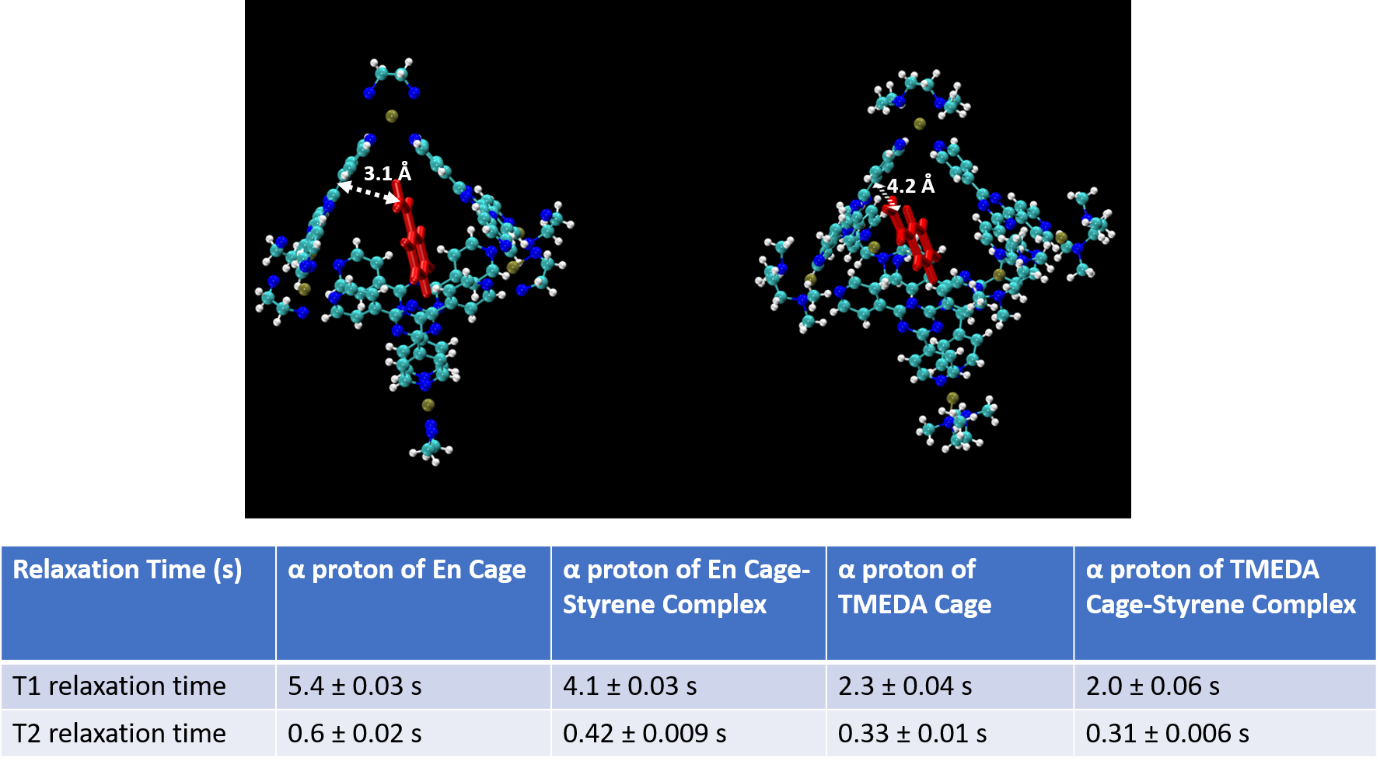


**Supplementary Figure 72.** Comparison of the influences of styrene incarceration inside nanocages on the T1 and T2 timescales of cage proton-Hα for En and TMEDA cages. For TMEDA cage, the corresponding timescales remain almost unperturbed upon styrene incarceration while for En cage, the corresponding timescales change considerably. This means that the styrene olefin site is more away from the triazine wall for TMEDA cage than En cage while the ROESY distance parameters also supports the argument. But when we tried to place two styrene molecules in one half of the cage symmetrically parallel to the opposite triazine walls maintaining the distance parameters obtained from ROESY data analysis, steric clash is happening for the geometric constraints. We found only one way to fit two styrene molecules by pointing those towards the pores. Also, for TMEDA cage the terminal protons of styrene olefin site are showing faster T1 relaxation (0.85 ms) timescale compared to Encage (1.1-1.2 ms) which additionally indicates towards more possible interactions of styrene olefin site with water molecules at the pores.


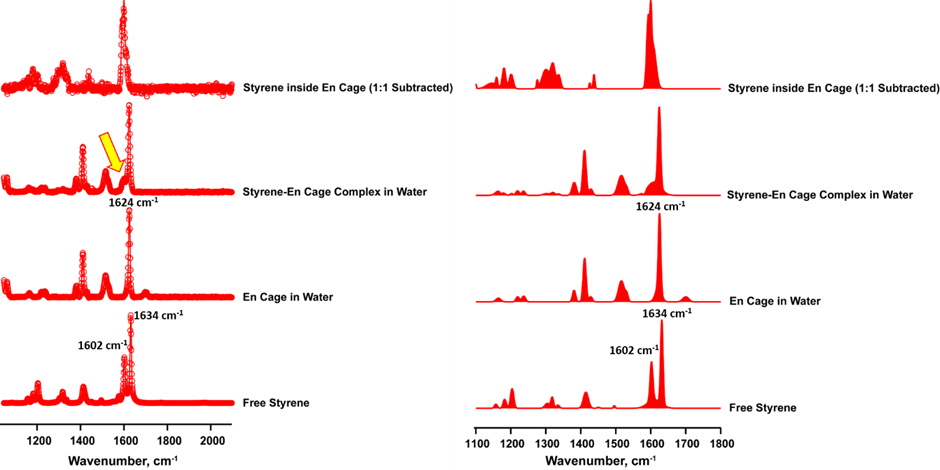


**Supplementary Figure 73.** The comparison of styrene vibrations in free solution versus inside hosts: The free styrene Raman spectra has been measured taking it neat as a solvent, the styrene-host complex Raman spectrum and the only host Raman spectrum were taken maintaining identical experimental conditions like concentration and laser power (5 mW). Then 1:1 subtraction has performed to extract only the styrene vibrational modes inside the cages. The demonstrated data processing was for styrene-En cage complex.


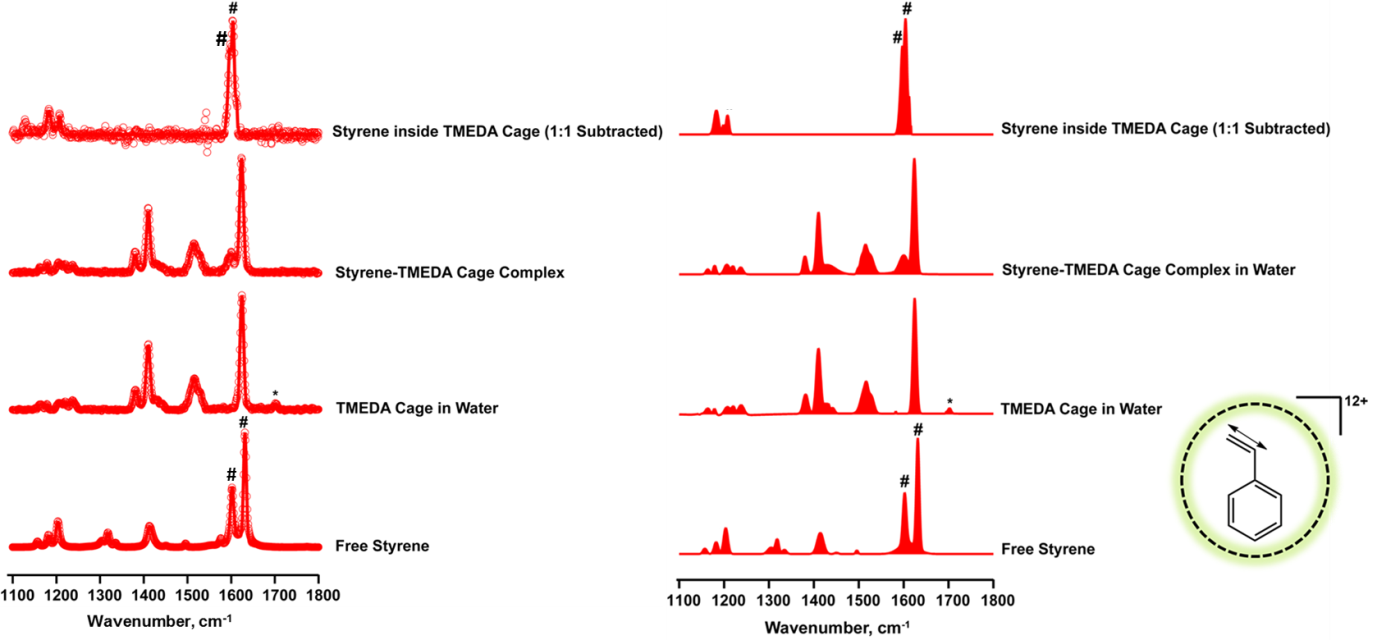


**Supplementary Figure 74.** Raman data for **styrene** $\boldsymbol{\subset}$ **TMEDA cage** complex to extract the bond-specific information of styrene inside TMEDA cage showing 32 cm^-1^ red shift of olefinic C=C stretch.


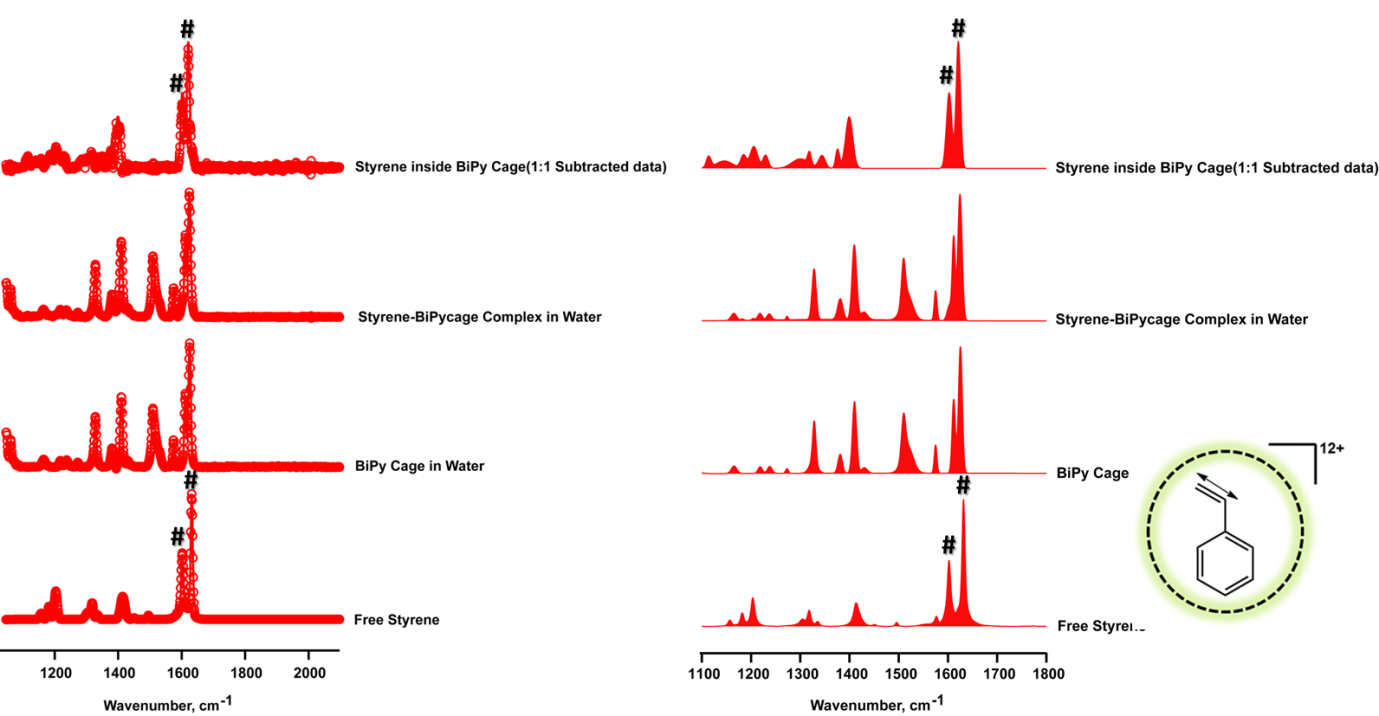


**Supplementary Figure 75.** Raman data for **styrene ⊂ BiPy cage** complex to extract the bond-specific information of styrene inside BiPy cage showing 13 cm^-1^ red shift of olefinic C=C stretch.


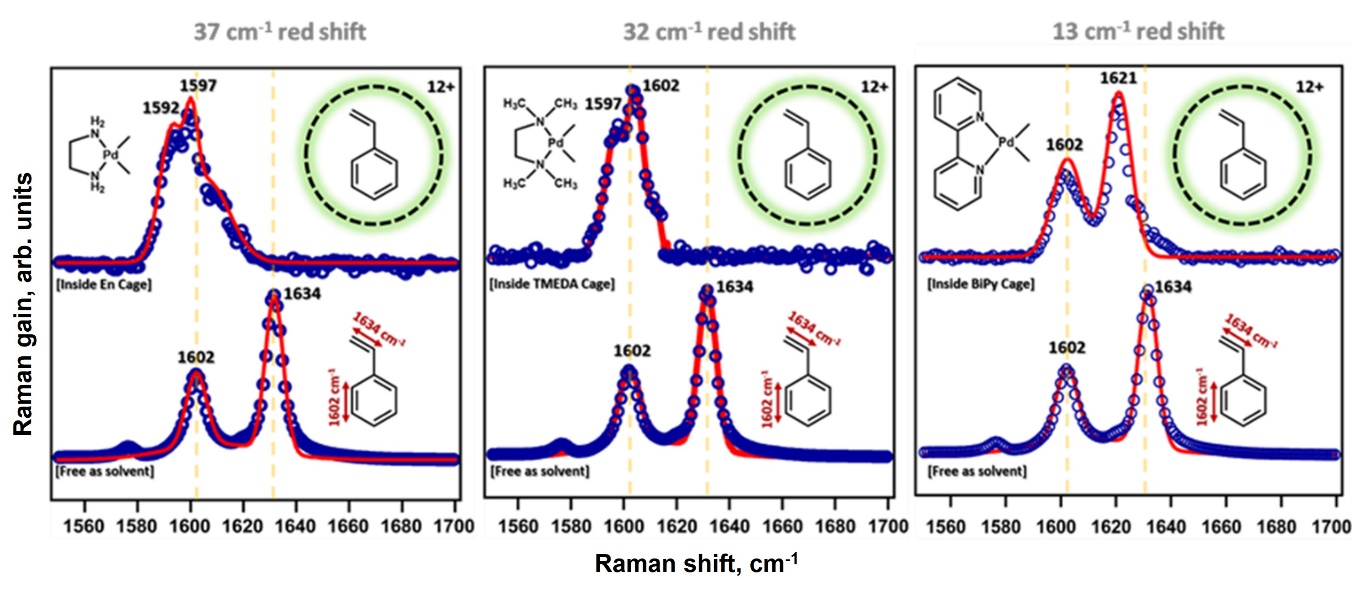


**Supplementary Figure 76.** Comparative Raman spectra of free styrene molecule and styrene molecule incarcerated inside different cages which clearly shows a prominent red shift for the olefinic C=C stretching mode and a very subtle red-shift of phenylic C=C stretching mode. The frequency shifts prove that even in the ground state configuration, styrene olefin moieties are polarized and weakened upon incarceration inside the cages bearing 12 positive charges. Such selective bond polarization might originate from the average positioning of styrene olefin bonds with respect to the electrostatic field generated by the positively charged Pd-ions at the cage structure and also from the possible electronic coupling in between host and guest electronic structure in tightly packed host-guest complexes. Further, the effect of subtly different guest preorganization in three different cages are also reflected in the different extents of olefinic bond polarization upon incarceration inside three cages.

**Supplementary References:**

1. Yoshizawa, M., Takeyama, Y., Kusukawa, T. & Fujita, M. Self-assembled coordination cages. *Analysis* **114**, 1347–1349 (2002).

2. Das, A., Mandal, I., Venkatramani, R. & Dasgupta, J. Ultrafast photoactivation of C-H bonds inside water-soluble nanocages. *Sci. Adv.* **5**, eaav4806 (2019).

3. Gera, R., Das, A., Jha, A. & Dasgupta, J. Light-induced proton-coupled electron transfer inside a nanocage. *J. Am. Chem. Soc.* **136**, 15909–15912 (2014).

4. Das, A., Jha, A., Gera, R. & Dasgupta, J. Photoinduced charge transfer state probes the dynamic water interaction with metal-organic nanocages. *J. Phys. Chem. C* **119**, 21234–21242 (2015).

5. Jha, A., Chakraborty, D., Srinivasan, V. & Dasgupta, J. Photoinduced charge transfer in solvated anthraquinones is facilitated by low-frequency ring deformations. *J. Phys. Chem. B* **117**, 12276–12285 (2013).

6. Ruckebusch, C., Sliwa, M., Pernot, P., de Juan, A. & Tauler, R. Comprehensive data analysis of femtosecond transient absorption spectra: a review. *J. Photochem. Photobiol. C: Photochem. Rev.* **13**, 1–27 (2012).

7. Berera, R., van Grondelle, R. & Kennis, J. T. M. Ultrafast transient absorption spectroscopy: principles and application to photosynthetic systems. *Photosynth. Res.* **101**, 105–118 (2009).

8. Che, J., Zhang, W. & Yan, Y. A classical time-frequency theory of transient absorption spectroscopy. *J. Chem. Phys.* **106**, 6947–6956 (2002).

9. Stein, T., Kronik, L. & Baer, R. Prediction of charge-transfer excitations in coumarin-based dyes using a range-separated functional tuned from first principles. *J. Chem. Phys.* **131**, 244119 (2009).

10. Prasad, S. *et al.* Near UV-Visible electronic absorption originating from charged amino acids in a monomeric protein. *Chem. Sci.* **8**, 5416–5433 (2017).
